# Mixed‐Functionalized Acylgermanes Result in Wavelength‐Controlled Fragmentation

**DOI:** 10.1002/anie.5559644

**Published:** 2026-04-03

**Authors:** André Culum, Manfred Drusgala, Roland C. Fischer, Anne‐Marie Kelterer, Mario Leypold, Dmytro Neshchadin, Michael Haas

**Affiliations:** ^1^ Institute of Inorganic Chemistry Graz University of Technology Graz Austria; ^2^ Institute of Physical and Theoretical Chemistry Graz University of Technology Graz Austria

**Keywords:** germanium, photochemistry, polymerization, radicals, synthesis design

## Abstract

Control over the radicals that a Type I photoinitiator releases without changing the formulation offers a direct handle on polymer microstructure. To address this challenge, molecular architectures with tailored solubility and reactivity are required. Consequently, we synthesized novel polyethylene glycol (PEG)‐functionalized acylgermanes via a straightforward one‐pot synthetic protocol in good yields, as confirmed by NMR spectroscopy, X‐ray crystallography and UV/Vis spectroscopy. By integrating PEG‐functionalized *para*‐alkoxybenzoyl substituents two different aryl‐carbonyl chromophores were introduced. UV/Vis spectroscopy and TD‐DFT (CAM‐B3LYP/def2‐TZVP) analysis assigned complementary bands to the mesitoyl and *para*‐alkoxybenzoyl units enabling wavelength‐selective excitation. Photo‐CIDNP tracked the fate of primary Ge/benzoyl radical pairs in the presence of a monomer quencher (butyl acrylate) and revealed competition between two alternative α‐cleavages via CIDNP polarizations of diagnostic aldehydes. By shifting the irradiation window, the balance between mesitoyl‐ and *para*‐alkoxybenzoyl‐derived radicals can be systematically tuned, an effect corroborated by steady‐state LED irradiation NMR experiments. Extension of this concept to other mixed tetraacylgermanes demonstrates that wavelength‐selective fragmentation is a general design principle rather than a compound‐specific anomaly. These PEG‐acylgermanes combine polar media compatibility with light‐programmable radical generation, converting small adjustments in wavelength into mechanistic control during photopolymerization.

## Graphical abstract

Selective radical generation from PEG‐acylgermanes is achieved by controlling the used wavelength. Distinct aryl‐carbonyl units undergo wavelength‐dependent α‐cleavage, producing radicals with different reactivities. UV/Vis, TD‐DFT, and photo‐CIDNP spectroscopy confirm that small adjustments in light input directly dictate radical identity and reactivity.

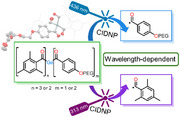

## 1 Introduction

**FIGURE 1:**
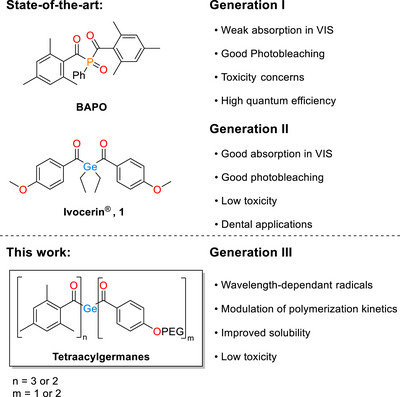
State‐of‐the‐art and this work.

Polymerization technology is intensively looking for greater control over when, where and how polymer networks are formed in order to advance sophisticated applications like multi‐material 3D printing, sequential curing of composites and micro‐patterned hydrogels, etc. [1, 2, 3, 4, 5, 6, 7]. Traditional photoinitiators (PIs) are generally “single‐channel”, they have one primary absorption band and one radical generation pathway. This means they respond to essentially one wavelength range and produce a set of fixed free radicals. When multiple reactions or stages are desired, multiple initiators or additives must be combined, which can introduce incompatibilities and added complexity. Developing new Norrish Type I photoinitiators that can undergo different α‐cleavages at different wavelengths would be a major advance for overcoming these limitations. Acylgermanes, germanium‐based photoinitiators, have attracted considerable interest in the field of photopolymerization owing to their strong visible‐light absorption capabilities and efficient radical formation [8, 9, 10, 11, 12]. Functioning as Type I photoinitiators, these compounds undergo homolytic bond cleavage upon UV or visible light irradiation, yielding highly reactive radicals that trigger the polymerization process. When compared to conventional photoinitiators such as acylphosphine oxides [13, 14, 15] or benzoin‐based derivatives [16], acylgermanes offer several notable advantages, including stronger absorption in the visible‐light range, no toxicity problems, and high quantum efficiencies for radical generation [17, 18, 19, 20]. In general, their synthesis can be approached via two principal routes: (i) direct acylation of germanide intermediates [21, 22, 23, 24, 25, 26, 27, 28] and (ii) functional group transformations of pre‐existing germanium‐containing compounds [29, 30, 31, 32, 33, 34] (see Figure 1 for the state‐of‐the‐art).

Applications range from 3D printing, dental applications [21, 29], wavelength orthogonal polymerization [35] and surface modification [34, 36]. Despite these advantages, less attention has been paid to the fine‐tuning of their excited‐state properties for advanced wavelength dependent applications. Bestowing these compounds with the ability to modulate polymerization kinetics via selective radical formation would allow for their use in spatiotemporally demanding polymer applications such as multi‐material 3D printing [37] holographic and volumetric photopolymerization [38] as well as photolithography in microfabrication [39].

Throughout our investigation of acylgermanes, a central question emerged: how does the incorporation of two discrete chromophores influence the photochemical behavior of these compounds? Specifically, can radical formation be selectively initiated at different wavelengths? To address this, we aimed to design photoinitiators with tunable photochemical properties, enabling control over radical generation via wavelength selection. By employing the mesitoyl‐substituted germenolates developed in our group and utilizing the insights gained from the Ivocerin (**1**) synthesis [30, 31], we have now identified two key structural elements with distinct electronic properties that could enable wavelength‐dependent activation (see Figure 1, this work).

This also aligned with our goal of modifying the solubility properties of these compounds. Instead of employing a simple methoxy group we designed compounds with polyethylene glycol moieties, allowing for better solubility in polar solvents like acetonitrile and methanol. Previously, we addressed this limitation by introducing d‐galactose substituents, which significantly enhanced solubility in polar media. However, this approach required a multi‐step synthetic route that hindered further development and broader application of these compounds [28]. This hurdle could be overcome with a shorter synthetic route enabled by the use of PEG moieties. The PEG units were designed to enhance polarity and compatibility with polar media while also serving as the *para*‐alkoxy substituent, thus addressing solubility limitations and the need for wavelength‐selective radical generation in a single modification. We selected two commercially available PEG derivatives containing either three or four ethylene oxide units, anticipating that their incorporation would eliminate the need for complex multi‐step synthesis and provide opportunities to modulate excited‐state reactivity and radical formation through strategic substitution.

## 2 Results and Discussion

### 2.1 Synthesis of PEG Substituted Diacylgermanes

**SCHEME 1:**
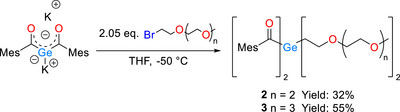
Synthetic route toward the PEG substituted diacylgermanes **2**, **3**.

We decided to use the geminal bisenolate salt K_2_Ge[(CO)Mes]_2_ as our germanium building block for the synthesis of our diacylgermanes [26]. In both cases, the bisenolate was reacted with 2.05 equivalents of the respective polyethylene glycol bromide. After the reaction and subsequent aqueous work‐up, the crude reaction mixtures were purified via column chromatography in moderate to good yields (see Scheme 1). Experimental details and NMR spectra of the isolated products are summarized in the *Experimental Section* of the Supporting Information.

### 2.2 Synthesis of PEG Substituted Triacylgermanes

**SCHEME 2:**
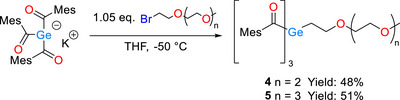
Synthetic route toward the PEG substituted triacylgermanes **4**, **5**.

In order to synthesize PEG substituted triacylgermanes, we opted for our well‐established triacylgermenolate as the starting material for our manipulations [23]. Again, in both cases the enolates were reacted with 1.05 equivalents of the respective polyethylene glycol bromide. After the reactions and subsequent aqueous work‐up, the crude reaction mixtures were isolated via column chromatography in good yields (compare Scheme 2). Experimental details are summarized in the *Experimental Section* of the Supporting Information.

### 2.3 Synthesis of PEG Substituted Tetraacylgermanes

**SCHEME 3:**
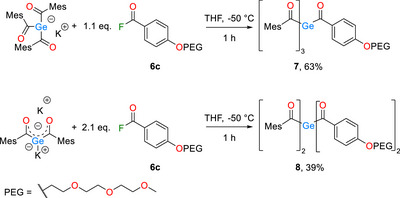
Synthesis of PEG substituted tetraacylgermanes.

**FIGURE 2:**
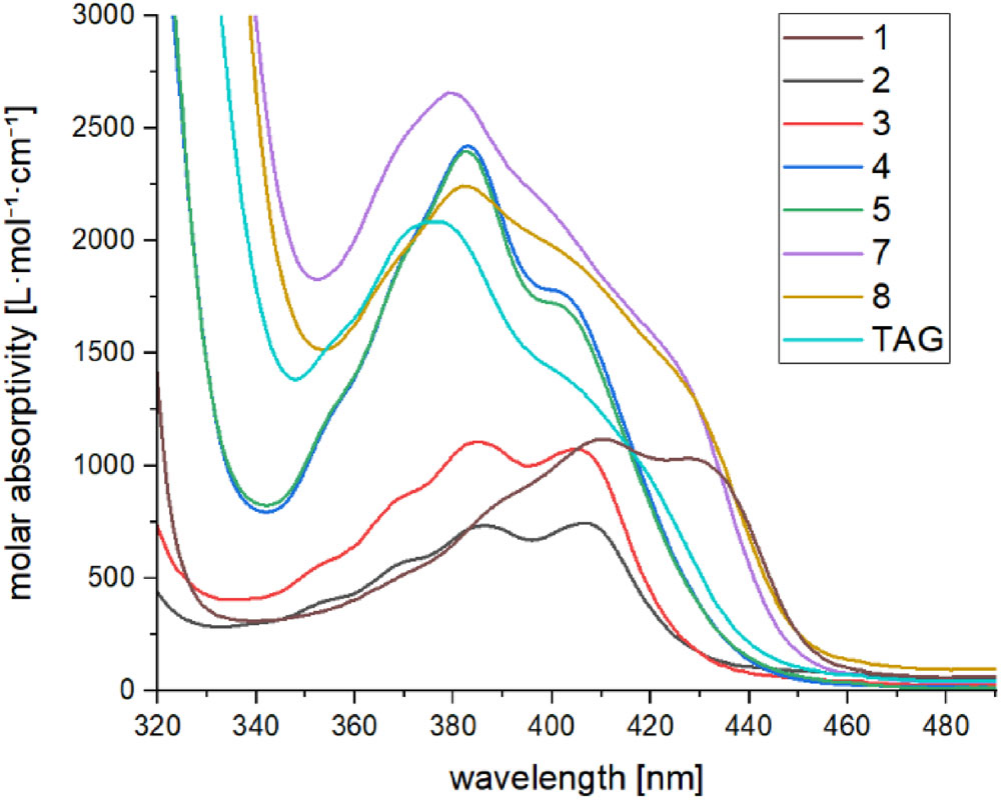
UV/Vis spectra of **1–5**, **7**, **8** and TAG in THF.

The freshly prepared acid fluoride **6c** (Supporting Information Scheme S1) was then reacted with the triacylgermenolate and the bisenolate, yielding the tetraacylgermanes **7** and **8** respectively in excellent and in good yields, respectively (Scheme 3). Important to note here is the design choice to utilize an aromatic moiety as a spacer group. This was done with the goal of utilizing the PEG groups electron donating properties to further improve the spectroscopic properties of the target compounds **7** and **8**. The effects of this substitution will be discussed in the UV/Vis part of the manuscript (see Figure 2).

### 2.4 UV/Vis Spectroscopy

A fundamental step in investigating photochemical activity is the determination of the absorption properties by using UV/Vis spectroscopy. THF was chosen as the solvent due to the good solubility of all derivatives. Moreover, as a very polar solvent it also mimics polarity of the target environments. The UV/Vis spectra of the isolated derivatives in comparison with Ivocerin (**1**) and tetramesitoylgermane (TAG) are presented in Figure 2. For clarity we discuss the spectra according to the number of acyl groups attached to the germanium center. The absorption profiles of **2** and **3**, as examples of diacylgermanes, exhibit a significant hypsochromic shift in respect to **1**.

This trend is consistent with the established behavior of this compound class: di‐ortho substitution on the aryl groups reduces effective conjugation and produces a significant hypsochromic shift compared with unsubstituted aryl substituents [22]. More surprising are the high extinction coefficients of the triacylgermanes **4** and **5**, which even exceed those of tetramesitoylgermane. We speculate that the PEG groups exhibit an inductive effect that increases the extinction coefficient. A similar trend is also seen for the tetraacylgermanes **7** and **8**. Finally, for the tetraacylgermanes **7** and **8** a significant broadening of the n/σ−π* absorption band is observed, which spans from 350 to 460 nm. This can be seen as the combination of the absorption behavior of the two different acyl substituents.

**FIGURE 3:**
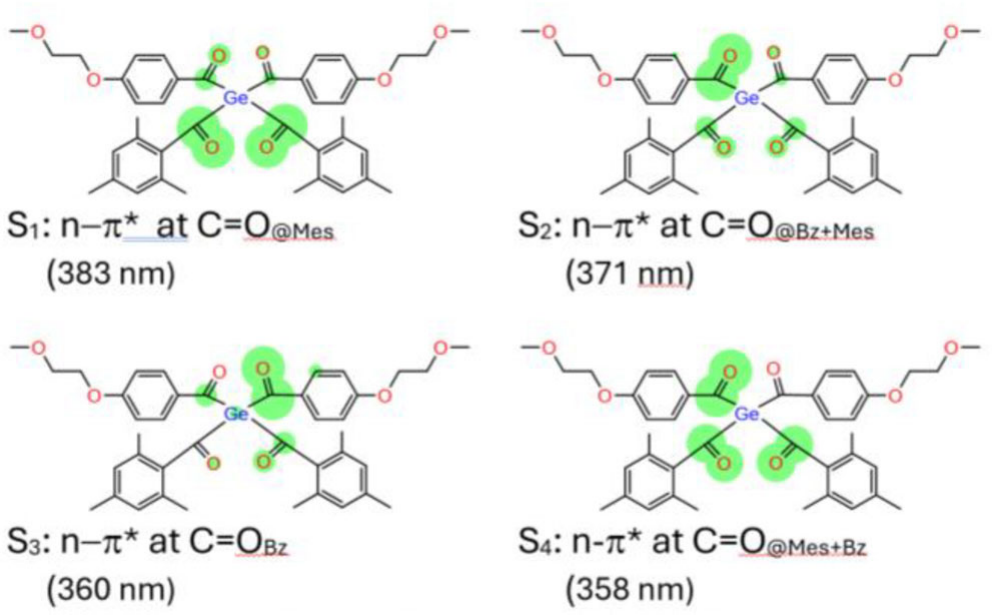
Sketch of the Natural Transition Orbital (NTO) difference densities for the four lowest vertical excitations of the long‐wavelength bands of a model of **8**. The size of the green circles is proportional to the size of the NTO difference densities between the ground and excited states. CAM‐B3LYP/def2‐TZVP calculated absorption wavelengths are given in parenthesis.

DFT calculations on compound **8** confirm that the absorption band arises from four vertical excitations corresponding to n−π* transitions from the frontier orbitals. Natural transition orbital (NTO) density analysis clarifies the composition of these excitations: the long‐wavelength edge of the band (S_1_) is dominated by transitions at the carbonyl groups of the mesitoyl moieties, with a smaller contribution from the p‐alkoxy benzoyl carbonyls. The central region of the band (S_2_‐S_3_) shows a significant increase in transition density localized on the benzoyl C═O groups. Finally, the blue edge of the band (S_4_) originates from contributions of both mesitoyl and p‐alkoxy benzoyl carbonyl groups. These results are illustrated in Figure 3 while detailed information on the vertical excitations and corresponding NTOs is provided in the Supporting Information. At first glance, these calculations appear to contradict the opening statement that mesitoyl substituents should dominate the blue edge, whereas *para*‐alkoxybenzoyl groups should dominate the red edge. However, molecules bearing two different aryl–carbonyl chromophores have not yet been examined. Surprisingly, we find that the distinct carbonyl‐centered manifolds can combine in ways that produce an apparent edge inversion. Moreover, the absorption pattern of **8** indicates that excitation can occur over a broad wavelength range, which in principle could influence the relative contributions of competing cleavage pathways leading to distinct benzoyl‐type radicals. However, the absorption spectrum alone is not sufficient to predict photochemical selectivity. The effective rate of a photochemical process at a given wavelength is determined by the product of the wavelength‐dependent absorption and the corresponding quantum yield (i.e., the photochemical action plot). Consequently, although the absorption profile of **8** suggests that wavelength‐dependent excitation may influence the balance between the competing cleavage channels, the actual product distribution will ultimately depend on the wavelength dependence of the quantum yields of the respective processes. This principle has been emphasized in recent discussions of precision photochemistry [7]. Comparison of the *para*‐alkoxybenzoyl‐substituted compound **8** with the benzoyl‐substituted analogue (see Supporting Information Table S8 for details) shows that introduction of the alkoxy group significantly shifts electron density from the phenyl ring toward the adjacent carbonyl group, resulting in a blue shift of the absorption bands S_2_ and S_3_. In other words, the lowest energy absorption bands in **8** correspond to mesitoyl groups while next higher energy bands are dominated by the *para*‐alkoxybenzoyl moieties.

### 2.5 X‐Ray Crystallography

**FIGURE 4:**
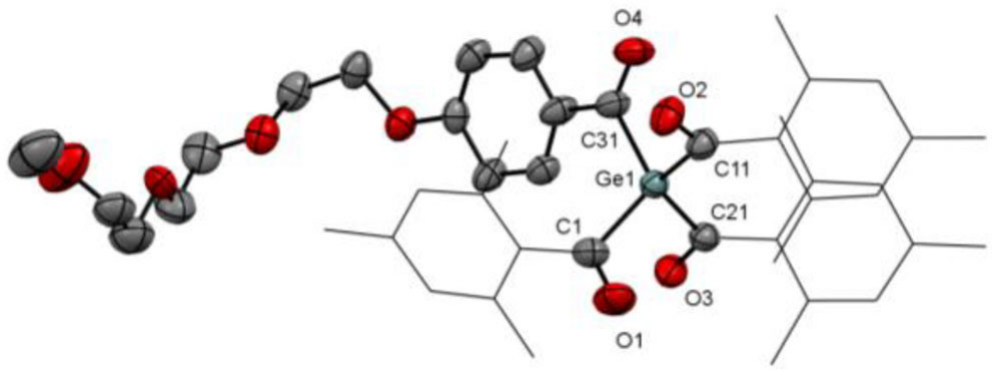
ORTEP representation of compound **7**. Thermal ellipsoids are drawn at the 50% probability level. Hydrogen‐atoms are omitted for clarity and the mesitoyl groups are presented in wireframe style. Selected bond lengths [Å] with estimated standard deviations: Ge(1)‐C(1) 2.032(3), Ge(1)‐C(11) 2.040(3), Ge(1)‐C(21) 2.029(3), Ge(1)‐C(31) 2.023(3), O(4)‐C(31) 1.224(3), O(1)‐C(1) 1.209(4).

Crystals suitable for single‐crystal XRD were obtained for compound **7** and **6b**. The structure of **7** is described below (Figure 4), while the structure of carboxylic acid **6b** can be found in the Supporting Information (Figure S50). Both single crystals were grown by cooling concentrated solutions in diethyl ether to −30 °C. The solid‐state structure of **7**, reveals a tetraacylgermane motif with the germanium center Ge(1) coordinated by four carbon atoms from acyl substituents in a slightly distorted tetrahedral geometry. The Ge–C bond lengths fall in a narrow range from 2.023 to 2.040 Å, consistent with typical Ge–acyl carbon bonds [8]. The carbonyl functionalities are clearly defined, with C═O bond lengths characteristic for localized ketone double bonds and showing no indication of significant delocalization toward the germanium atom. The mesitoyl substituents adopt orientations that minimize steric congestion, leading to slight deviations from ideal tetrahedral angles but without inducing significant strain at the metal center. More information about the crystal structures is provided in the Supporting Information (Table S12).

### 2.6 CIDNP and QM Calculations

To investigate the mechanism of the primary photo‐induced reactions of acylgermanes **2 ‐ 8**, we utilized ^1^H Photo‐CIDNP [40, 41] (Photo‐Chemically Induced Dynamic Nuclear Polarization) spectroscopy under various conditions, in combination with DFT quantum mechanical calculation. Photo‐CIDNP is an NMR‐based technique in which radical pairs are generated—typically by light irradiation—within the strong magnetic field of an NMR magnet. Once formed, the radicals of the primary pair undergo further reactions leading to diamagnetic products.

Two principal reaction pathways are available to the primary radicals: (1) recombination within the solvent cage (“cage” reaction), and (2) diffusion apart followed by reactions in the bulk (“escape” reactions). The likelihood of each pathway is governed by the g‐factor difference between the radicals and, via hyperfine interactions, by the nuclear spin states of the NMR‐active nuclei involved.

As a result of this nuclear spin‐selective process, non‐Boltzmann nuclear spin polarizations are encoded in the diamagnetic products. These manifest in the NMR spectrum as strongly enhanced absorptive or emissive signals. The integral intensities of these polarized signals reflect the hyperfine coupling constants in the reacting radicals, while the signs of the polarizations provide insight into the underlying reaction mechanism. In addition to mechanistic insights, CIDNP spectra may also offer access to some quantitative information, such as approximate radical content or relative reaction rates. In typical CIDNP experiment, background NMR signals are saturated prior to the light exposure of the sample. As a result, only polarizations that are generated by CIDNP effect appear in the spectrum.

### 2.7 ^1^H CIDNP in Acetonitrile

Ivocerin (**1**) is the *de facto* standard for Ge‐based photoinitiators and we find it highly informative to compare its photo‐chemical properties with those of our PEG‐substituted compounds **2 ‐ 8**.

**FIGURE 5:**
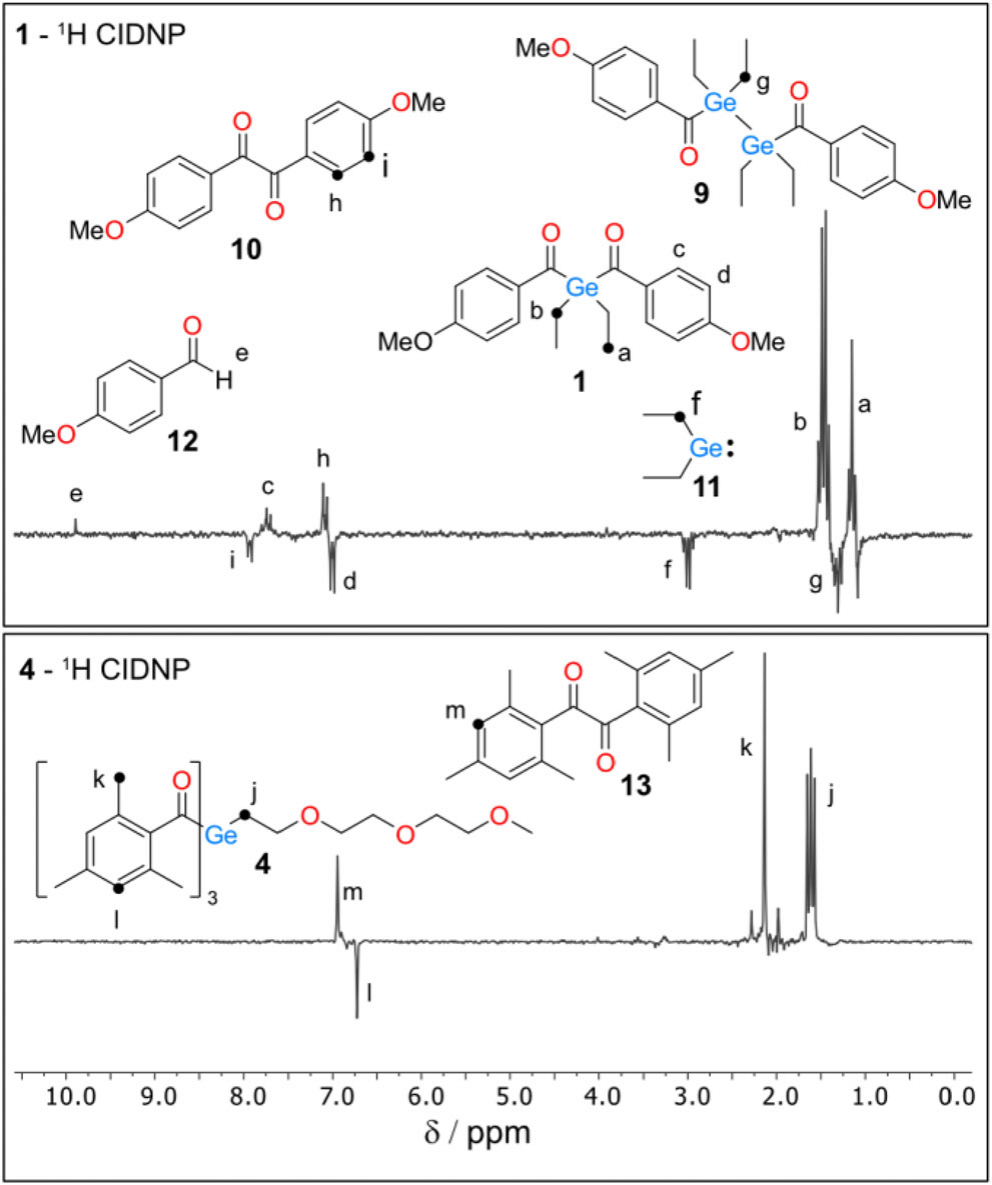
^1^H CIDNP spectra of **1** (top) and **4** (bottom) recorded 1 µs after the laser flash in Acetonitrile‐d_3_.

The comparison of ^1^H photo‐CIDNP spectra of **1** and **4** together with the signal assignments is shown in Figure 5. Both CIDNP spectra, recorded 1 µs after the laser (355 nm) flash, are dominated by the polarization of reactants. This is the result of the recombination (“cage”) reaction between two primary radicals — a germanium‐centered radical (**Ge**•) and a carbon‐centered radical (**C**•) — formed upon photoexcitation of compounds **1** and **4** (signals a—d, j—l in Figure 5). Alongside with the cage reactions, few escape processes manifest themselves in CIDNP spectra of **1** and **4**. When two identical germyl radicals stemming from **1** escape recombination reaction in the solvent cage, they can form diacyldigermane **9** (signal g, Figure 5). In a similar fashion, two p‐methoxybenzoyl radicals couple in the bulk to produce **10** (h, i, Figure 5) Moreover, germyl and *p*‐methoxybenzoyl radicals can undergo disproportionation reactions in cage, as well as in the bulk. Primary germyl radical cleaves producing germylene **11** (f, Figure 5), which is not persistent under the conditions of our experiments; methoxybenzoyl radical, in turn, can abstract hydrogen, presumably from the ethyl group of germyl radical, leading to aldehyde **12**.

**SCHEME 4:**
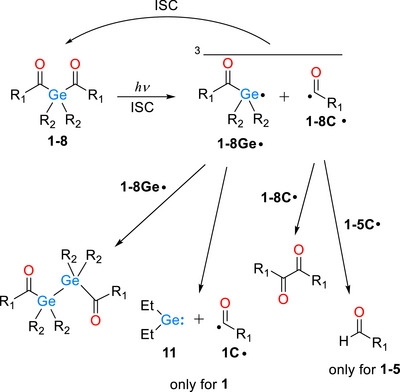
Photo‐induced reactions of **1 ‐ 8** as established by photo‐CIDNP.

With little alteration, PEG‐substituted acylgermanes **2** ‐ **8** exhibit similar reactivity patterns in ^1^H CIDNP spectra upon photolysis. Formation of germylene is not observed with any of **2 ‐ 8**, however, the traces of the aldehyde can be seen on a longer irradiation timescale (300 ms, UV lamp) with **2** ‐ **5**, where PEG chain is directly attached to the Ge‐radical center. Two distinct chromophores can be potentially cleaved away in photoinitiators **7** and **8**. Photo‐induced release of mesitoyl and *para*‐alkoxybenzoyl groups may lead to free radicals with different properties that can shape the properties of resulting polymer, still at this stage we do not see any evidence that this happens. All the processes that led to CIDNP polarizations observed with **1 ‐ 8** are shown in Scheme 4.

### 2.8 ^1^H CIDNP in the Presence of Monomer (Butyl Acrylate)

**FIGURE 6:**
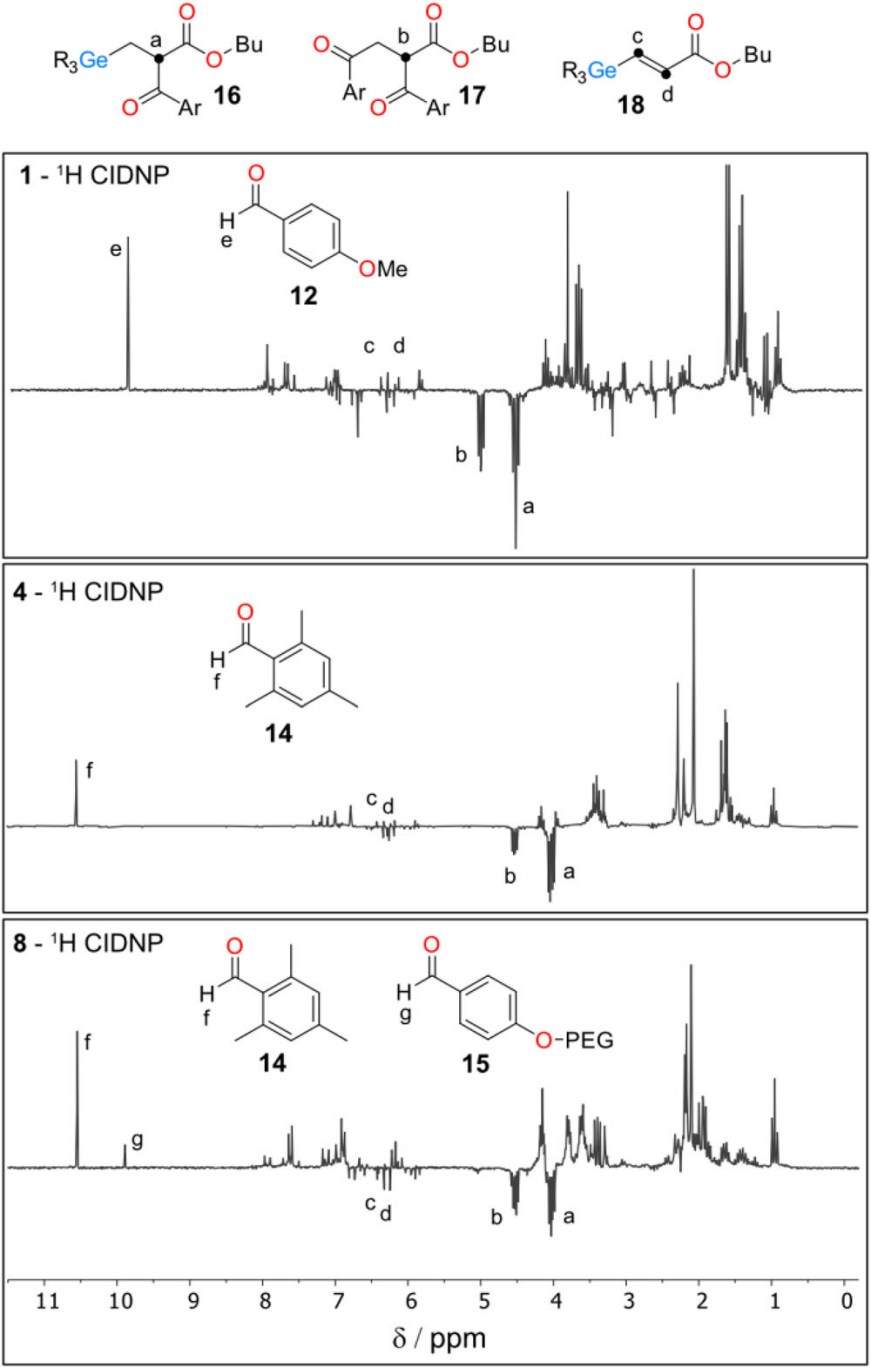
^1^H CIDNP spectra of **1**, **4**, and **8** in the presence of butyl acrylate as a quencher of free radicals, recorded using UV/Vis lamp (see Supporting Information) as the light source in Acetonitrile‐d_3_.

The reaction of free radicals constituting the primary radical pair with a quencher like monomer can lead to the formation of secondary radical pairs with distinct hyperfine profiles [42]. This can significantly alter the nuclear polarization patterns, providing additional information about both primary and secondary chemical processes. Indeed, the ^1^H CIDNP spectra of compounds **1**, **4**, and **8** in the presence of butyl acrylate (**M**), as shown in Figure 6, exhibit much greater complexity than those recorded without the monomer. In addition to the species depicted in Scheme 4, a number of new, distinct products of free radical reactions with **M** ‒ consistent across all three photoinitiators ‒ are observed in the spectra.

Under these moderately low monomer concentrations (compared to typical polymerization conditions), two major types of products can be identified in the CIDNP spectra (Figure 6). The first type includes products resulting from the initial addition to the double bond followed by termination with one of the primary free radicals (**16**, **17**). The second type consists of products formed via the initial addition of germyl radicals to the monomer, followed by hydrogen transfer to benzoyl‐type radicals (**12**, **14**, **15**, **18**).

**SCHEME 5:**
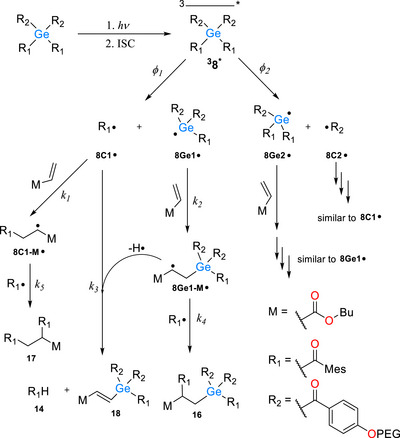
Reaction of free radicals formed upon photolysis of **8** as derived by ^1^H CIDNP in the presence of butyl acrylate (M).

The reactions leading to the CIDNP‐detected products in the presence of **M**, using compound **8** as an example, are shown in Scheme 5. Upon light exposure, compound **8** is promoted to its triplet excited state and undergoes cleavage. Two different carbon centered radicals can be released from **8** ‒ **8C1**• and **8C2**•. The competition between these two cleavage pathways is governed by the corresponding quantum yields *ϕ_1_* and *ϕ_2_*.

In addition to recombination and disproportionation reactions (Scheme 4), both radicals in a primary radical pair (**8Ge1**• and **8C1**•) can undergo addition to a double bond of monomer **M,** with respective rate constants *k_1_* and *k_2_*. Germyl radicals typically add to the double bond faster (*k_2_*) than benzoyl‐type radicals (*k_1_*) [17, 43, 44]. Thus, for each primary radical pair, there is a high probability that the Ge‐centered radical has already reacted with the monomer forming **8Ge1‐M**• while the C‐centered radical **8C1**• remains intact. The remaining benzoyl‐type radical **8C1**• can further react with **8Ge1‐M**• via hydrogen transfer (*k_3_*) or radical coupling (*k_4_*) leading to CIDNP‐observable products such as **14**, **16** and **18**. Alternatively, **8C1**• may diffuse away and add to another molecule of the monomer molecule (*k_1_*) forming **8C1‐M**•. This newly formed radical can then undergo quenching via radical‐radical termination with another **8C1**• species, governed by rate constant *k_5_*. Alternative radical pairs consisting of **8Ge1**• and **8C1**• exhibit similar reactivity, although their reaction rates may differ (Scheme 5).

Although these reactions do not represent all possible processes occurring in the system, we assume they are the most significant under our experimental conditions, as they are the ones reflected in the CIDNP spectra.

Kinetic equations that express the concentrations of the final products in terms of the rate constants of all involved processes can become quite complex in this case, and some reaction rates are not precisely established. However, kinetic simulations [45] with realistic parameters (see SI) show that, when all other parameters are held constant, the ratio of the concentrations of two different aldehydes ([R_1_H]/[R_2_H]) depends approximately linearly on the ratio of the quantum yields of the two alternative cleavage reactions (*ϕ_1_* / *ϕ_2_*). Since the CIDNP polarizations of aldehyde protons are proportional to the corresponding aldehyde concentrations they serve as a sensitive probe for monitoring variations in yields of these reactions. We consider the reaction pathways depicted in Scheme 5 to be sufficient for describing the influence of the competing α‐cleavage channels on the observed aldehyde ratio. The relative rates of these elementary steps fully account for the experimentally detected product distribution, and no additional parallel processes are required to rationalize the observations.

**FIGURE 7:**
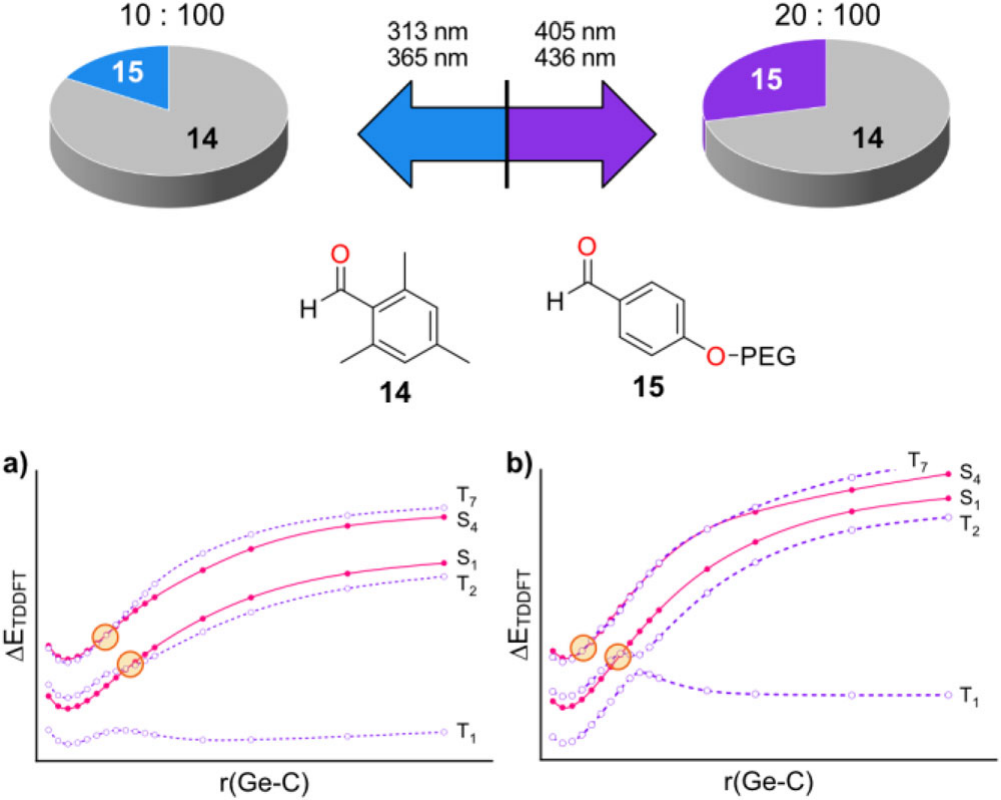
Top: Relative change of CIDNP polarizations produced by aldehydes **14** and **15** upon variation of the irradiation window. This change reflects relative alteration of quantum yields *ϕ_1_* and *ϕ_2_* in Scheme 5. Bottom: TDDFT dissociation curves of the singlet and triplet states of **8** showing important singlet‐triplet crossings (orange circles) and different barriers at the T_1_ and T_2_ states when dissociating the mesitoyl R_1_• (a) and the p‐alkoxybenzoyl R_2_• (b) moieties, respectively (the energy scales are kept identical to allow direct comparison).

By selecting different emission bands of the Hg/Xe high‐pressure UV lamp using optical filters, we were able to shift the [R1H]/[R2H] ratio from 100:10 under irradiation with the higher energy combination of wavelengths (313 nm and 365 nm) to 100:20 when using longer‐wavelength bands of the UV lamp at 405 nm and 436 nm (Figure 7). This observation suggests that irradiation at different wavelengths favors either the release of the mesitoyl radical R_1_• (**8C1**•) together with the corresponding germyl radical, or the formation of R_2_• (**8C2**•). Similar but a less quantitatively efficient control was achieved with the compound **7**. This provides an additional level of manipulation in photoinduced free radical polymerization: the ability to generate free radicals with distinct reactivities depending on the irradiation wavelength may enable the synthesis of polymers with tunable chemical and mechanical properties.

To rationalize experimental findings, we have performed TDDFT calculations on the release of either **R1•** (**8C1•)** or **R2•** (**8C2•**) from **8**. Fixed‐geometry excited‐state scans (Figure 7, bottom) for **8** demonstrate that both mesitoyl and p‐alkoxybenzoyl release originate from triplet excited states, with mesitoyl dissociation remaining dominant due to its lower adiabatic T_1_ barrier. Upon excitation at lower photon energies (longer wavelengths), however, the relative contribution of p‐alkoxybenzoyl release increases, which can be rationalized by pronounced differences in the excited‐state topology along the two dissociation coordinates. For p‐alkoxybenzoyl cleavage, the energy separation ΔE(T_2_‐T_1_) is substantially smaller than for mesitoyl cleavage, and the T_2_ surface exhibits a local minimum at approximately the Ge‐C distance where the T_1_ surface reaches its maximum, enabling efficient T_1_ ↔ T_2_ mixing and a two‐triplet‐state detour that alleviates the effective dynamical bottleneck for bond cleavage despite the higher adiabatic T_2_ barrier. In addition, the S_1_/T_2_ crossing occurs on the reactant side of the T_1_ maximum for p‐alkoxybenzoyl dissociation, whereas for mesitoyl dissociation it appears only at or beyond the T_1_ maximum, rendering early population transfer into the advantageous T_2_ pathway more effective for the former. Consistent with this topology, p‐alkoxybenzoyl release yields a more planar Ge‐centered radical than mesitoyl release, indicative of enhanced electronic stabilization and increased product commitment. Furthermore, an additional nonadiabatic doorway unique to the p‐alkoxybenzoyl coordinate is identified: a crossing between S_4_ and T_7_ occurs close to the equilibrium Ge–C bond length and is already accessible at low excitation energies, providing early population transfer into the triplet manifold while preserving p‐alkoxybenzoyl‐centered character. Collectively, the preferential population of p‐alkoxybenzoyl‐centered states at lower excitation energies, combined with early singlet–triplet injection and a favorable low‐lying triplet‐state topology, accounts for the experimentally observed increase in the relative contribution of p‐alkoxybenzoyl release at longer wavelengths, while mesitoyl cleavage remains overall dominant due to its intrinsically lower T_1_ barrier. Similar wavelength‐dependent branching has been reported for triplet‐mediated bond‐cleavage reactions, where nonadiabatic excited‐state topology rather than adiabatic barrier heights governs product selectivity [46, 47, 48].

### 2.9 Steady‐State LED Irradiation NMR

The wavelength‐dependent quantum yields observed for compounds **7** and **8** are not only consistent with the results of quantum mechanical calculations but are also fully supported by the steady‐state irradiation NMR experiments (irradiation by the LED photoreactor) in the presence of an excess of benzyl alcohol. In these experiments, yields of aldehydes R_1_H and R_2_H, formed via hydrogen transfer from PhCH_2_OH to radicals **8C1**• and **8C2**•, varied with the irradiation wavelength (365 and 405 nm see Supporting Information). The ratio of the two characteristic aldehydes was strongly dependent on the irradiation wavelength, indicating wavelength‐dependent changes in the relative rates of formation of the **8C1**• and **8C2**• radicals.

**SCHEME 6:**
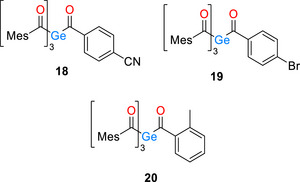
Mixed tetraacylgermanes reinvestigated.

We aimed to generalize our previously established paradigm by applying it to a slightly expanded set of chemical compounds, including systems presented in our earlier work (see Scheme 6) [25].

Whereas the *p*‐CN‐ and *p*‐Br‐substituted tetraacylgermanes **18** and **19** showed no evidence of competing cleavage, the *o*‐methyl derivative **20** not only exhibited such cleavage, but also displayed a stronger wavelength dependence than compounds **7** and **8**. Irradiation of **20** at 365 nm resulted exclusively in the formation of mesitoyl aldehyde, whereas irradiation at 450 nm corresponding to the long‐wavelength edge of the absorption spectrum of **20** yielded both mesitoyl and *o*‐methylbenzoyl aldehydes in comparable amounts (see Supporting Information). The increased yield of *o*‐methylbenzoyl aldehyde is consistent with the behavior reported by Barner‐Kowolik and co‐workers for oxime ester photoinitiators [49, 50]. Furthermore, these results indicate that wavelength‐selective fragmentation is a generally applicable pathway for carefully designed mixed functionalization

## 3 Conclusion

We have designed and synthesized a series of novel Ge‐based photoinitiators containing polyethylene glycol (PEG) moieties, that enable their application in polar environments. Photo‐CIDNP spectroscopy demonstrates that these compounds efficiently generate germanium‐centered radicals that readily add to vinyl monomers, exhibiting reactivity comparable to the commercial benchmark Ivocerin.

Beyond establishing their general photoinitiating performance, we show that mixed‐functionalized tetraacylgermanes containing mesitoyl and *para*‐alkoxybenzoyl units undergo competing α‐cleavage pathways, the relative contributions of which can be deliberately modulated by the irradiation wavelength.

We also demonstrate that photo‐CIDNP in combination with a suitable radical quencher (e.g., monomer), enables the quantification of competing photochemical pathways. These findings are independently confirmed by steady‐state LED irradiation NMR experiments and are fully consistent with TD‐DFT predictions of chromophore‐resolved excitation and dissociation curves.

Importantly, reinvestigation of structurally related mixed tetraacylgermanes demonstrates that wavelength‐selective fragmentation is a general phenomenon that emerges from carefully designed chromophore combinations, rather than an isolated case.

This wavelength‐dependent cleavage behavior provides an additional handle for controlling the nature of the initiating radical species. Such control over radical generation has the potential to fine‐tune polymer structure, composition, and material properties—particularly in applications requiring spatial or temporal precision, such as 3D printing, microfabrication, or multi‐stage curing systems.

Although the exact technological applications that will fully exploit these wavelength‐selective PIs are still emerging, the unique reactivity and tunability demonstrated here are likely to find use in next‐generation photopolymerization technologies as the field advances in the future.

## Conflicts of Interest

The authors declare no conflicts of interest.

## Supporting information



The authors have cited additional references within the Supporting Information [51, 52, 53, 54, 55, 56, 57, 58, 59, 60, 61, 62, 63, 64, 65, 66].
**Supporting File 1**: anie72089‐sup‐0001‐SuppMat.pdf.

**Supporting File 2**: anie72089‐sup‐0002‐Data.zip.

## Data Availability

The data that support the findings of this study are available in the supplementary material of this article. In addition, all underlying NMR, IR, UV/Vis, CIDNP and the relevant natural transition orbitals data supporting this work are openly available via the TU Graz repository at https://repository.tugraz.at/records/dy8vs‐7fd27.
