# Supplementary material for: Mixed‐Functionalized Acylgermanes Result in Wavelength‐Controlled Fragmentation
Source: Angew Chem Int Ed Engl. 2026 Apr 3;65(20):e5559644. doi: 10.1002/anie.5559644 (PMC13159412; doi:10.1002/anie.5559644)
Supplement: Supplementary file 1 — The authors have cited additional references within the Supporting Information [51, 52, 53, 54, 55, 56, 57, 58, 59, 60, 61, 62, 63, 64, 65, 66]. Supporting File 1: anie72089‐sup‐0001‐SuppMat.pdf. [file ANIE-65-e5559644-s001.pdf]

Supporting Information

Mixed-Functionalized Acylgermanes Result in Wavelength-controlled Fragmentation

André Culum,[a] Manfred Drusgala,[a] Roland C. Fischer,[a] Anne-Marie Kelterer,[b] Mario Leypold,[a] Dmytro Neshchadin*[b] and Michael Haas*[a]

[a] André Culum, MSc, Dr. Manfred Drusgala, Prof. Roland C. Fischer and Prof. Michael Haas

Institute of Inorganic Chemistry, Graz University of Technology

Stremayrgasse 9/IV, 8010 Graz (Austria)

E-mail: michael.haas@tugraz.at

[b] Dr. Dmytro Neshchadin, Prof. Anne-Marie Kelterer

Institute of Physical and Theoretical Chemistry, Graz University of Technology

E-mail: neshchadin@tugraz.at

Table of Contents

Additional synthetic information **3**

Synthesis of acid fluoride 6c 3

Attempted synthesis of polyethylene glycol substituted tetra-acylgermanes via multiple silyl abstraction 3

Experimental section **4**

Bis(2,4,6-trimethylbenzoyl)-bis(2-(2-(2-(2-methoxyethoxy)ethoxy)ethyl))germane (2) 4

Bis(2,4,6-trimethylbenzoyl)-bis(2-(2-(2-(2-methoxyethoxy)ethoxy)ethoxy)ethyl)germane (3) 4

Tris(2,4,6-trimethylbenzoyl)-(2-(2-(2-(2-methoxyethoxy)ethoxy)ethyl))germane (4) 4

Tris(2,4,6-trimethylbenzoyl)-(2-(2-(2-(2-(2-methoxyethoxy)ethoxy)ethoxy)ethyl))germane (5) 5

Methyl 4-(2-(2-(2-(2-methoxyethoxy)ethoxy)ethoxy)ethyl) benzoate (6a) 5

4-(2-(2-(2-(2-Methoxyethoxy)ethoxy)ethoxy)ethyl)benzoic acid (6b) 5

4-(2-(2-(2-(2-Methoxyethoxy)ethoxy)ethoxy)ethyl)benzoyl fluoride (6c) 6

Tetra(4-(2-(2-(2-(2-methoxyethoxy)ethoxy)ethoxy)ethyl)benzoyl)germane (6d) 6

4-(2-(2-(2-(2-Methoxyethoxy)ethoxy)ethoxy)ethyl)benzoyl-tris(2,4,6-trimethylbenzoyl)germane (7) 6

Bis(4-(2-(2-(2-(2-Methoxyethoxy)ethoxy)ethoxy)ethyl)benzoyl)-bis(2,4,6-trimethylbenzoyl)germane (8) 7

NMR Spectroscopy **8**

Bis(2,4,6-trimethylbenzoyl)-bis(2-(2-(2-(2-methoxyethoxy)ethoxy)ethyl))germane (2) 8

Bis(2,4,6-trimethylbenzoyl)-bis(2-(2-(2-(2-methoxyethoxy)ethoxy)ethoxy)ethyl)germane (3) 9

Tris(2,4,6-trimethylbenzoyl)-(2-(2-(2-(2-methoxyethoxy)ethoxy)ethyl))germane (4) 10

Tris(2,4,6-trimethylbenzoyl)-(2-(2-(2-(2-(2-methoxyethoxy)ethoxy)ethoxy)ethyl))germane (5) 11

Methyl 4-(2-(2-(2-(2-methoxyethoxy)ethoxy)ethoxy)ethyl) benzoate (6a) 12

4-(2-(2-(2-(2-Methoxyethoxy)ethoxy)ethoxy)ethyl)benzoic acid (6b) 13

4-(2-(2-(2-(2-Methoxyethoxy)ethoxy)ethoxy)ethyl)benzoyl fluoride (6c) 14

4-(2-(2-(2-(2-Methoxyethoxy)ethoxy)ethoxy)ethyl)benzoyl-tris(2,4,6-trimethylbenzoyl)germane (7) 15

Bis(4-(2-(2-(2-(2-Methoxyethoxy)ethoxy)ethoxy)ethyl)benzoyl)-bis(2,4,6-trimethylbenzoyl)germane (8) 16

Tetra(4-(2-(2-(2-(2-methoxyethoxy)ethoxy)ethoxy)ethyl)benzoyl)germane (6d) 17

UV-Vis Spectroscopy **18**

CIDNP Experiments **21**

Density Functional Theory Calculations **39**

Computational Methods 39

Computational results 39

Single Crystal X-ray Crystallography **54**

Additional synthetic information

Synthesis of acid fluoride 6c

Given the superior spectroscopic properties of tetraacylgermanes in comparison to di- and tri-acylgermanes, we developed a synthetic strategy towards these molecules. It was deemed necessary to prepare the respective polyethylene glycol substituted aromatic acid fluorides. The starting point was a simple SN_2 substitution resulting in the formation of the desired polyethylene glycol substituted aromatic ester **6a** in good yields. Subsequent hydrolysis gives rise to the corresponding acid **6b** in nearly quantitative yield. The acid fluoride **6c** was obtained by the conversion with diethylaminosulfur trifluoride (DAST) in in nearly quantitative yield (see Scheme S1).

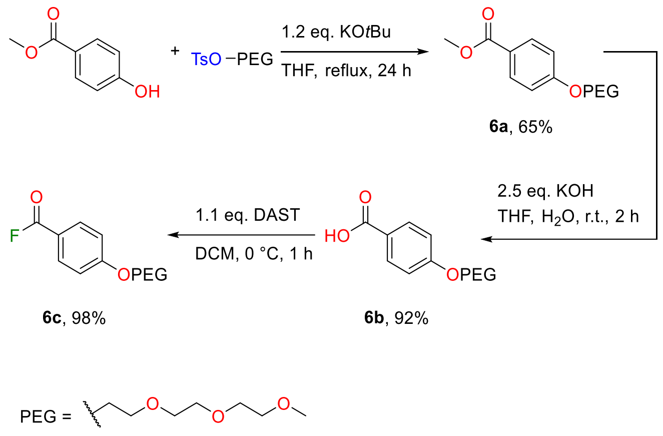

Scheme S1. Preparation of the acid fluoride **6c**.

Attempted synthesis of polyethylene glycol substituted tetra-acylgermanes via multiple silyl abstraction

Following our initial successes of preparing tetraacylgermanes from the respective bisenolate and triacylgermenolate, we wanted to prepare polyethylene substituted tetraacylgermane **6d**. Consequently, we used tetrakis(trimethylsilyl)germane as a starting material and reacted this compound with 1.05 eq. of potassium *tert*-butoxide to generate the germanide via a targeted silyl abstraction reaction. This reaction mixture was subsequently reacted with 4.0 eq. of acid fluoride **6c** yielding target compound **6d**. To our dismay, flash column chromatography, preparative TLC, as well as the use of a preparative LPLC have proven ineffective in purifying the crude reaction mixture. However, crude NMR spectra could be obtained, proving the formation of the desired product. The lack of a sufficiently pure product means that no further characterization nor photochemical experiments of compound **6d** were performed.

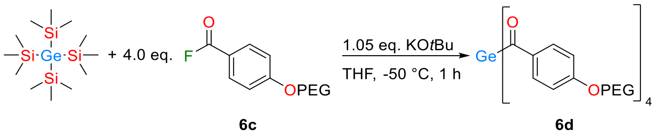

Scheme S2. Synthesis of polyethylene glycol substituted tetraacylgermanes.

Experimental section

All experiments were performed under a nitrogen atmosphere using standard Schlenk techniques. Solvents were dried using a column solvent purification system.[51] KO*t*Bu (>98%), 1-(2-Bromoethoxy)-2-(2-methoxyethoxy)ethane (95%), 1-Brom-2-(2-methoxyethoxy)ethane (95%), C_6D_6 (99.5 atom%, D) and $CDCl_3$ (99.8 atom% D) were used without any further purification. tetrakis(trimethylsilyl)germane and FCO(Mes) were prepared according to published procedures.[52]

^{1}H- and ^{13}C-NMR spectra were recorded on a Varian INOVA 400, Bruker Ultrashield 300 or a 400 MHz JEOL JNM ECZL spectrometer with a Royal HFX-Probes with an autosampler. Samples were prepared in C_6D_6 or $CDCl_3$ solutions and referenced versus TMS using the internal ^{2}H-lock signal of the solvent.

Infrared spectra were obtained on a Bruker Alpha-P Diamond ATR Spectrometer from the solid samples. Melting points were determined using Stuart SMP50 apparatus and are uncorrected. Elemental analyses were carried out on a Hanau Vario Elementar EL apparatus. UV absorption spectra were recorded on a Perkin Elmer Lambda 5 spectrometer.

Bis(2,4,6-trimethylbenzoyl)-bis(2-(2-(2-(2-methoxyethoxy)ethoxy)ethyl))germane (2)

To a cooled (-50 °C) solution of 704 mg 11-bromo-2,5,8-trioxaundecane (3.10 mmol; 2.05 eq.) in 5 ml of THF, 1.0 g (1.51 mmol; 1.00 eq.) of bisenolate $K_2Ge[(CO)Mes]_2$ was added. The reaction mixture was brought to room temperature and stirred overnight. A turbid dark brown reaction mixture was obtained. TLC analysis ($CHCl_3$/EtOAc, 1/5, v/v) confirmed complete conversion of the starting material. The reaction mixture was diluted with 50 ml diethyl ether and washed with a saturated NH_4Cl solution, the organic layer was dried with Na_2SO_4 and the solvent removed in vacuum. The crude reaction mixture was purified via column chromatography ($CHCl_3$/EtOAc, 1/8, v/v). Yield: 320 mg (0.48 mmol; 32%) of analytically pure **2** as a yellow oil. Anal. Calc. (%) for $C_{34}H_{52}GeO_8$: C, 61.74; H, 7.92, Found: C, 61.92; H, 7.99. ^{13}C-NMR Data (75.5 MHz, C_6D_6, ppm): 241.29 (Mes-*C*=O), 143.64 (Mes-*C4*), 138.58 (Mes-*C1*), 132.52 (Mes-*C2*), 129.15 (Mes-*C3*), 72.35, 70.84, 70.78, 70.06, 67.76 (PEG-*C*H$_2$), 58.69 (PEG-*C*H$_3$), 21.05, 19.23 (Mes-*C*H$_3$), 17.61 (Ge-*C*H$_2$). ^{1}H-NMR Data (300 MHz, C_6D_6, ppm): 6.67 (s, 4H, Mes-*H*), 3.50–3.30 (m, 20H, PEG-C*H$_2$*), 3.26 (s, 6H, PEG-C*H$_3$*), 2.15 (s, 6H, Mes-C*H$_3$*), 2.00 (s, 12H, Mes-C*H$_3$*), 1.43 (t, 2H, J = 7.8 Hz, Ge-C*H$_2$*). UV-vis: λ [nm] (ε [L mol^{-1} cm^{-1}]) = 354 (401), 370 (575), 386 (735), 406 (747). IR (neat): ν(C=O) = 1630, 1600.

Bis(2,4,6-trimethylbenzoyl)-bis(2-(2-(2-(2-methoxyethoxy)ethoxy)ethoxy)ethyl)germane (3)

To a cooled (-50 °C) solution of 839 mg 13-bromo-2,5,8,11-tetraoxatridecane (3.10 mmol; 2.05 eq.) in 5 ml of THF, 1.0 g (1.51 mmol; 1.00 eq.) of bisenolate $K_2Ge[(CO)Mes]_2$ was added. The reaction mixture was brought to room temperature and stirred overnight. A turbid dark brown reaction mixture was obtained. TLC analysis ($CHCl_3$/EtOAc, 1/5, v/v) confirmed complete conversion of the starting material. The reaction mixture was diluted with 50 ml diethyl ether and washed with a saturated NH_4Cl solution, the organic layer was dried with Na_2SO_4 and the solvent removed in vacuum. The crude reaction mixture was purified via column chromatography ($CHCl_3$/EtOAc, 1/8, v/v). Yield: 622 mg (0.83 mmol; 55%) of analytically pure **3** as a yellow oil. Anal. Calc. (%) for $C_{38}H_{60}GeO_{10}$: C, 60.89; H, 8.07, Found: C, 60.26; H, 7.64. ^{13}C-NMR Data (75.5 MHz, $CDCl_3$, ppm): 241.50 (Mes-*C*=O), 142.68 (Mes-*C4*), 138.93 (Mes-*C1*), 132.03 (Mes-*C2*), 128.92 (Mes-*C3*), 72.00, 70.65, 70.59, 70.58, 70.50, 69.58, 67.39 (PEG-*C*H$_2$), 59.07 (PEG-*C*H$_3$), 21.13, 18.98 (Mes-*C*H$_3$), 16.87 (Ge-*C*H$_2$). ^{1}H-NMR Data (300 MHz, $CDCl_3$, ppm): 6.66 (s, 4H, Mes-*H*), 3.73–3.27 (m, 28H, PEG-C*H$_2$*), 3.26 (s, 6H, PEG-C*H$_3$*), 2.15 (s, 6H, Mes-C*H$_3$*), 1.99 (s, 12H, Mes-C*H$_3$*), 1.52 (t, 2H, J = 7.8 Hz, Ge-C*H$_2$*). UV-vis: λ [nm] (ε [L mol^{-1} cm^{-1}]) = 356 (574), 369 (858), 385 (1108), 405 (1076). IR (neat): ν(C=O) = 1620, 1585.

Tris(2,4,6-trimethylbenzoyl)-(2-(2-(2-(2-methoxyethoxy)ethoxy)ethyl))germane (4)

To a cooled (-50 °C) solution of 345 mg 11-bromo-2,5,8-trioxaundecane (1.52 mmol; 1.05 eq.) in 5 ml of THF, a solution of 1.0 g (1.45 mmol; 1.00 eq.) of potassium trimesitylgermenolate $KGe[(CO)Mes]_3$ in 10 ml THF was added dropwise over the course of 5 minutes. The reaction mixture was brought to room temperature and stirred for 60 minutes. An opaque yellow reaction mixture was obtained. TLC analysis (toluene/EtOAc, 2/1, v/v) confirmed complete conversion of the starting material. The reaction mixture was diluted with 50 ml diethyl ether and washed with a saturated NH_4Cl solution, the organic layer was dried

with Na_2SO_4 and the solvent removed in vacuum. The crude reaction mixture was purified via column chromatography (toluene/EtOAc, 5/1, v/v). Yield: 460 mg (0.70 mmol; 48%) of analytically pure **4** as a yellow oil. Anal. Calc. (%) for $C_{37}H_{48}GeO_6$: C, 67.19; H, 7.32, Found: C, 67.53; H, 7.11. ^{13}C-NMR Data (75.5 MHz, $CDCl_3$, ppm): 236.03 (Mes-*C*=O), 141.82 (Mes-*C4*), 139.21 (Mes-*C1*), 132.45 (Mes-*C2*), 128.71 (Mes-*C3*), 71.82, 70.40, 70.34, 69.28, 66.84 (PEG-CH_2), 58.91 (PEG-CH_3), 21.04, 18.96 (Mes-CH_3), 18.28 (Ge-CH_2). ^{1}H-NMR Data (300 MHz, $CDCl_3$, ppm): 6.66 (s, 6H, Mes-*H*), 3.59–3.37 (m, 10H, PEG-CH_2), 3.34 (s, 3H, PEG-CH_3), 2.22 (s, 9H, Mes-CH_3), 2.05 (s, 18H, Mes-CH_3), 1.70 (t, 2H, *J* = 8.2 Hz, Ge-CH_2). UV-vis: λ [nm] (ε [L mol^{-1} cm^{-1}]) = 356 (641), 371 (998), 382 (1242), 401 (893). IR (neat): ν(C=O) = 1630, 1605.

Tris(2,4,6-trimethylbenzoyl)-(2-(2-(2-(2-(2-methoxyethoxy)ethoxy)ethoxy)ethyl))germane (5)

To a cooled (-50 °C) solution of 413 mg 13-bromo-2,5,8,11-tetraoxatridecane (1.52 mmol; 1.05 eq.) in 5 ml of THF, a solution of 1.0 g (1.45 mmol; 1.00 eq.) of potassium trimesitylgermenolate KGe[(CO)Mes]$_3$ in 10 ml of THF was added dropwise over the course of 5 minutes. The reaction mixture was brought to room temperature and stirred for 60 minutes. An opaque yellow reaction mixture was obtained. TLC analysis (toluene/EtOAc, 2/1, v/v) confirmed complete conversion of the starting material. The reaction mixture was diluted with 50 ml diethyl ether and washed with a saturated NH_4Cl solution, the organic layer was dried with Na_2SO_4 and the solvent removed in vacuum. The crude reaction mixture was purified via column chromatography (toluene/EtOAc, 5/1, v/v). Yield: 522 mg (0.91 mmol; 51%) of analytically pure **5** as a yellow oil. Anal. Calc. (%) for $C_{39}H_{52}GeO_7$: C, 66.40; H, 7.43, Found: C, 66.79; H, 7.32. ^{13}C-NMR Data (101 MHz, $CDCl_3$, ppm): 236.33 (Mes-*C*=O), 142.02 (Mes-*C4*), 139.48 (Mes-C1), 132.71 (Mes-*C2*), 128.95 (Mes-*C3*), 72.10, 70.73, 70.71, 70.69, 70.56, 69.53, 67.09 (PEG-CH_2), 59.21 (PEG-CH_3), 21.30, 19.22 (Mes-CH_3), 18.47 (Ge-CH_2). ^{1}H-NMR Data (400 MHz, $CDCl_3$, ppm): 6.67 (s, 6H, Mes-*H*), 3.63–3.58 (m, 6H, PEG-CH_2), 3.55–3.50 (m, 4H, PEG-CH_2), 3.44–3.40 (m, 4H, PEG-CH_2), 3.38–3.35 (m, 5H, PEG-CH_2, PEG-CH_3), 2.24 (s, 9H, Mes-CH_3), 2.05 (s, 18H, Mes-CH_3), 1.710–1.67 (m, 2H, Ge-CH_2). UV-vis: λ [nm] (ε [L mol^{-1} cm^{-1}]) = 356 (1268), 371 (1970), 383 (2396), 402 (1709). IR (neat): ν(C=O) = 1650, 1615.

Methyl 4-(2-(2-(2-(2-methoxyethoxy)ethoxy)ethoxy)ethyl) benzoate (6a)

A 150 mL THF solution of 7.72 g methyl 2-(4-hydroxybenzoyl)benzoate (51.0 mmol; 1.0 eq.), 2.10 g 2-(2-(2-methoxyethoxy)ethoxy)ethyl 4-methylbenzenesulfonate (66.3 mmol; 1.3 eq.), 6.83 g potassium *t*-butoxide (61.2 mmol; 1.2 eq.) and 0.85 g potassium iodide (5.1 mmol; 0.1 eq.) was refluxed for 24h. After complete conversion of starting materials was observed by TLC (cyclohexane/EtOAc, 1/1, v/v), the, dark brown, reaction mixture was concentrated under reduced pressure, diluted with 200 mL of ethyl acetate and washed with an aqueous NH_4Cl solution. The organic layer was dried with $NaSO_4$ and concentrated *in* vacuum. The crude reaction mixture was purified via column chromatography (cyclohexane/EtOAc, 3/1, v/v). Yield: 9.42 g (31.6 mmol; 65%).^{13}C-NMR Data (75.5 MHz, $CDCl_3$, ppm): 166.83 (Aryl-*C*=O), 162.61 (Aryl-*C4*), 131.57 (Aryl-*C2*), 122.77 (Aryl-*C1*), 114.24 (Aryl-*C3*), 71.97, 70.92, 70.69, 70.60, 69.59, 67.62 (PEG-CH_2), 59.03 (PEG-CH_3), 51.85 (ester-CH_3). ^{1}H-NMR Data (101 MHz, $CDCl_3$, ppm): 7.95 (d, 2H, *J* = 8.9 Hz, Aryl-*H*), 6.91 (d, 2H, *J* = 8.7 Hz, Aryl-*H*), 4.14 (t, 2H, *J* = 4.8 Hz, PEG-CH_2), 3.85–3.82 (m, 5H, ester-CH_3, PEG-CH_2), 3.72–3.49 (m, 8H, PEG-CH_2), 3.34 (s, 3H, PEG-CH_3).

4-(2-(2-(2-(2-Methoxyethoxy)ethoxy)ethoxy)ethyl)benzoic acid (6b)

A 60 mL THF solution of 9.42 g **6a** (31.6 mmol; 1.0 eq.) was mixed with 40 mL of an aqueous solution of potassium hydroxide 4.40 g (79 mmol; 2.5 eq.). Complete conversion of starting materials was observed after 2 h *via* TLC (cyclohexane/EtOAc, 1/1, v/v). Subsequently, the reaction was acidified to a pH of ~3 by the addition of a saturated $KHSO_4$ solution and the THF was removed *in vacuo*, yielding an opaque suspension of the product in water. The obtained suspension was kept at 0 °C for 30 min allowing a large amount of white crystalline precipitate to form. The product was then filtered off and washed with cold distilled water and dried, yielding the analytically pure product as glistening white crystals. Yield: 8.30 g (29.2 mmol; 92%). Mp: 78-79°C.^{13}C-NMR Data (101 MHz, $CDCl_3$, ppm): 171.58 (Aryl-*C*=O), 163.34 (Aryl-*C4*), 132.39 (Aryl-*C2*), 121.99 (Aryl-*C1*), 114.42 (Aryl-*C3*), 72.03, 71.00, 70.77, 70.68, 69.64, 67.73 (PEG-CH_2), 59.15 (PEG-CH_3).^{1}H-NMR Data (400 MHz, $CDCl_3$, ppm): 8.04 (d, 2H, *J* = 9.0 Hz, Aryl-*H*), 6.95 (d, 2H, *J* = 9.0 Hz, Aryl-*H*), 4.19 (t, 2H, *J* = 4.8 Hz, PEG-CH_2), 3.88 (t, 2H, *J* = 4.8 Hz, PEG-CH_2), 3.76–3.50 (m, 8H, PEG-CH_2), 3.38 (s, 3H, PEG-CH_3).

4-(2-(2-(2-(2-Methoxyethoxy)ethoxy)ethoxy)ethyl)benzoyl fluoride (6c)

6.0 g of the carboxylic acid **6b** (21.2 mmol; 1 eq.) were dissolved in 100 mL of DCM and cooled to 0 °C. To this solution 3.07 mL of diethylaminosulfur trifluoride (23.3 mmol; 1.1 eq) were added dropwise. After 1 h of stirring at 0 °C the reaction was diluted with 150 mL of DCM and washed with 300 mL of distilled water, the organic layer was dried with $MgSO_4$ and concentrated under reduced pressure. The obtained product, a clear pale-yellow oil was used for subsequent reaction steps without further purification. Yield: 6.0 g (20.9 mmol; 98%).^{13}C-NMR Data (75.5 MHz, $CDCl_3$, ppm): 164.48 (Aryl-*C4*), 159.47, 154.97 (d, *J* (C-F) = 339.8 Hz, Aryl-*C*=O), 133.72, 133.66 (d, *J* (C-F) = 4.1 Hz, Aryl-*C2*), 117.39, 116.57 (d, *J* (C-F) = 62.0 Hz, Aryl-*C1*), 115.00 (Aryl-*C3*), 71.93, 70.92, 70.66, 70.59, 69.41, 67.89 (PEG-CH_2), 59.02 (PEG-CH_3).^{1}H-NMR Data (300 MHz, $CDCl_3$, ppm): 7.90 (d, 2H, *J* = 8.8 Hz, Aryl-*H*), 6.93 (d, 2H, *J* = 8.2 Hz, Aryl-*H*), 4.15 (t, 2H, *J* = 4.5 Hz, PEG-CH_2), 3.82 (t, 2H, *J* = 4.8 Hz, PEG-CH_2), 3.70–3.44 (m, 8H, PEG-CH_2), 3.30 (s, 3H, PEG-CH_3).

Tetra(4-(2-(2-(2-(2-methoxyethoxy)ethoxy)ethoxy)ethyl)benzoyl)germane (6d)

To a solution of tetrakis(trimethylsilyl)germanium(IV) 1.0 g (2.7 mmol, 1 eq.) in DME, 322 mg of KO*t*Bu (2.8 mmol, 1.05 eq.) was added and the reaction mixture was stirred for 2 h at room temperature. 3.13 g of acid fluoride **6c** (10.8 mmol, 4.0 eq.) were added in quarter portions over the course of 1 h at 0 °C, allowing the reaction to come to room temperature between additions of the acid fluoride portions. The reaction was stirred overnight, TLC analysis ($CHCl_3$/MeOH, 15/1, v/v) revealed the formation of the expected product. The reaction was washed with a saturated NH_4Cl solution, the organic layer was dried with Na_2SO_4 and concentrated *in vacuo*. Purification of this compound was attempted via column chromatography in multiple attempts, neither of which were successful. Preparative thin layer chromatography ($CHCl_3$/MeOH, 20/1, v/v) has also proven inefficient in the purification of the target compound. The use of a Biotage preparative LPLC system was also unsuccessful in isolating the desired compound.

4-(2-(2-(2-(2-Methoxyethoxy)ethoxy)ethoxy)ethyl)benzoyl-tris(2,4,6-trimethylbenzoyl)germane (7)

To a cooled (-50 °C) solution of 530 mg **6c** (1.75 mmol; 1.20 eq.) in 5 ml of THF, a solution of 1.0 g (1.45 mmol; 1.00 eq.) of trimesitylgermenolate KGe[(CO)Mes]$_3$ in 10 ml THF was added dropwise over the course of 5 minutes. The reaction mixture was brought to room temperature and stirred for 60 minutes. A bright yellow reaction mixture was obtained. TLC analysis (toluene/EtOAc, 2/1, v/v) confirmed complete conversion of the starting material. The reaction mixture was diluted with 50 ml diethyl ether and washed with a saturated NH_4Cl solution, the organic layer was dried with Na_2SO_4 and the solvent removed in vacuum. The crude reaction mixture was purified via column chromatography (toluene/EtOAc, 5/1, v/v). Yield: 715 mg (0.91 mmol; 63%) of analytically pure **7** as a yellow oil. Single crystals could be obtained via crystallization from hot toluene. Mp: 63°C. Anal. Calc. (%) for $C_{44}H_{52}GeO_8$: C, 67.62; H, 6.71, Found: C, 67.73; H, 6.86. ^{13}C-NMR Data (101 MHz, $CDCl_3$, ppm): 233.21 (Mes-*C*=O), 217.54 (Aryl-*C*=O), 163.26 (Aryl-*C4*), 141.51 (Mes-*C4*), 139.66 (Mes-*C1*), 133.74 (Aryl-*C1*), 133.12 (Mes-*C2*), 131.75 (Aryl-*C2*), 128.77 (Mes-*C3*), 114.28 (Aryl-*C3*), 72.05, 71.02, 70.77, 70.71, 69.52, 67.79 (PEG-CH_2), 59.13 (PEG-CH_3), 21.21, 19.33 (Mes-CH_3). ^{1}H-NMR Data (400 MHz, $CDCl_3$, ppm): 7.78 (d, 2H, *J* = 8.6 Hz, Arom-*H*), 6.83 (d, 2H, *J* = 8.6 Hz, Arom-*H*), 6.65 (s, 6H, Mes-*H*), 4.20 (t, 2H, *J* = 4.8 Hz, PEG-CH_2), 3.93 (t, 2H, *J* = 4.8 Hz, PEG-CH_2), 3.84 – 3.57 (m, 8H, PEG-CH_2), 3.46 (s, 3H, PEG-CH_3), 2.27 (s, 9H, Mes-CH_3), 2.19 (s, 18H, Mes-CH_3). UV-vis: λ [nm] (ε [L mol^{-1} cm^{-1}]) = 368 (2390), 380 (2656), 397 (2194), 422 (1544). IR (neat): ν(C=O) = 1620, 1585.

Bis(4-(2-(2-(2-(2-Methoxyethoxy)ethoxy)ethoxy)ethyl)benzoyl)-bis(2,4,6-trimethylbenzoyl)germane (8)

To a cooled (-50 °C) solution of 939 mg **6c** (3.10 mmol; 2.05 eq.) in 5 ml of THF, 1.0 g (1.51 mmol; 1.00 eq.) of bisenolate $K_2Ge[(CO)Mes]_2$ was added. The reaction mixture was brought to room temperature and stirred overnight. A turbid dark brown reaction mixture was obtained. TLC analysis ($CHCl_3$/EtOAc, 1/5, v/v) confirmed complete conversion of the starting material. The reaction mixture was diluted with 50 ml diethyl ether and washed with a saturated NH_4Cl solution, the organic layer was dried with Na_2SO_4 and the solvent removed in vacuum. The crude reaction mixture was purified via column chromatography ($CHCl_3$/EtOAc, 1/8, v/v). Yield: 534 mg (0.59 mmol; 39%) of analytically pure **8** as a yellow oil. Anal. Calc. (%) for $C_{48}H_{60}GeO_{12}$: C, 63.94; H, 6.71, Found: C, 64.43; H, 7.00. ^{13}C-NMR Data (75.5 MHz, $CDCl_3$, ppm): 232.91 (Mes-*C*=O), 218.49 (Aryl-*C*=O), 163.43 (Aryl-*C4*), 141.51 (Mes-*C4*), 139.69 (Mes-*C1*), 133.97 (Aryl-*C1*), 133.00 (Mes-*C2*), 131.76 (Aryl-*C2*), 128.81 (Mes-*C3*), 114.55 (Aryl-*C3*), 72.03, 70.99, 70.74, 70.68, 69.50, 67.79 (PEG-*C*H_2), 59.11 (PEG-*C*H_3), 21.17, 19.28 (Mes-*C*H_3). ^{1}H-NMR Data (101 MHz, $CDCl_3$, ppm): 7.70 (d, 4H, J = 8.9 Hz, Aryl-*H*), 6.79 (d, 4H, J = 8.8 Hz, Aryl-*H*), 6.56 (s, 4H, Mes-*H*), 4.11 (t, 4H, J = 4.7 Hz, PEG-CH_2), 3.83 (t, 2H, J = 4.7 Hz, PEG-CH_2), 3.73–3.60 (m, 12H, PEG-CH_2), 3.58– 3.48 (m, 4H, PEG-CH_2), 3.35 (s, 6H, PEG-CH_3), 2.15 (s, 6H, Mes-CH_3), 2.12 (s, 12H, Mes-CH_3). UV-vis: λ [nm] (ε [L mol^{-1} cm^{-1}]) = 370 (1956), 383 (2240), 400 (1978), 425 (1419). IR (neat): ν(C=O) = 1635, 1590.

NMR Spectroscopy

Bis(2,4,6-trimethylbenzoyl)-bis(2-(2-(2-(2-methoxyethoxy)ethoxy)ethyl))germane (2)

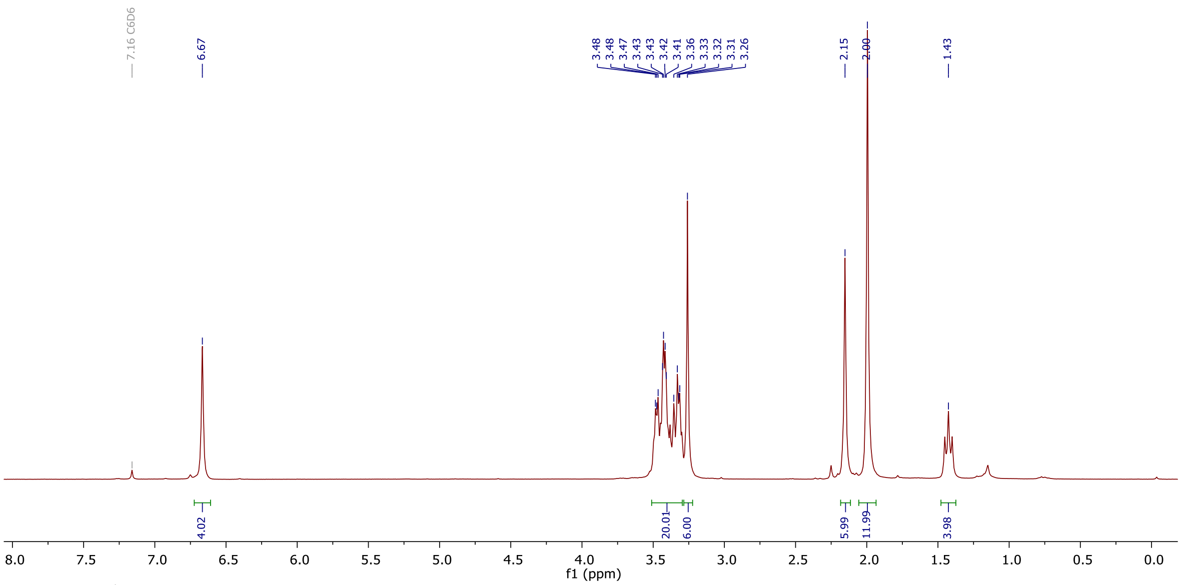

Figure S1. ^{1}H- spectrum of **2** (C_6D_6 solution, vs ext. TMS, ppm).

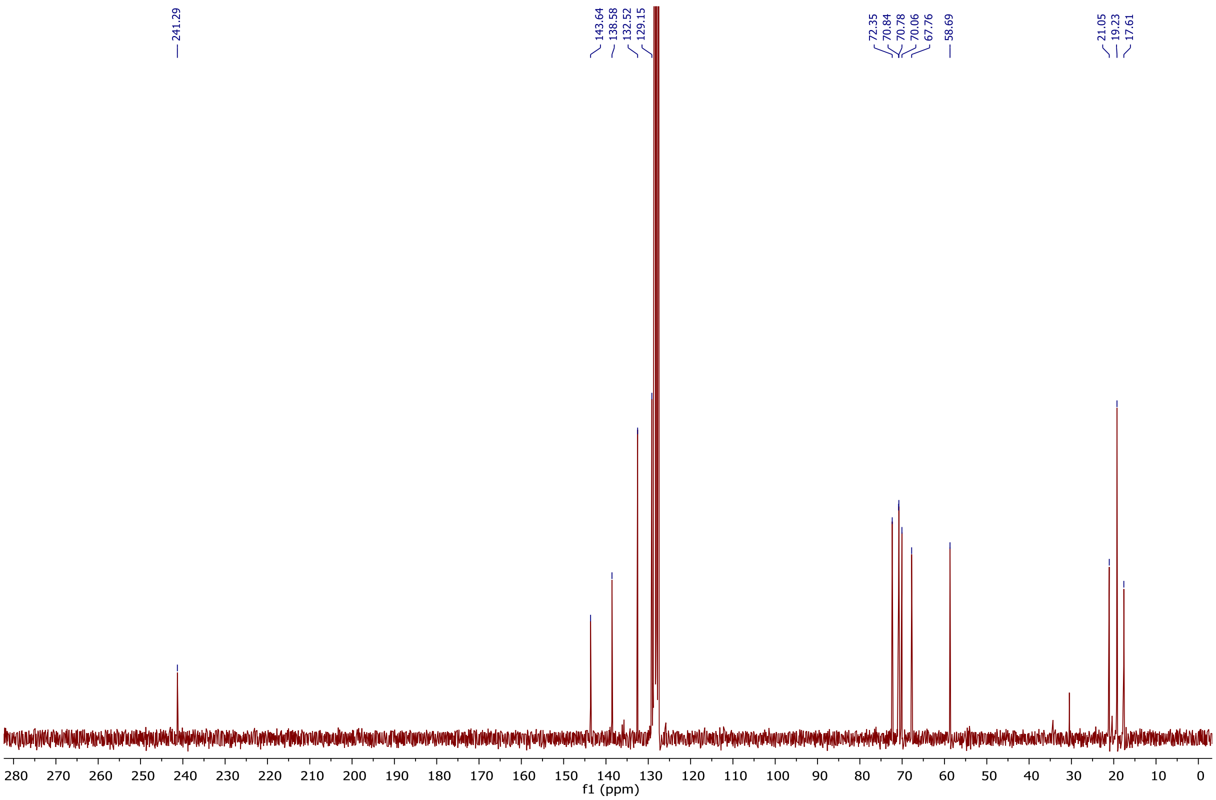

Figure S2. ^{13}C-NMR spectrum of **2** (C_6D_6 solution, vs ext. TMS, ppm).

Bis(2,4,6-trimethylbenzoyl)-bis(2-(2-(2-(2-methoxyethoxy)ethoxy)ethoxy)ethyl)germane (3)

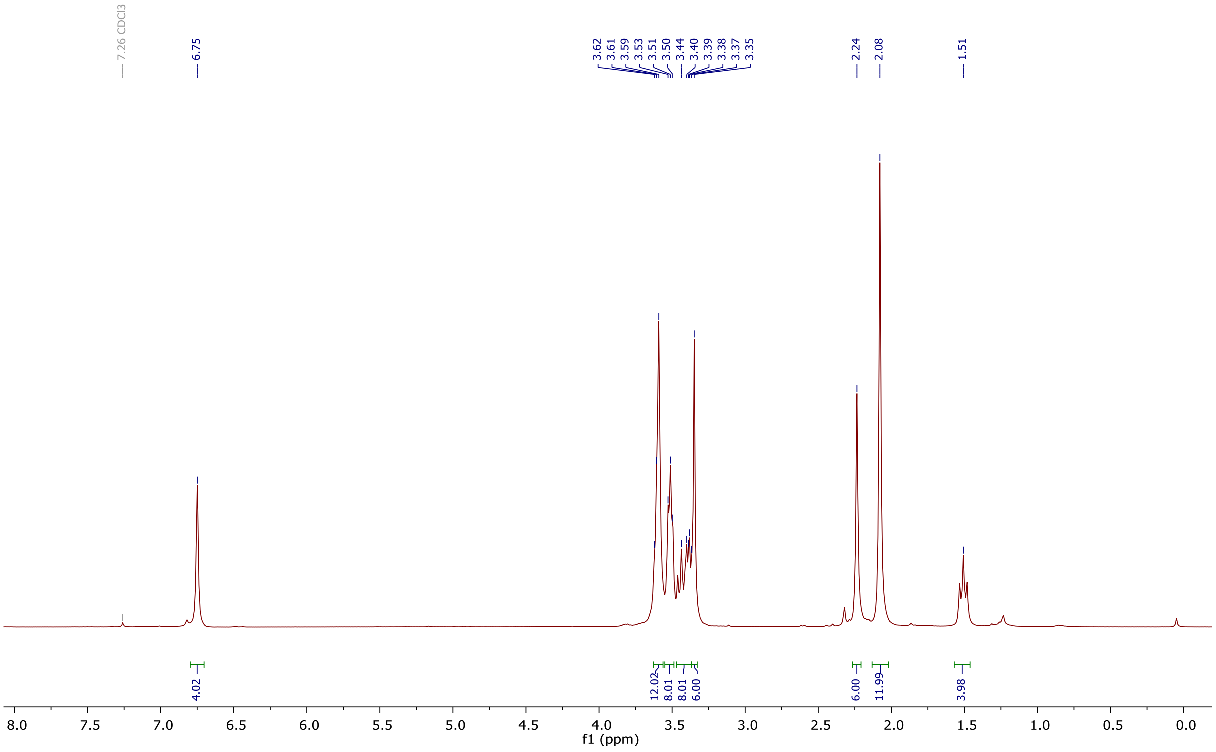

Figure S3. ^{1}H- spectrum of **3** ($CDCl_3$ solution, vs ext. TMS, ppm).

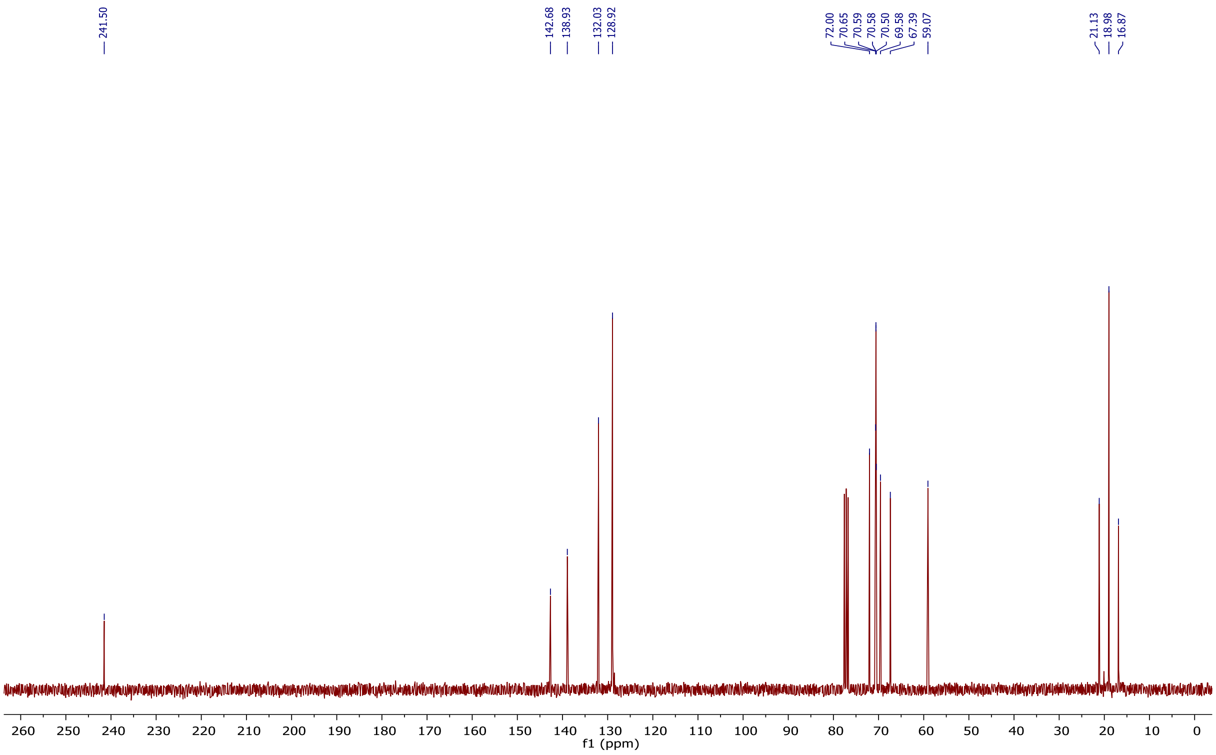

Figure S4. ^{13}C-NMR spectrum of **3** ($CDCl_3$ solution, vs ext. TMS, ppm).

Tris(2,4,6-trimethylbenzoyl)-(2-(2-(2-(2-methoxyethoxy)ethoxy)ethyl))germane (4)

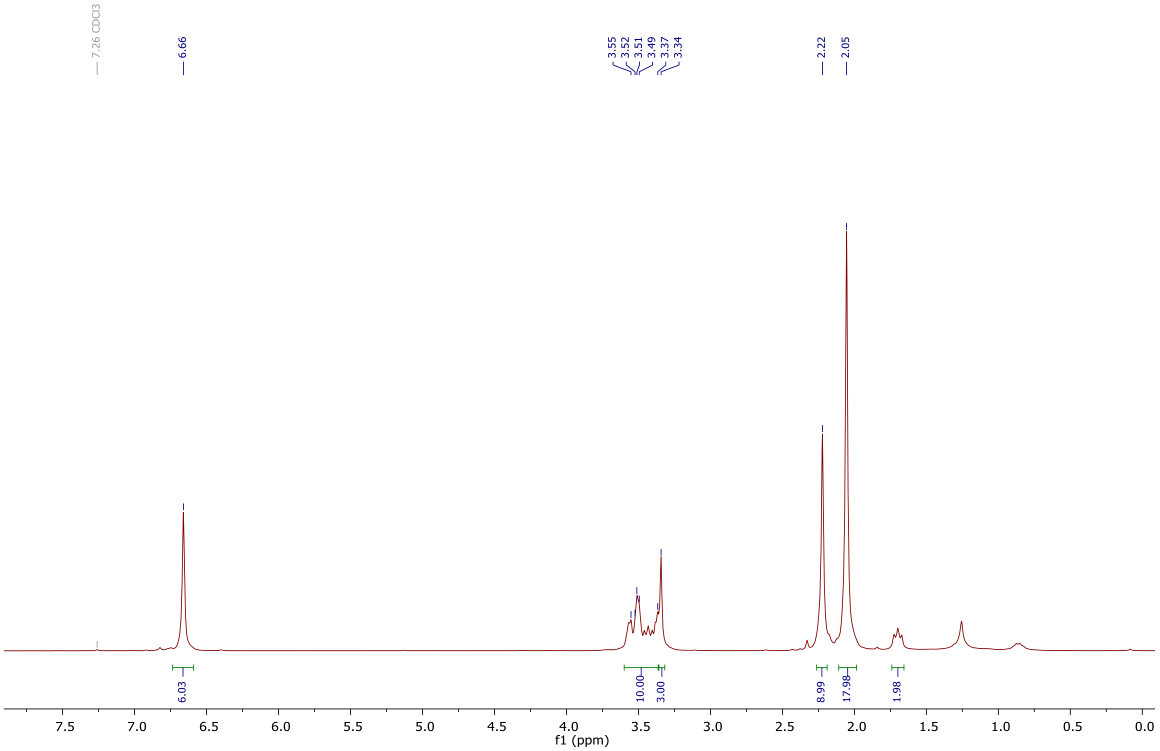

Figure S5. ^{1}H- spectrum of **4** ($CDCl_3$ solution, vs ext. TMS, ppm).

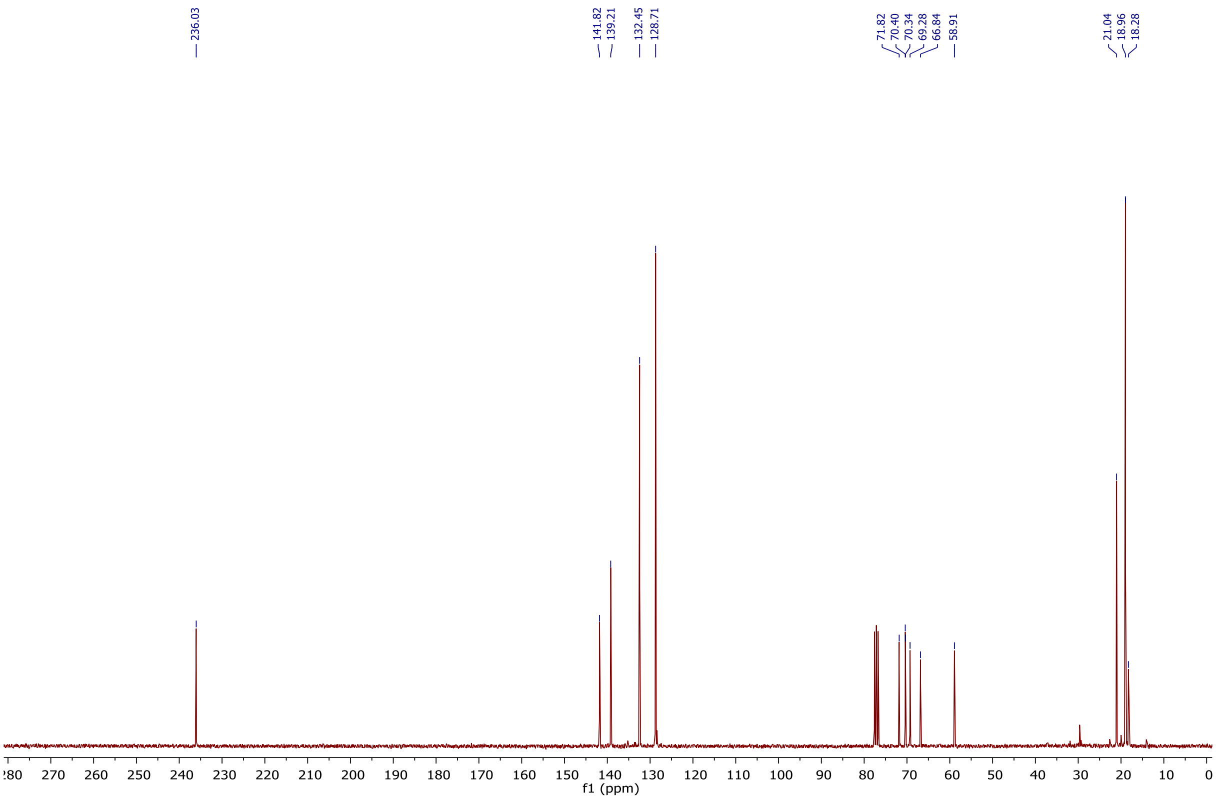

Figure S6. ^{13}C-NMR spectrum of **4** ($CDCl_3$ solution, vs ext. TMS, ppm).

Tris(2,4,6-trimethylbenzoyl)-(2-(2-(2-(2-(2-methoxyethoxy)ethoxy)ethoxy)ethyl))germane (5)

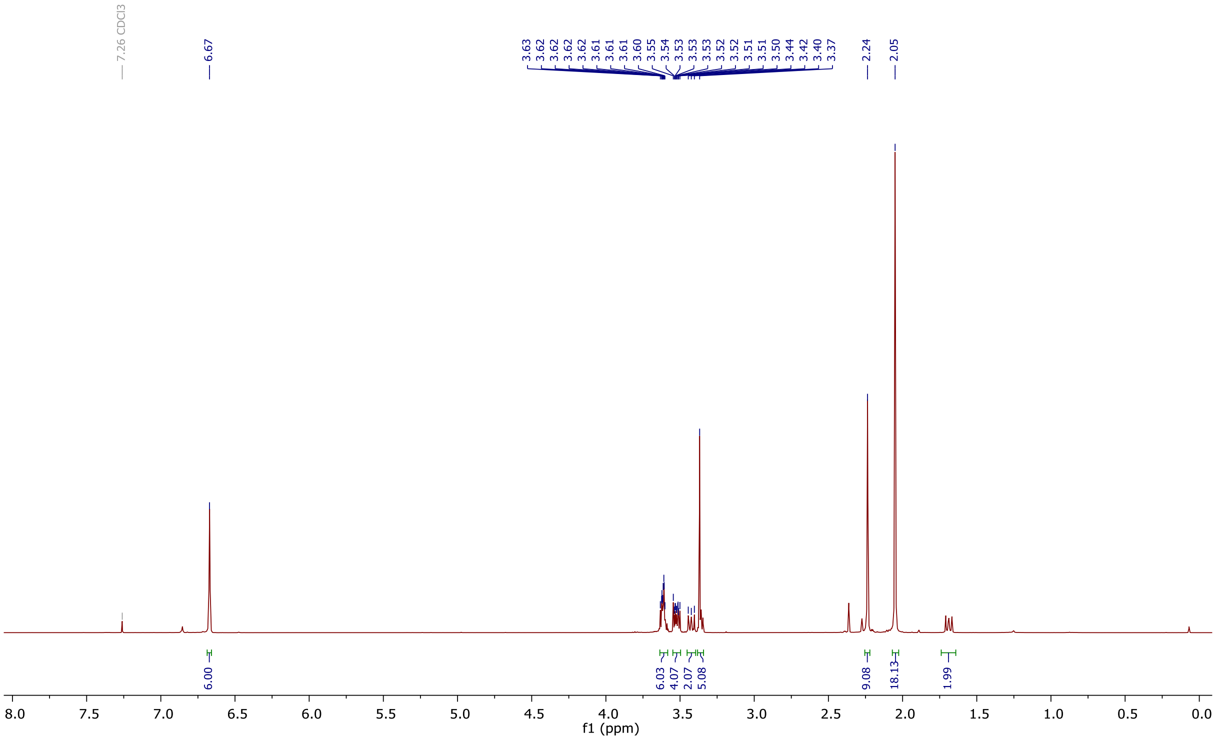

Figure S7. ^{1}H- spectrum of **5** ($CDCl_3$ solution, vs ext. TMS, ppm).

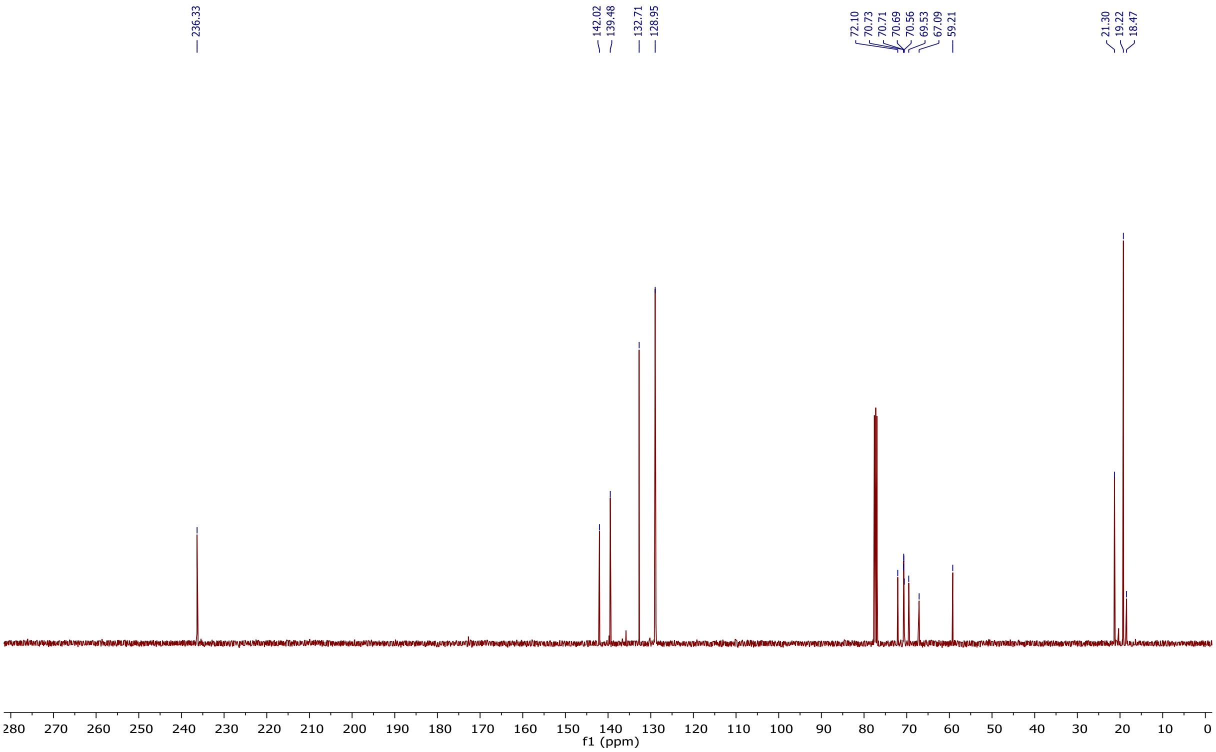

Figure S8. ^{13}C-NMR spectrum of **5** ($CDCl_3$ solution, vs ext. TMS, ppm).

Methyl 4-(2-(2-(2-(2-methoxyethoxy)ethoxy)ethoxy)ethyl) benzoate (6a)

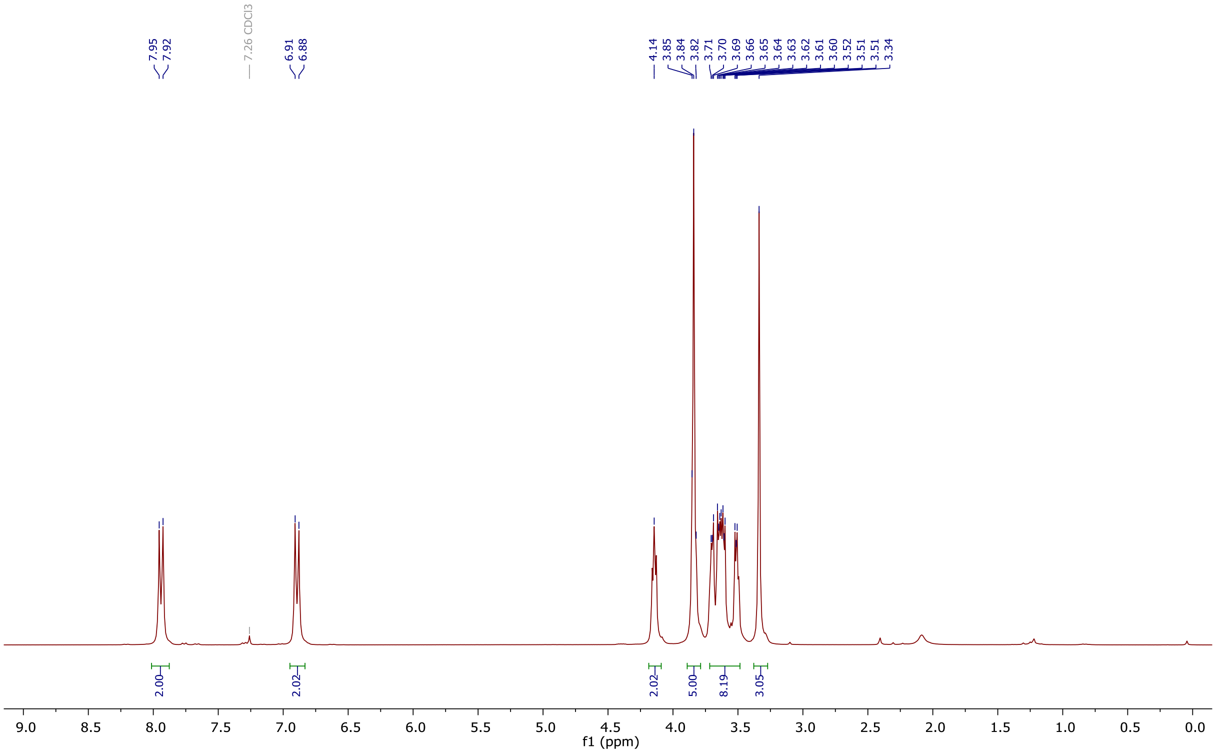

Figure S9. ^{1}H- spectrum of **6a** ($CDCl_3$ solution, vs ext. TMS, ppm).

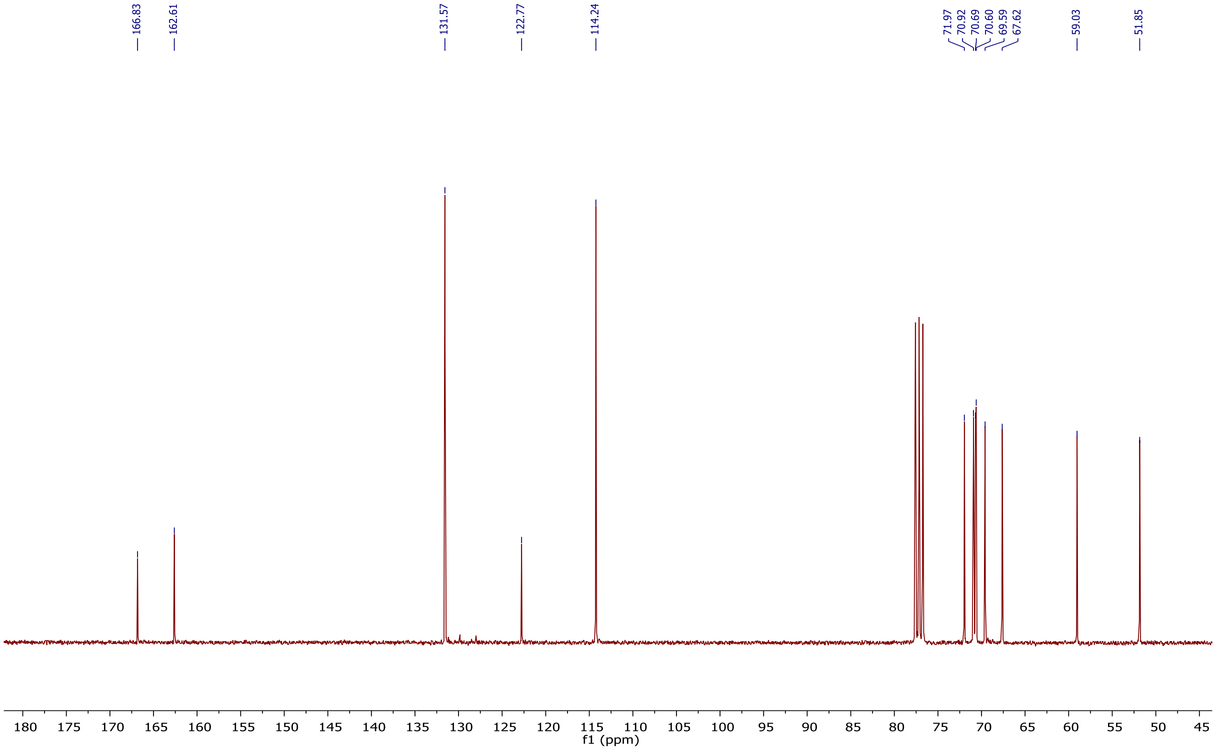

Figure S10. ^{13}C-NMR spectrum of **6a** ($CDCl_3$ solution, vs ext. TMS, ppm).

4-(2-(2-(2-(2-Methoxyethoxy)ethoxy)ethoxy)ethyl)benzoic acid (6b)

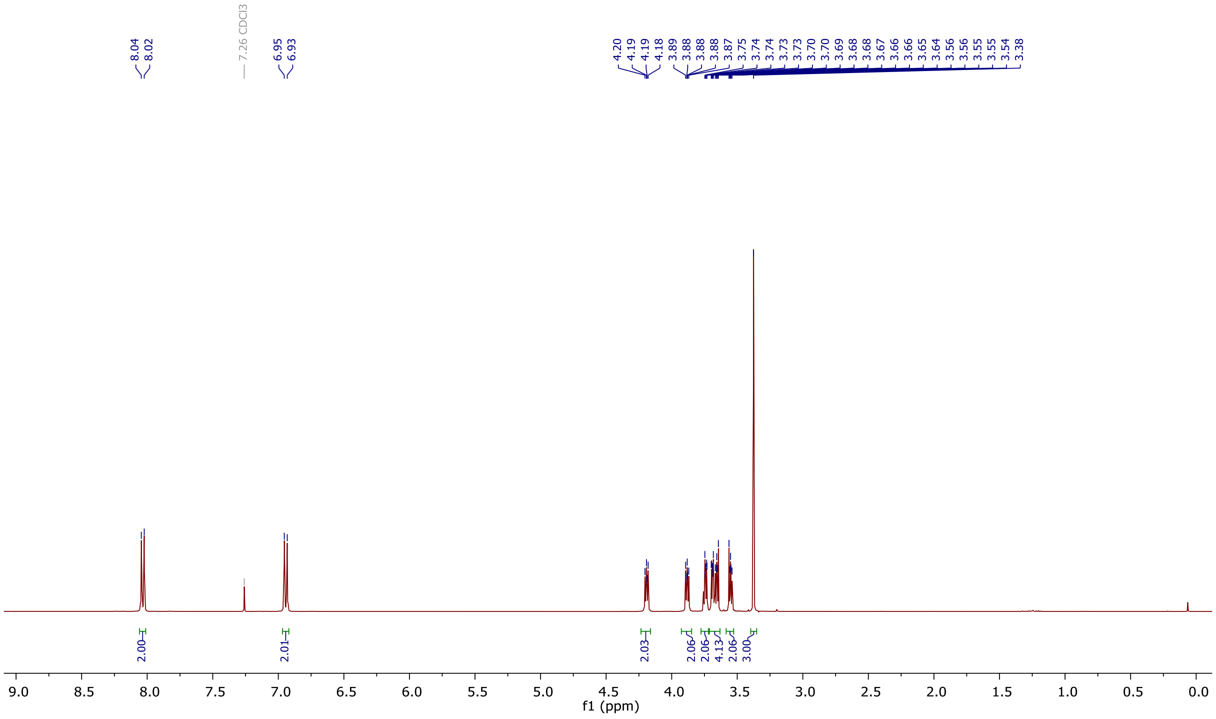

Figure S11. ^{1}H- spectrum of **6b** ($CDCl_3$ solution, vs ext. TMS, ppm).

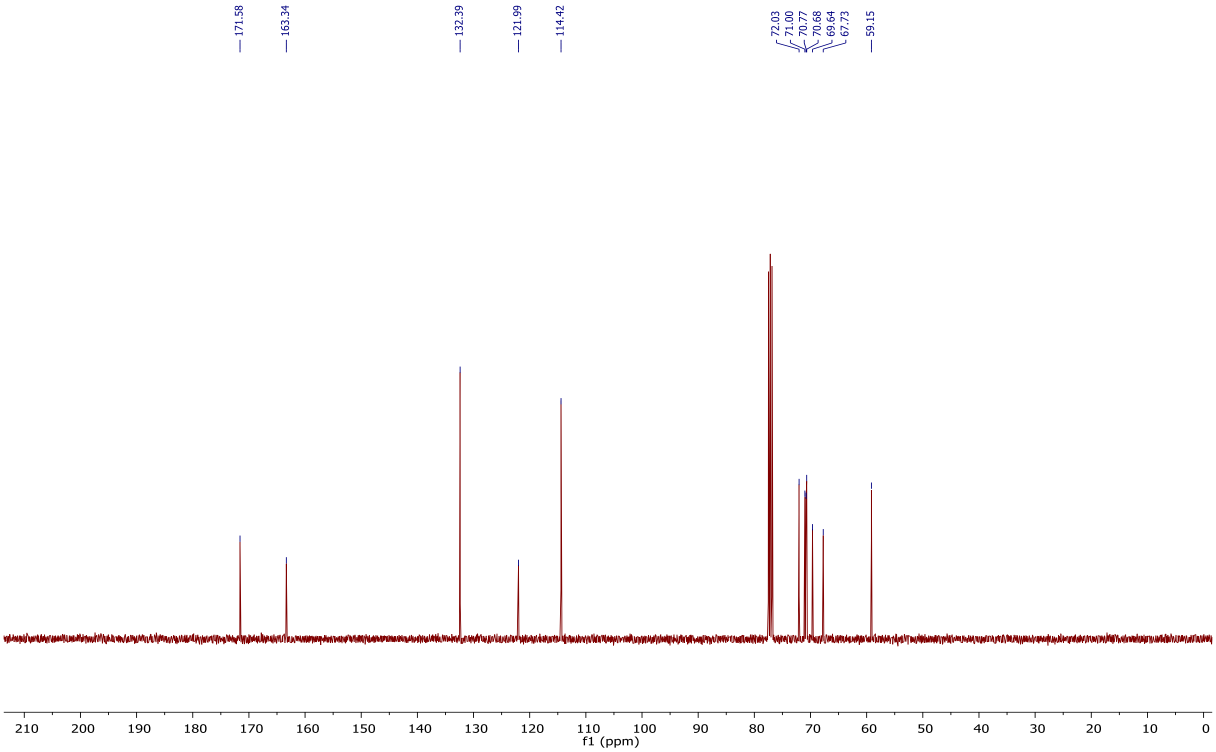

Figure S12. ^{13}C-NMR spectrum of **6b** ($CDCl_3$ solution, vs ext. TMS, ppm).

4-(2-(2-(2-(2-Methoxyethoxy)ethoxy)ethoxy)ethyl)benzoyl fluoride (6c)

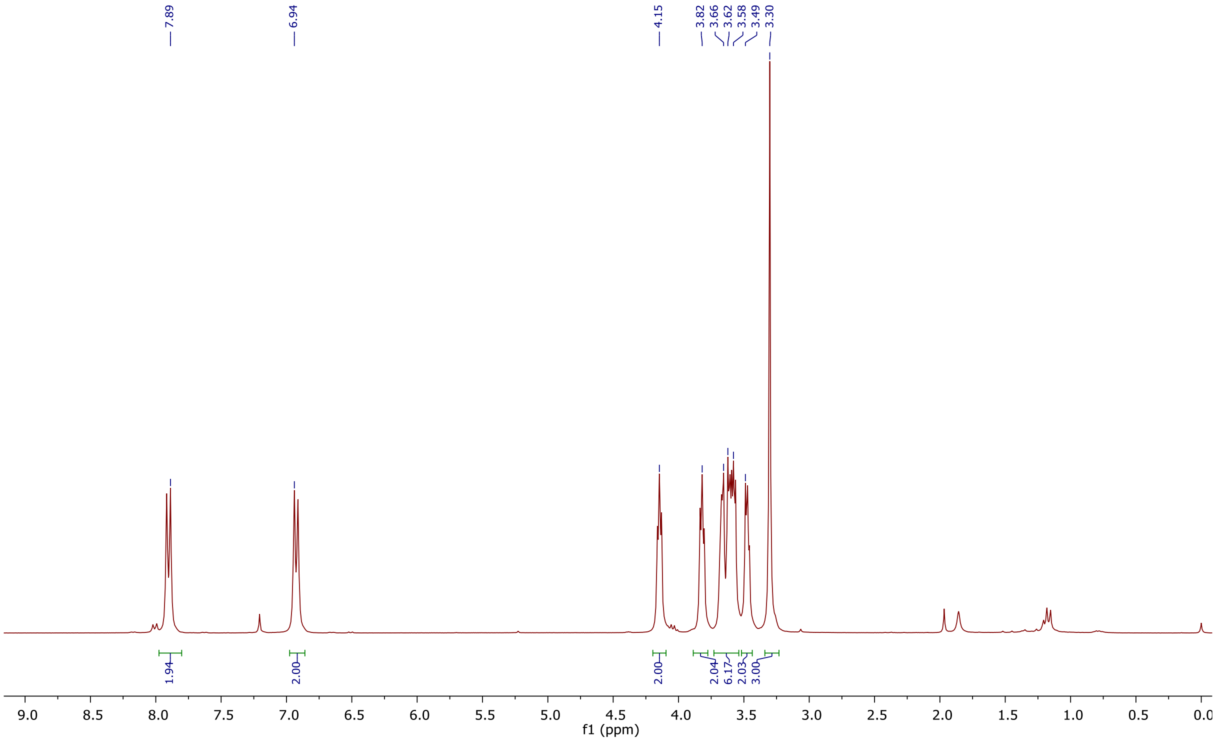

Figure S13. ^{1}H- spectrum of **6c** ($CDCl_3$ solution, vs ext. TMS, ppm).

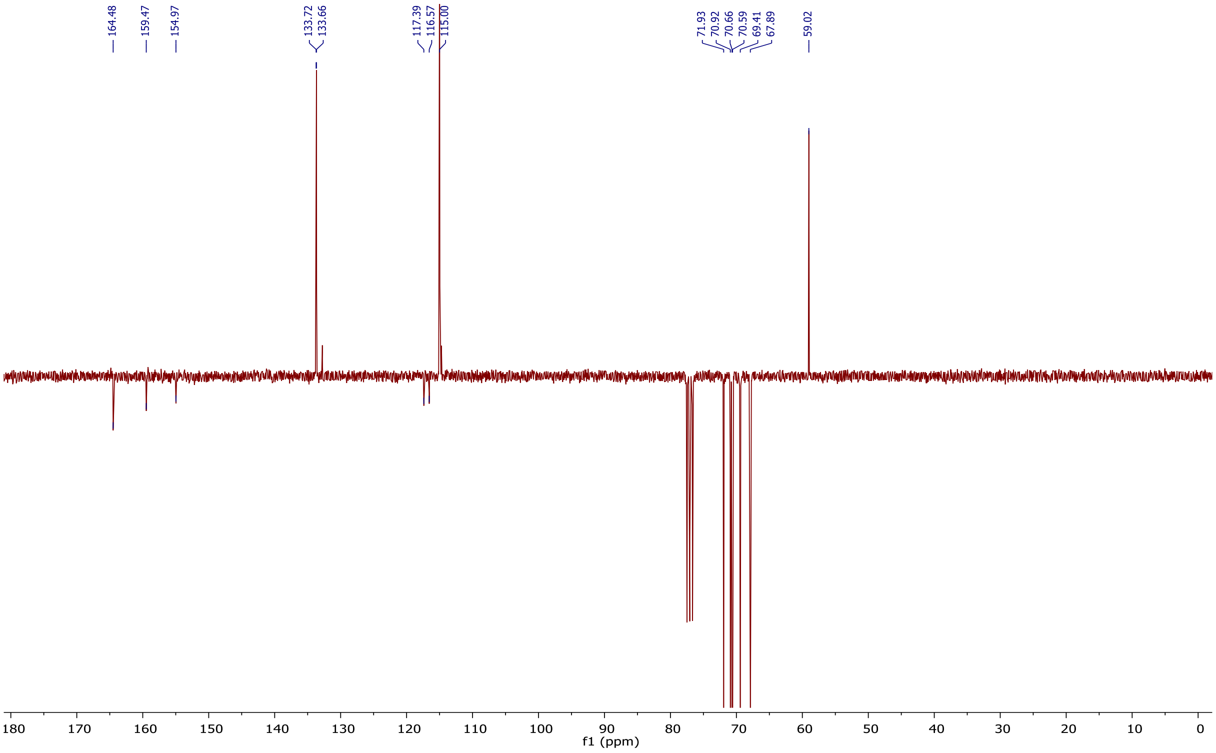

Figure S14. APT-NMR spectrum of **6c** ($CDCl_3$ solution, vs ext. TMS, ppm).

4-(2-(2-(2-(2-Methoxyethoxy)ethoxy)ethoxy)ethyl)benzoyl-tris(2,4,6-trimethylbenzoyl)germane (7)

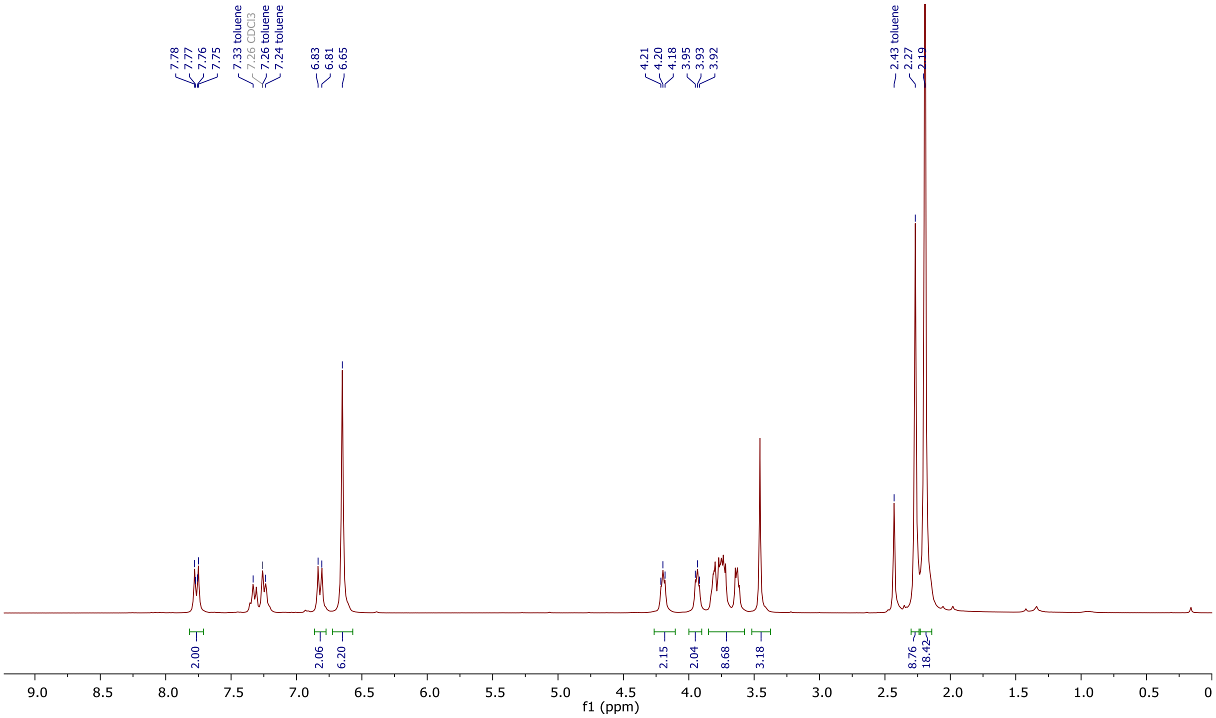

Figure S15. ^{1}H- spectrum of **7** ($CDCl_3$ solution, vs ext. TMS, ppm).

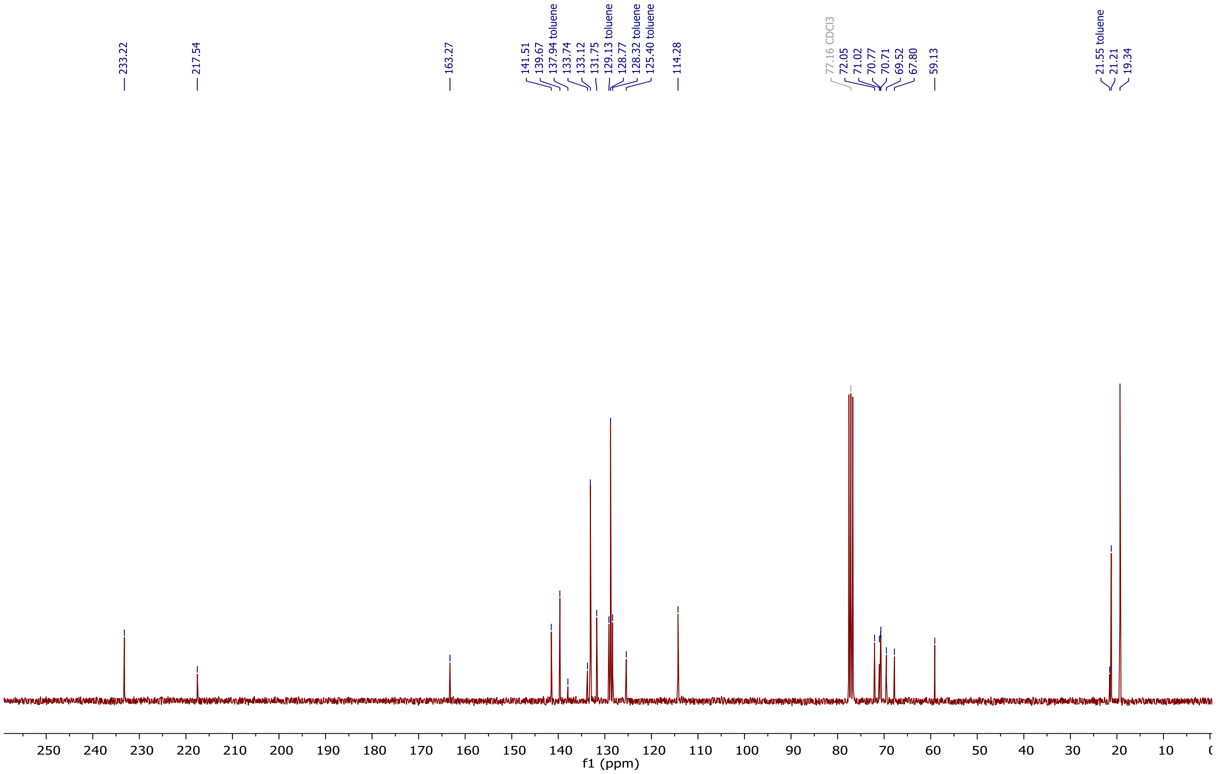

Figure S16. ^{13}C-NMR spectrum of **7** ($CDCl_3$ solution, vs ext. TMS, ppm).

Bis(4-(2-(2-(2-(2-Methoxyethoxy)ethoxy)ethoxy)ethyl)benzoyl)-bis(2,4,6-trimethylbenzoyl)germane (8)

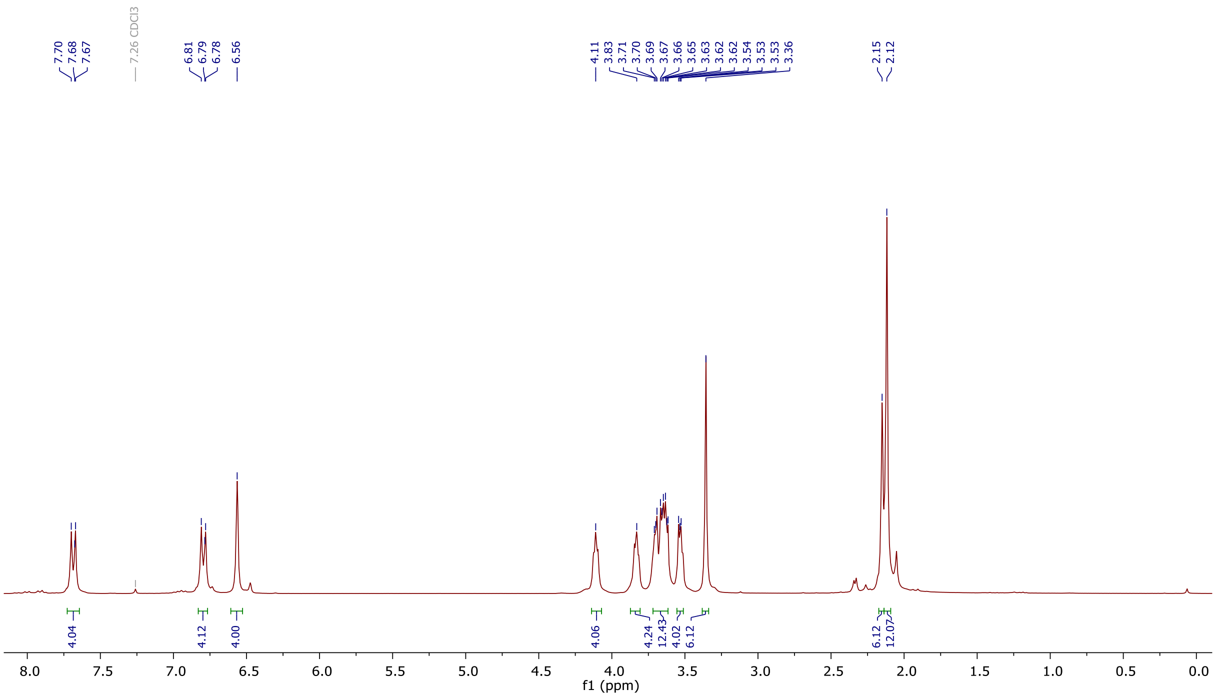

Figure S17. ^{1}H- spectrum of **8** ($CDCl_3$ solution, vs ext. TMS, ppm).

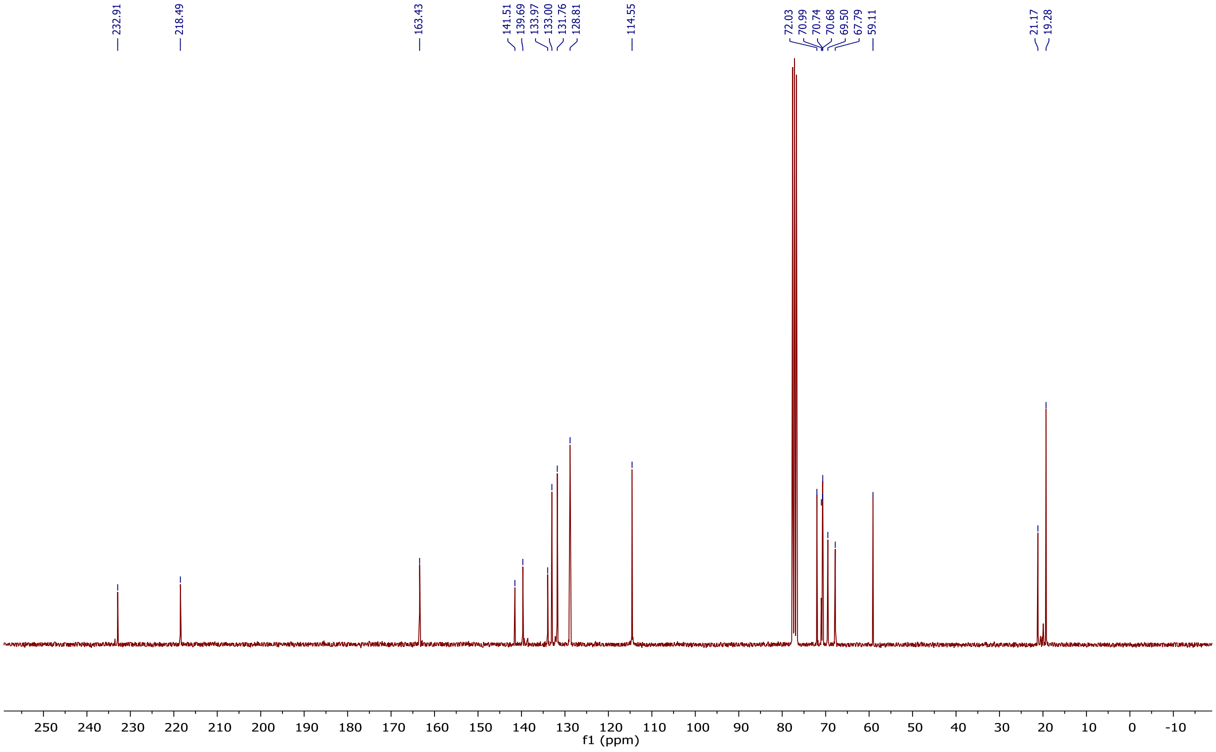

Figure S18. ^{13}C-NMR spectrum of **8** ($CDCl_3$ solution, vs ext. TMS, ppm).

Tetra(4-(2-(2-(2-(2-methoxyethoxy)ethoxy)ethoxy)ethyl)benzoyl)germane (6d)

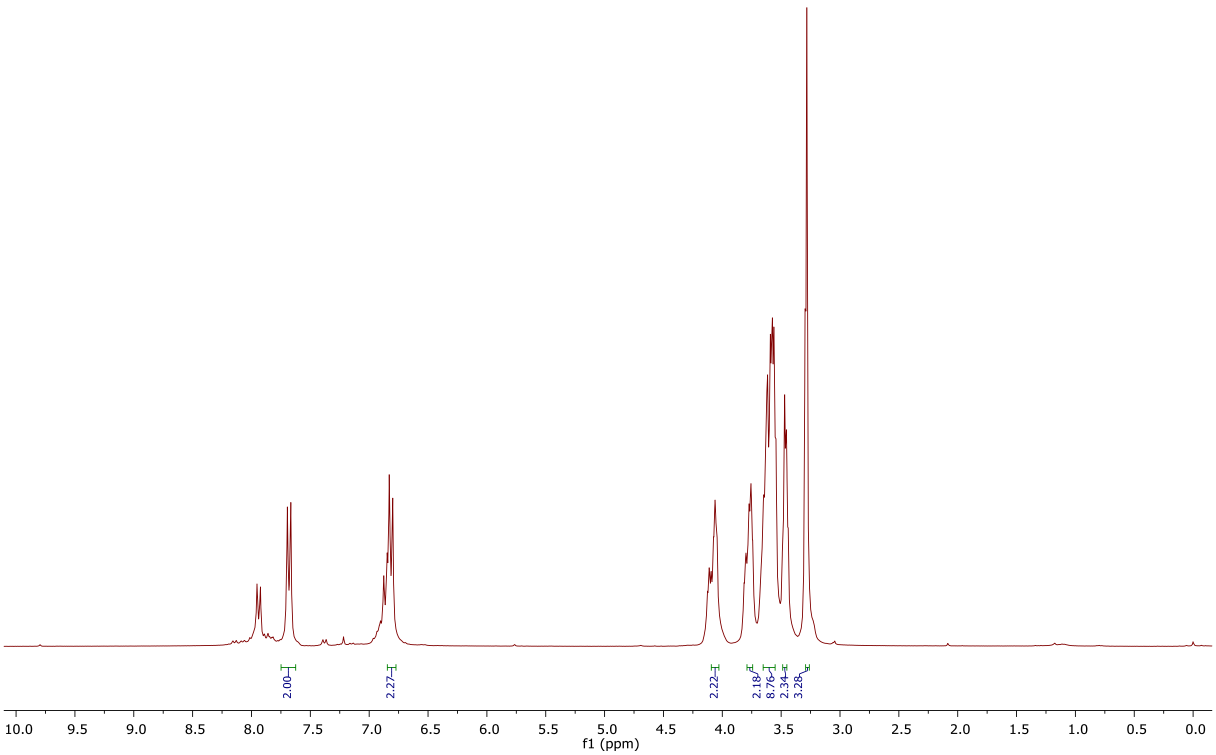

Figure S19. ^{1}H- spectrum of **6d** ($CDCl_3$ solution, vs ext. TMS, ppm).

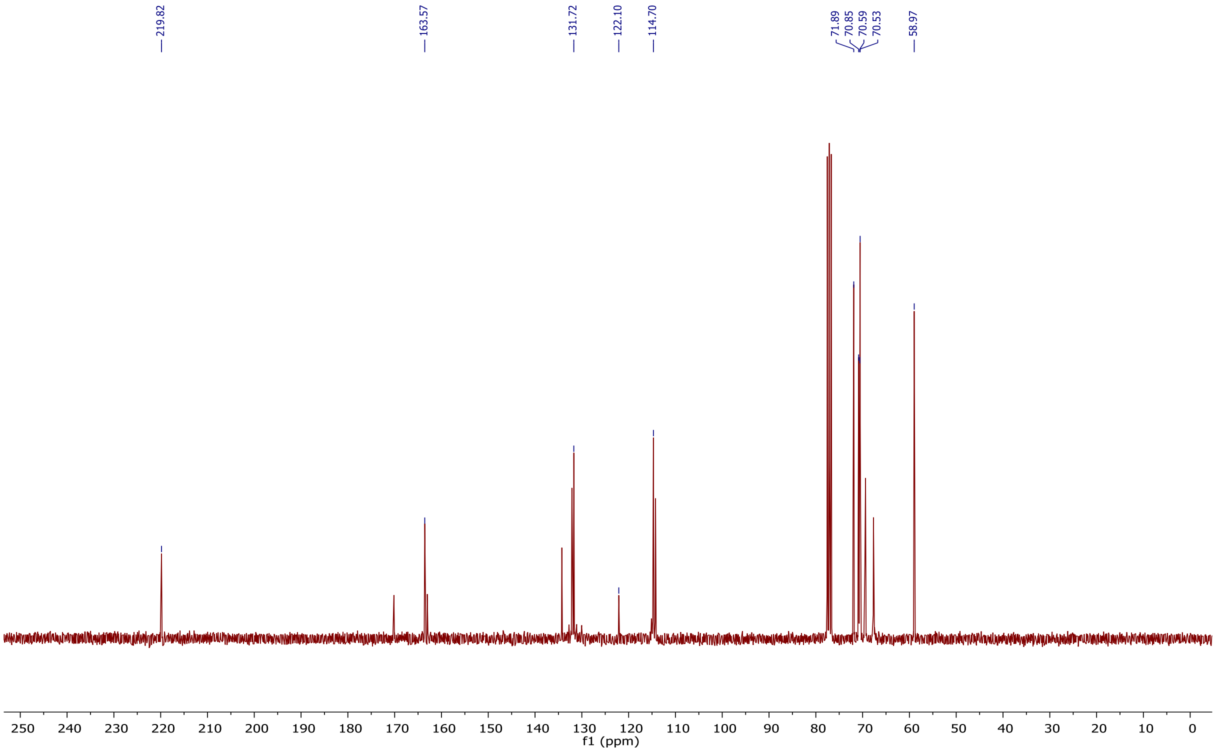

Figure S20. ^{13}C-NMR spectrum of **6d** ($CDCl_3$ solution, vs ext. TMS, ppm).

UV-Vis Spectroscopy

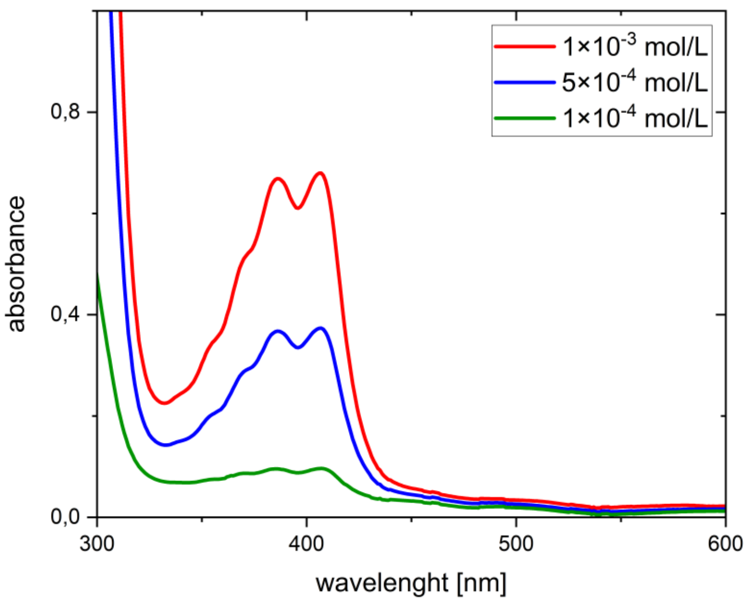

Figure S21. UV-vis Spectrum of **2** at different concentrations, measured in THF.

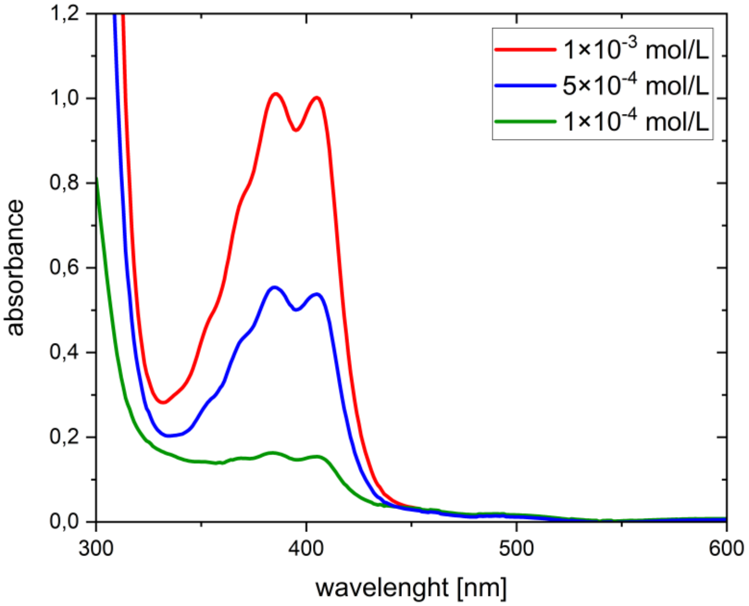

Figure S22. UV-vis Spectrum of **3** at different concentrations, measured in THF.

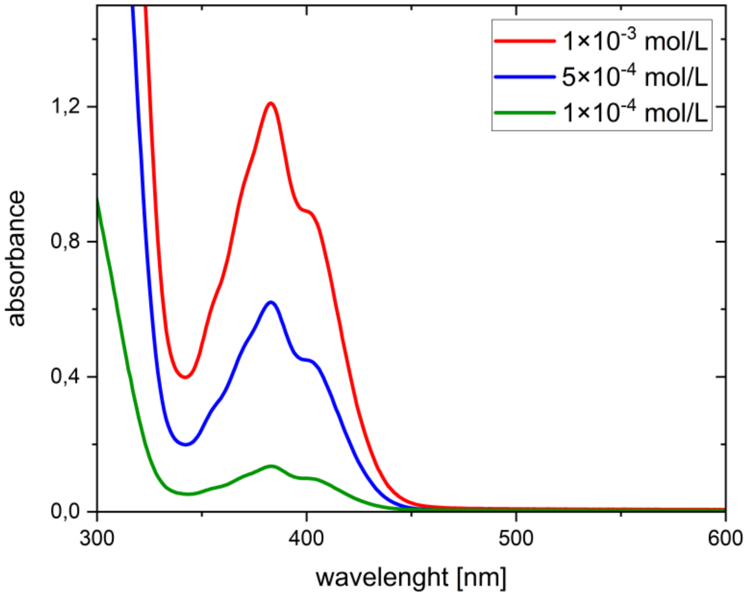

Figure S23. UV-vis Spectrum of **4** at different concentrations, measured in THF.

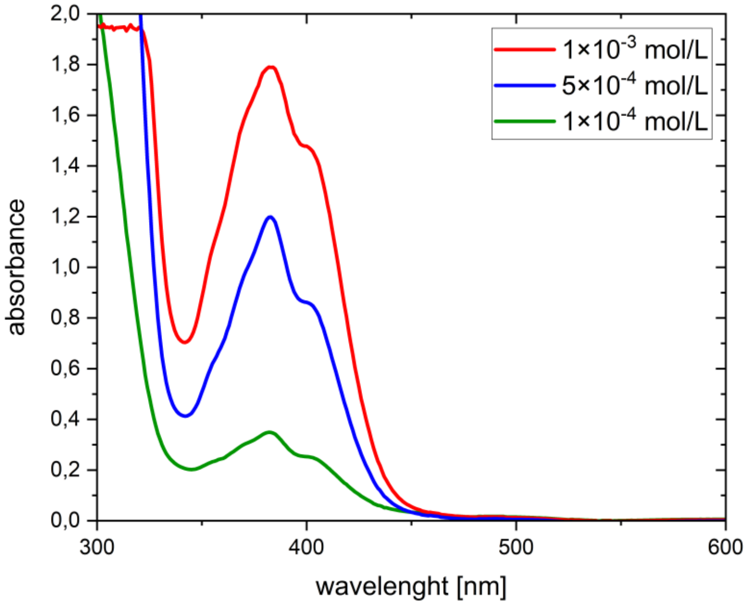

Figure S24. UV-vis Spectrum of 5 at different concentrations, measured in THF.

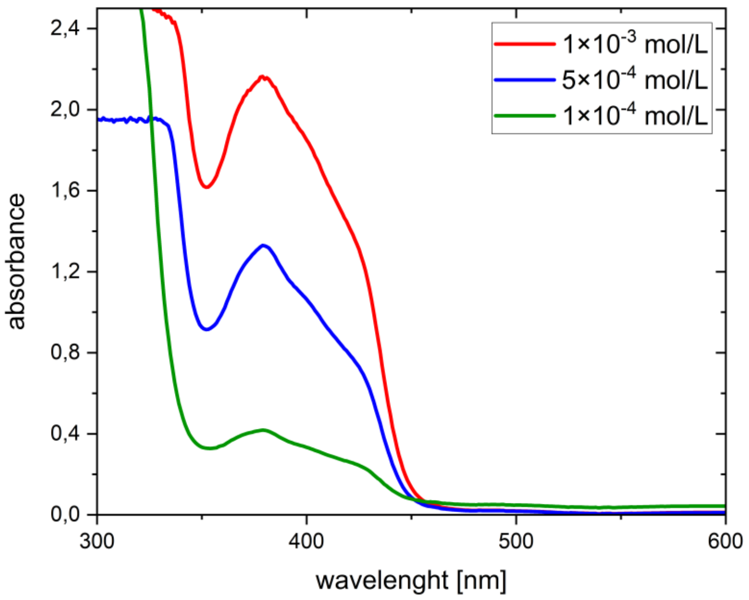

Figure S25. UV-vis Spectrum of **7** at different concentrations, measured in THF.

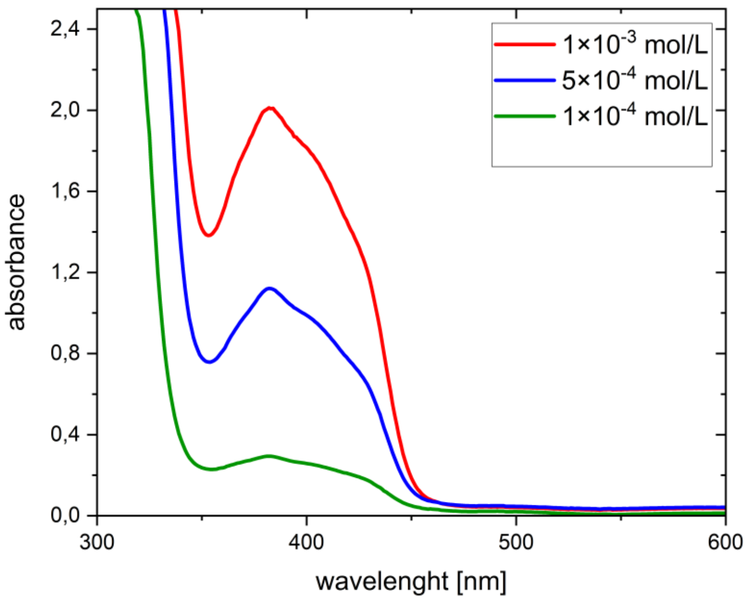

Figure S26. UV-vis Spectrum of **8** at different concentrations, measured in THF.

CIDNP Experiments

The ^{1}H NMR and CIDNP spectra were recorded using a Bruker Avance 200 MHz spectrometer (see Figure S27 for set-up). For Photo-CIDNP experiments, two irradiation sources were utilized: a Quantel Brilliant B Nd:YAG laser operating at its third harmonic (355 nm) and a Hamamatsu LC4 high-pressure Hg/Xe lamp. In laser-CIDNP experiments, a presaturation pulse train (waltz16) was applied before the laser flash (8 ns) to suppress background equilibrium NMR transitions. In UV lamp CIDNP experiments, the lamp flash duration was 300 ms, and the CIDNP spectra were obtained by subtracting spectra recorded with and without light irradiation. All samples were bubbled with dry nitrogen gas through for 5 minutes prior to use to eliminate dissolved oxygen.

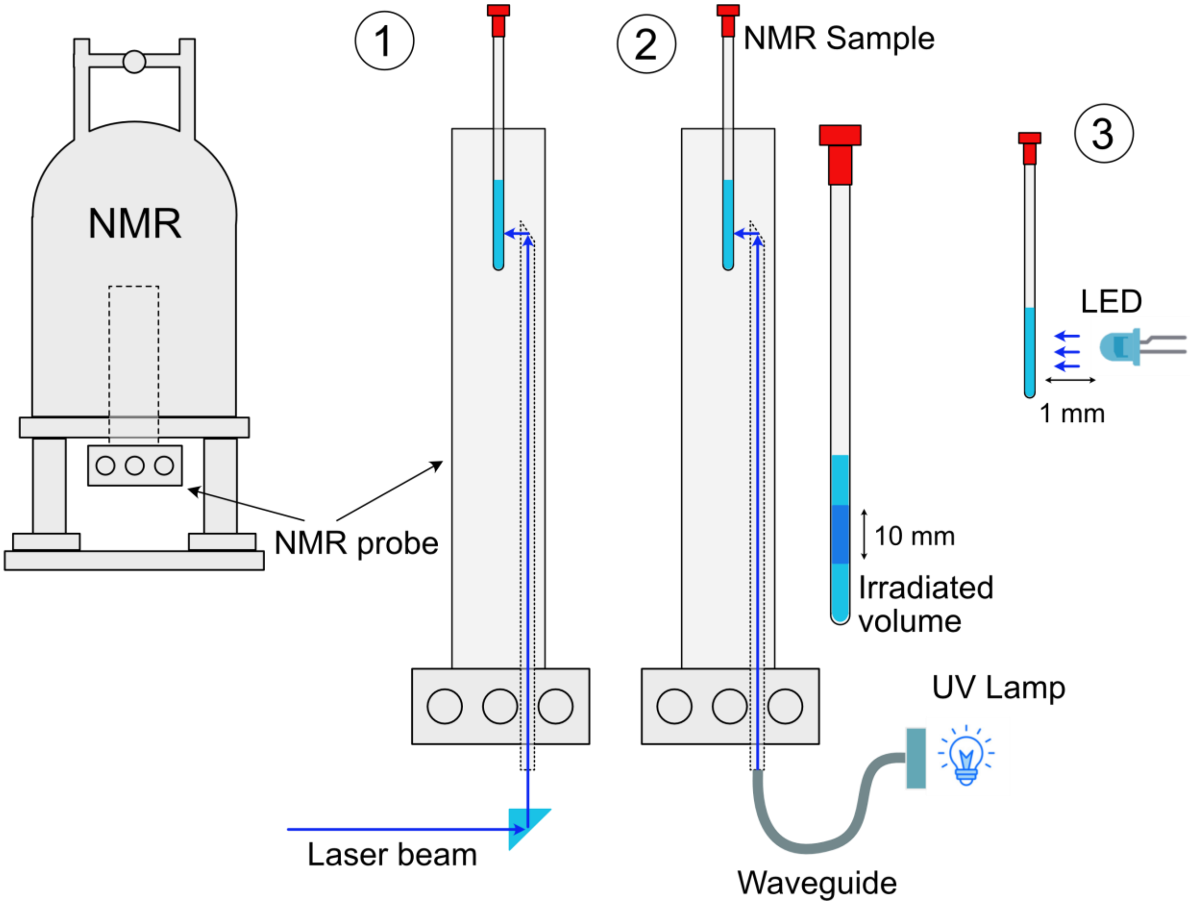

Figure S27. Schematic representation of different irradiation set-ups which were used throughout this study. 1. Laser CIDNP (355 nm, 8 ns) 2. UV-Lamp CIDNP (broadband, 300 ms, with filter) 3. LED irradiation ex situ (365 nm, 405 nm, 580 nm)

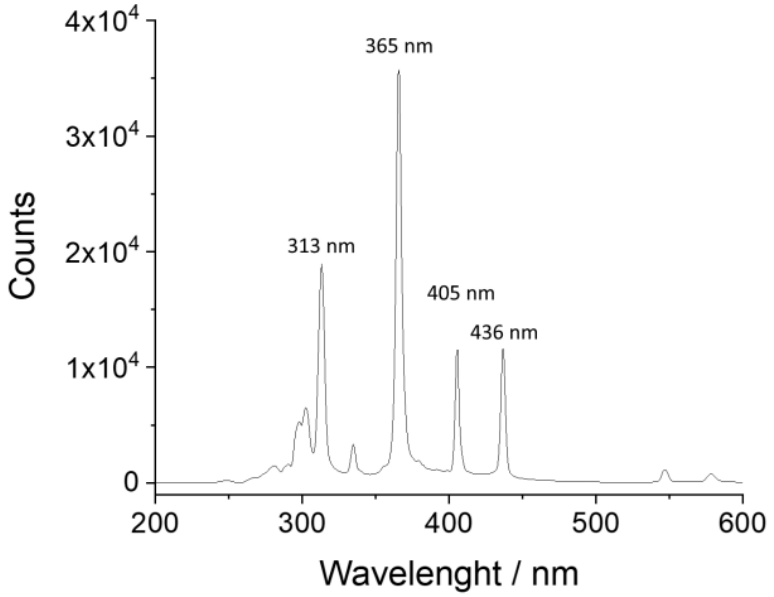

Figure S28. Emission spectrum of Hamamatsu LC4 lamp used in selected Photo-CIDNP experiments

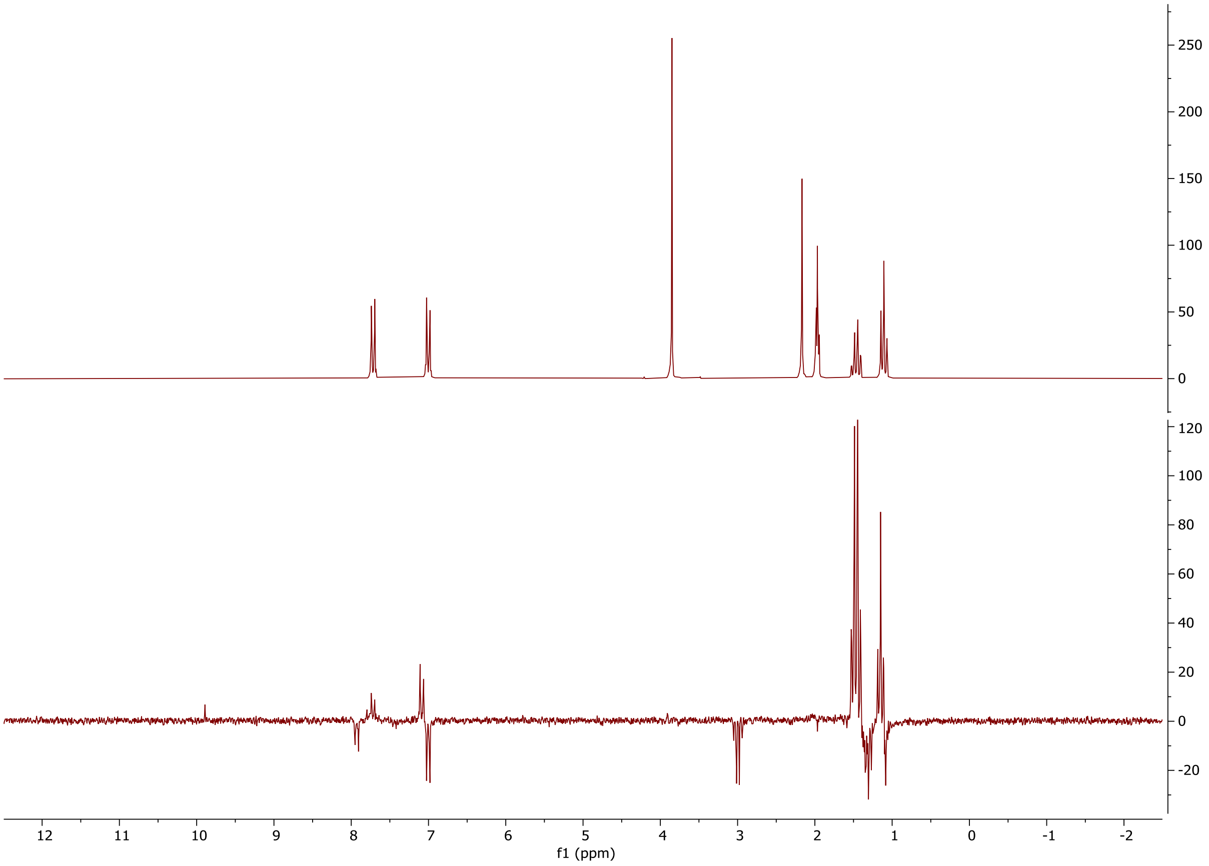

Figure S29. ^{1}H NMR (top) and photo-CIDNP (laser, 355 nm) spectra of **Ivocerin** (**1**) in Acetonitrile-d_3

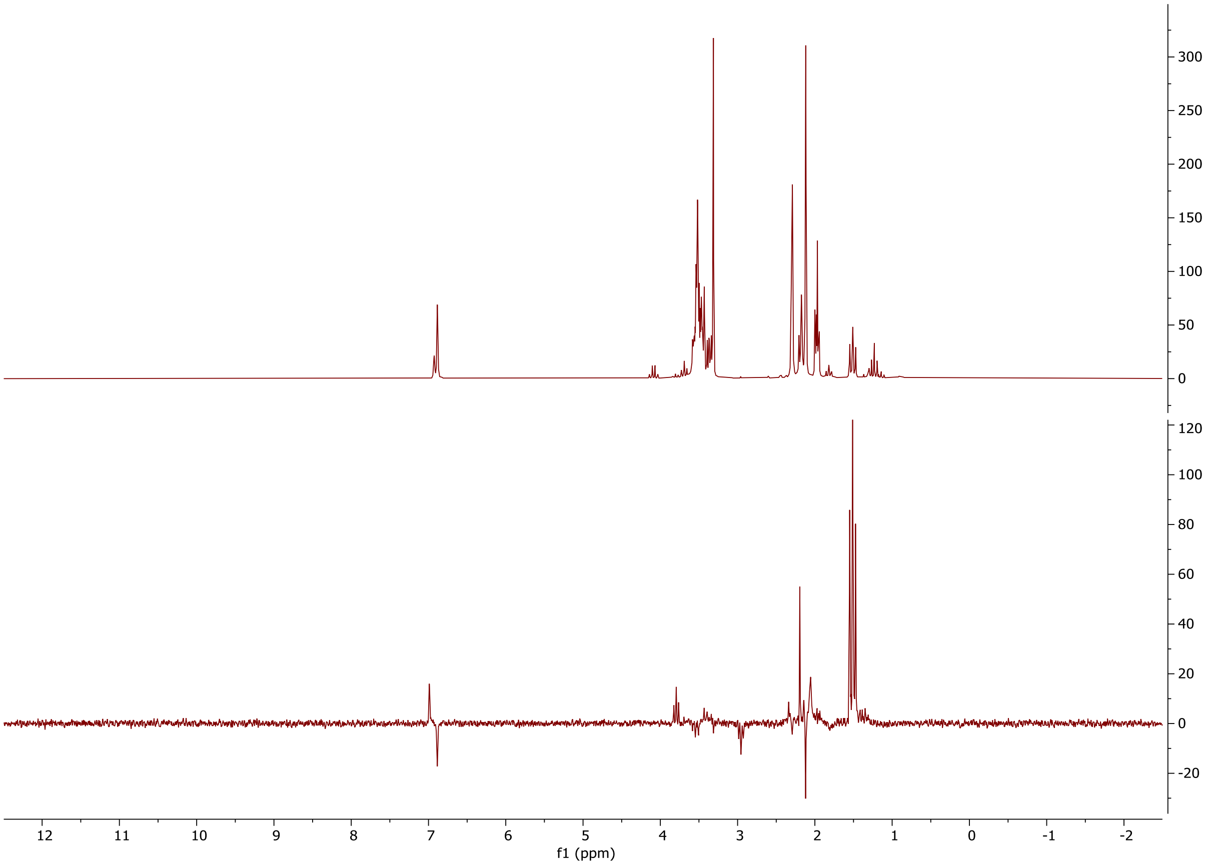

Figure S30. ^{1}H NMR (top) and photo-CIDNP (laser, 355 nm) spectra of **3** in Acetonitrile-d_3

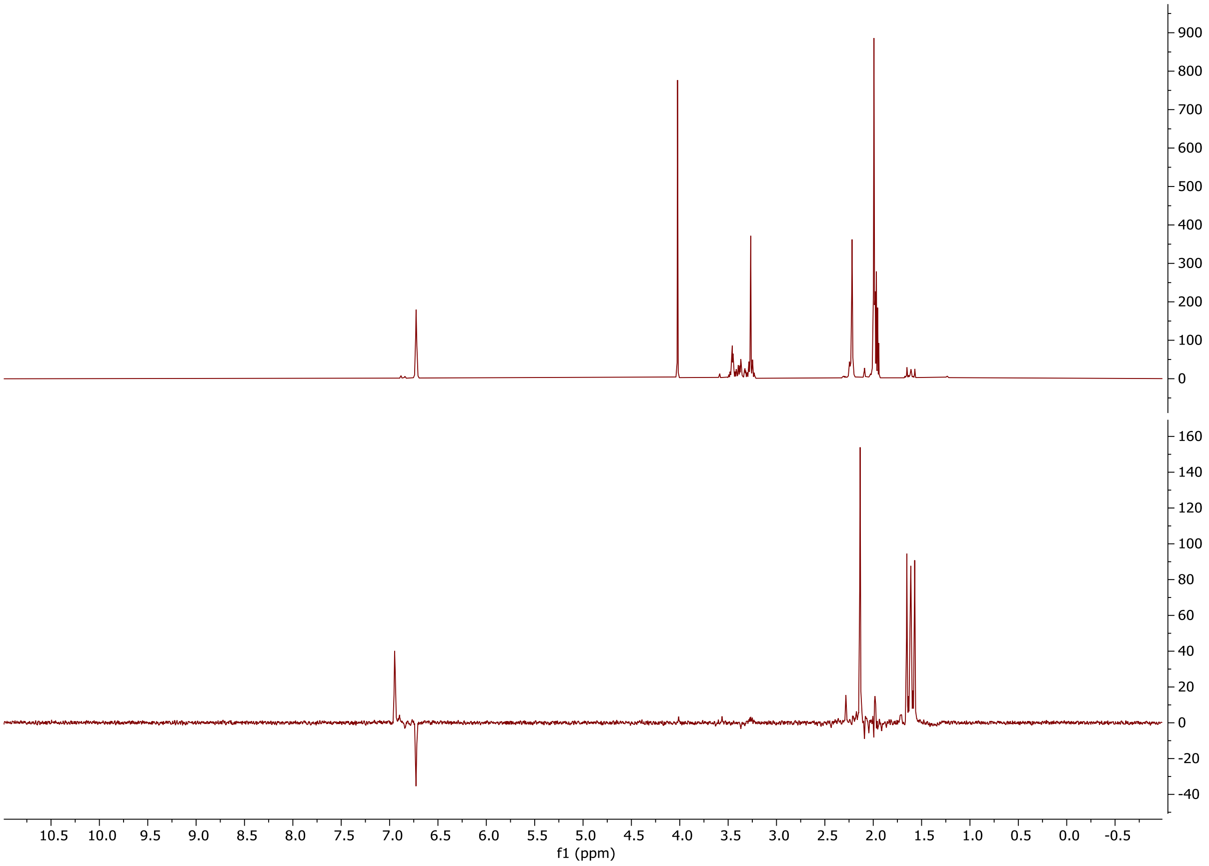

Figure S31. ^{1}H NMR (top) and photo-CIDNP (laser, 355 nm) spectra of **4** in Acetonitrile-d_3

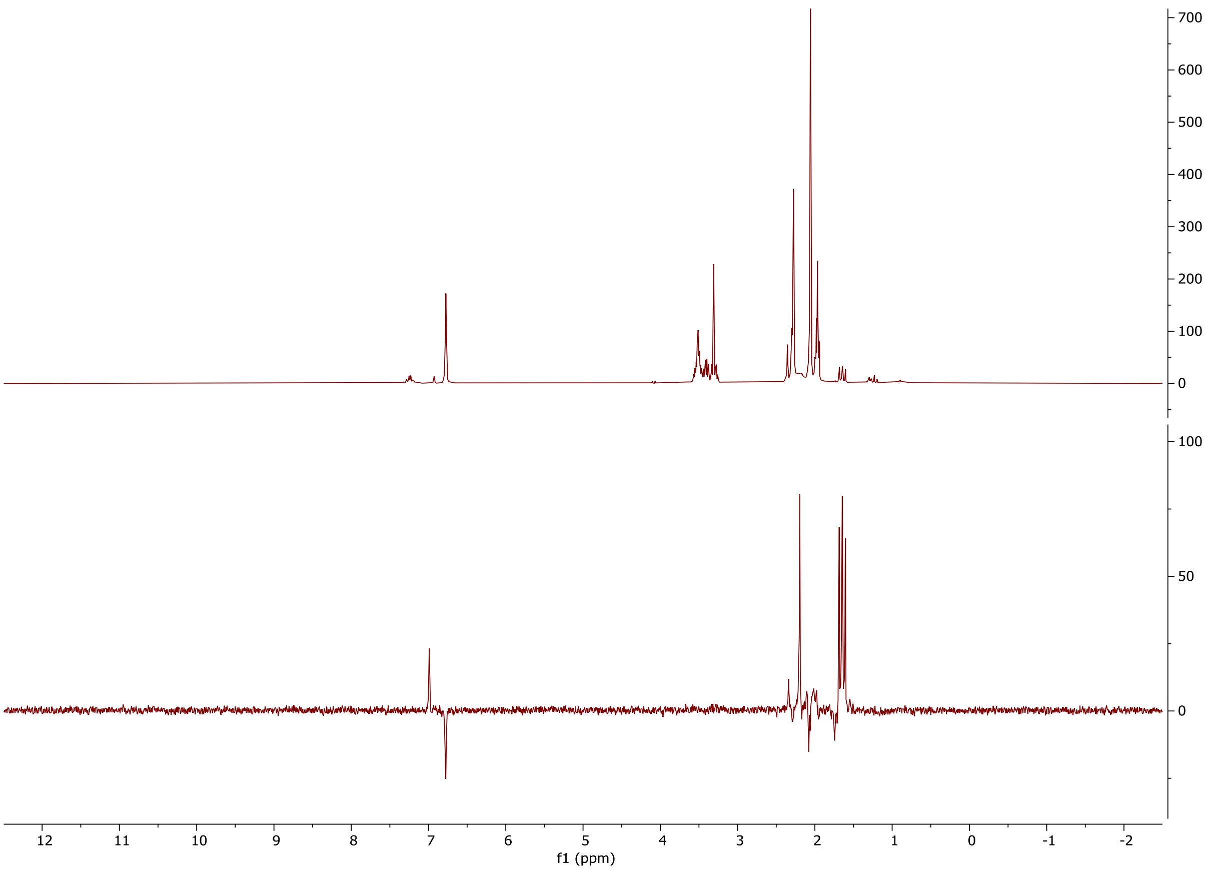

Figure S32. ^{1}H NMR (top) and photo-CIDNP (laser, 355 nm) spectra of **5** in Acetonitrile-d_3

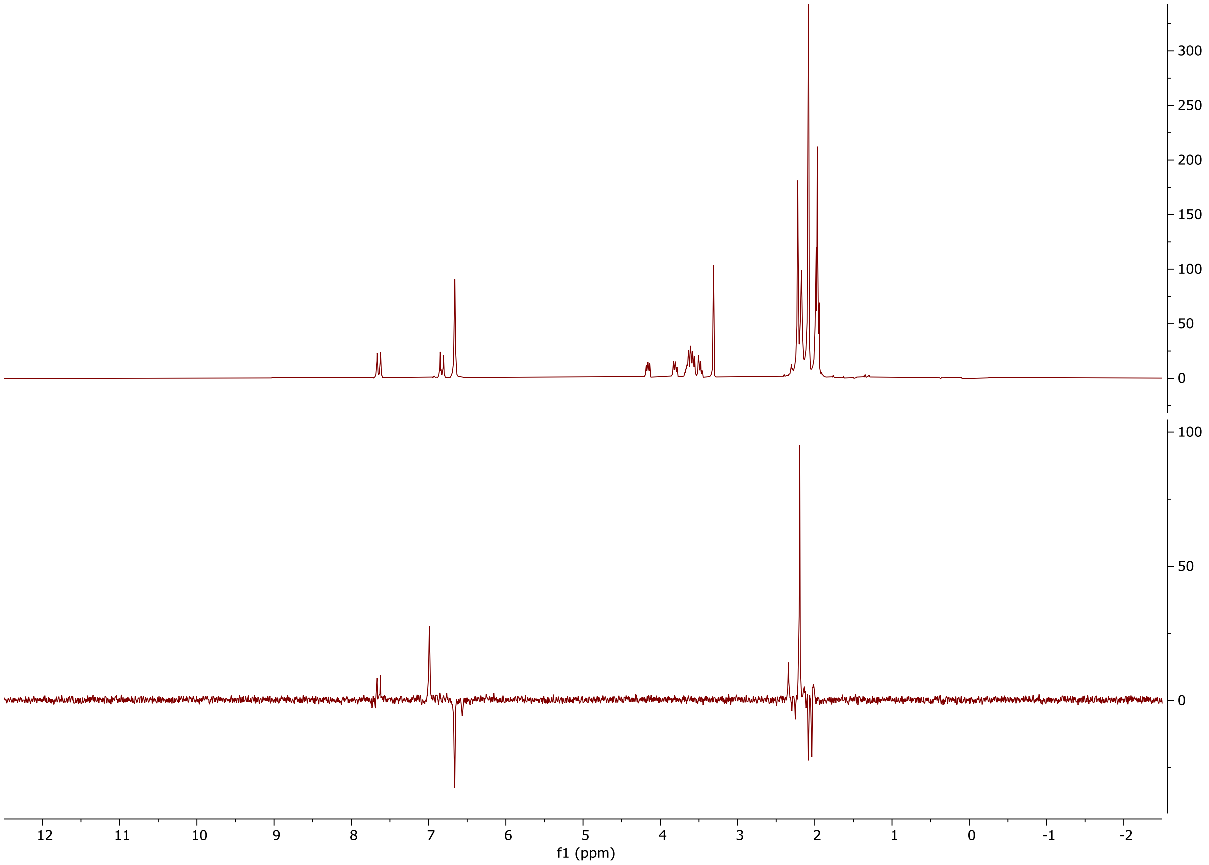

Figure S33. ^{1}H NMR (top) and photo-CIDNP (laser, 355 nm) spectra of **7** in Acetonitrile-d_3

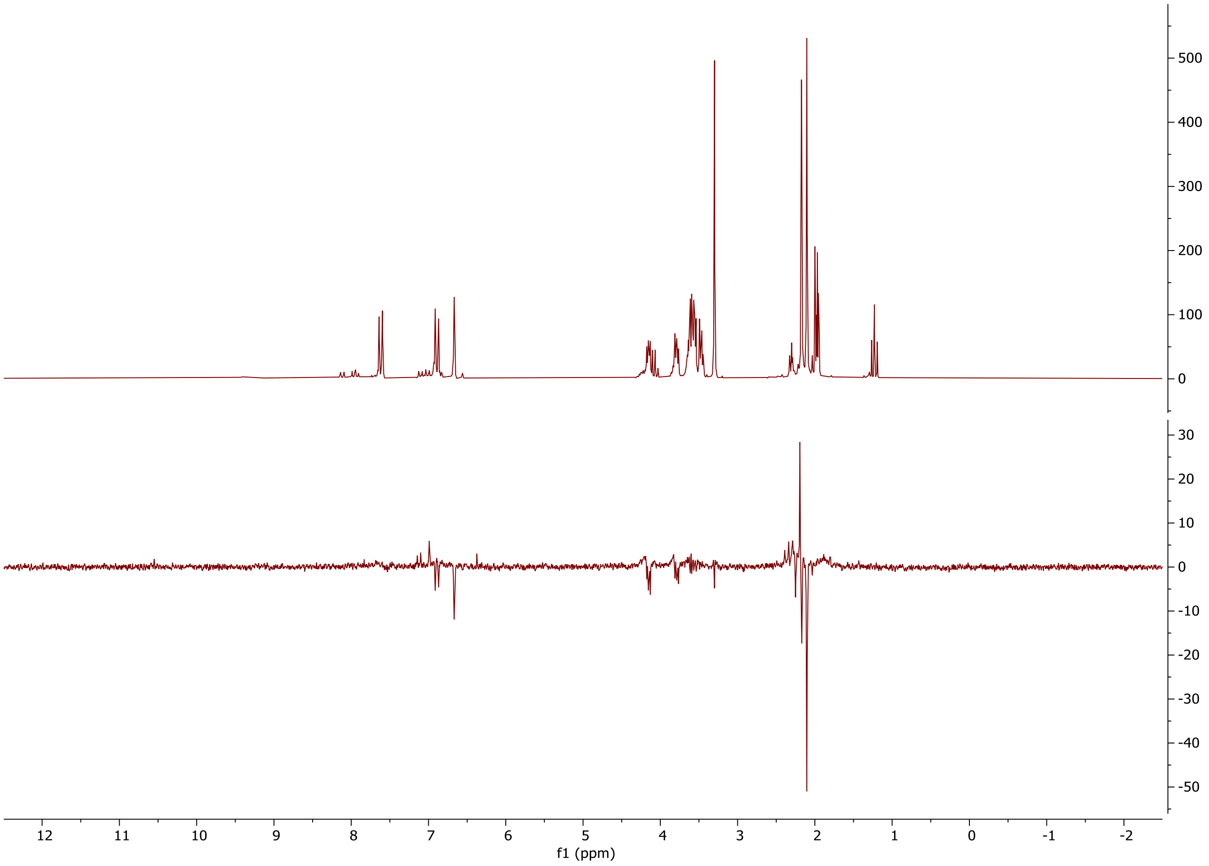

Figure S34. ^{1}H NMR (top) and photo-CIDNP (laser, 355 nm) spectra of **8** in Acetonitrile-d_3

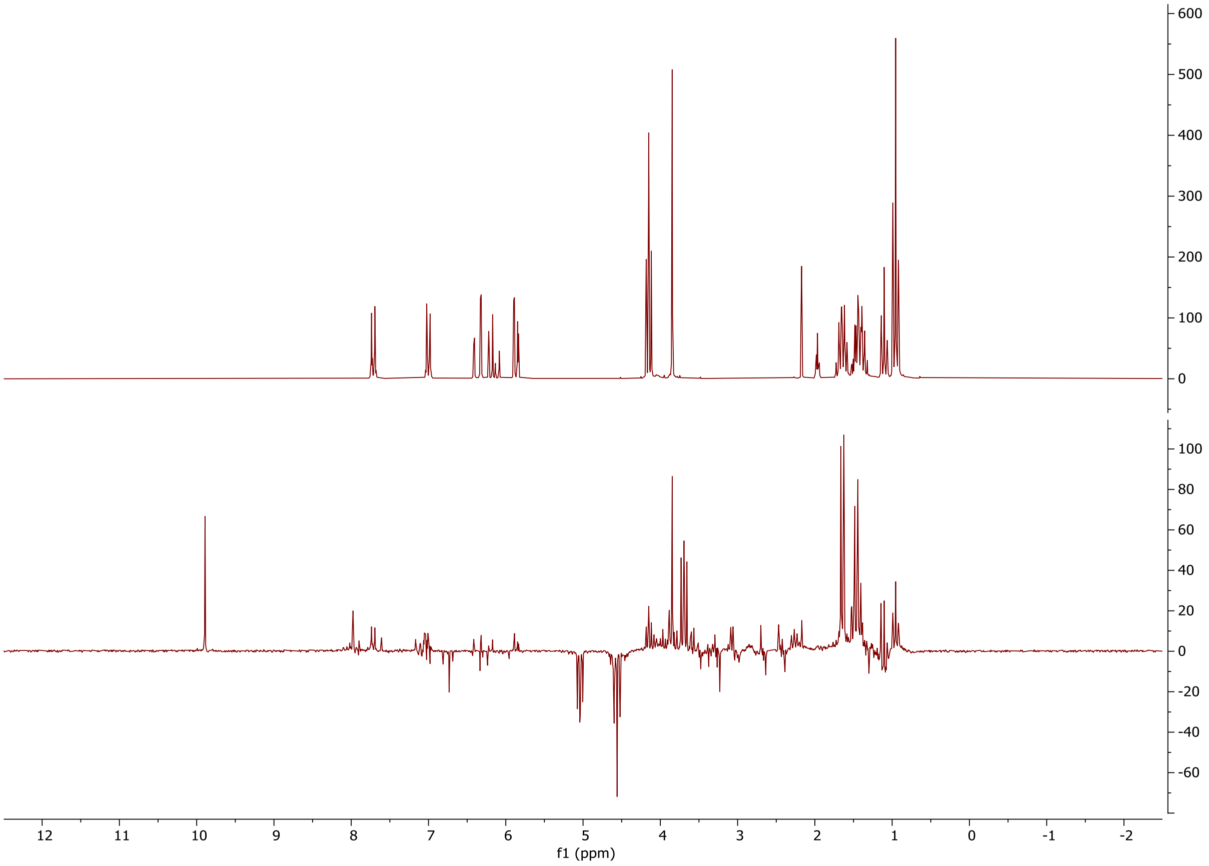

Figure S35. ^{1}H NMR (top) and photo-CIDNP (UV-vis lamp) spectra of **1** in Acetonitrile-d_3 in the presence of butyl acrylate.

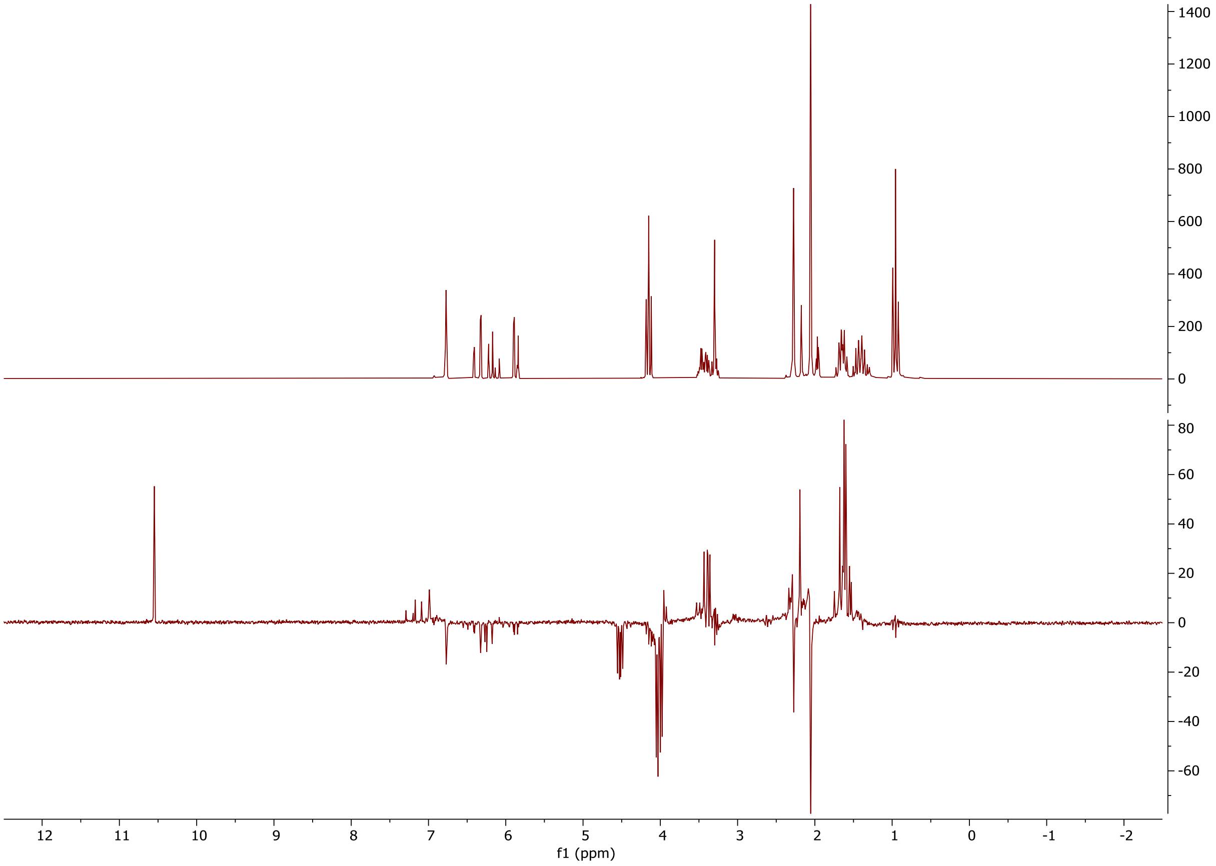

Figure S36. ^{1}H NMR (top) and photo-CIDNP (UV-vis lamp) spectra of **4** in Acetonitrile-d_3 in the presence of butyl acrylate.

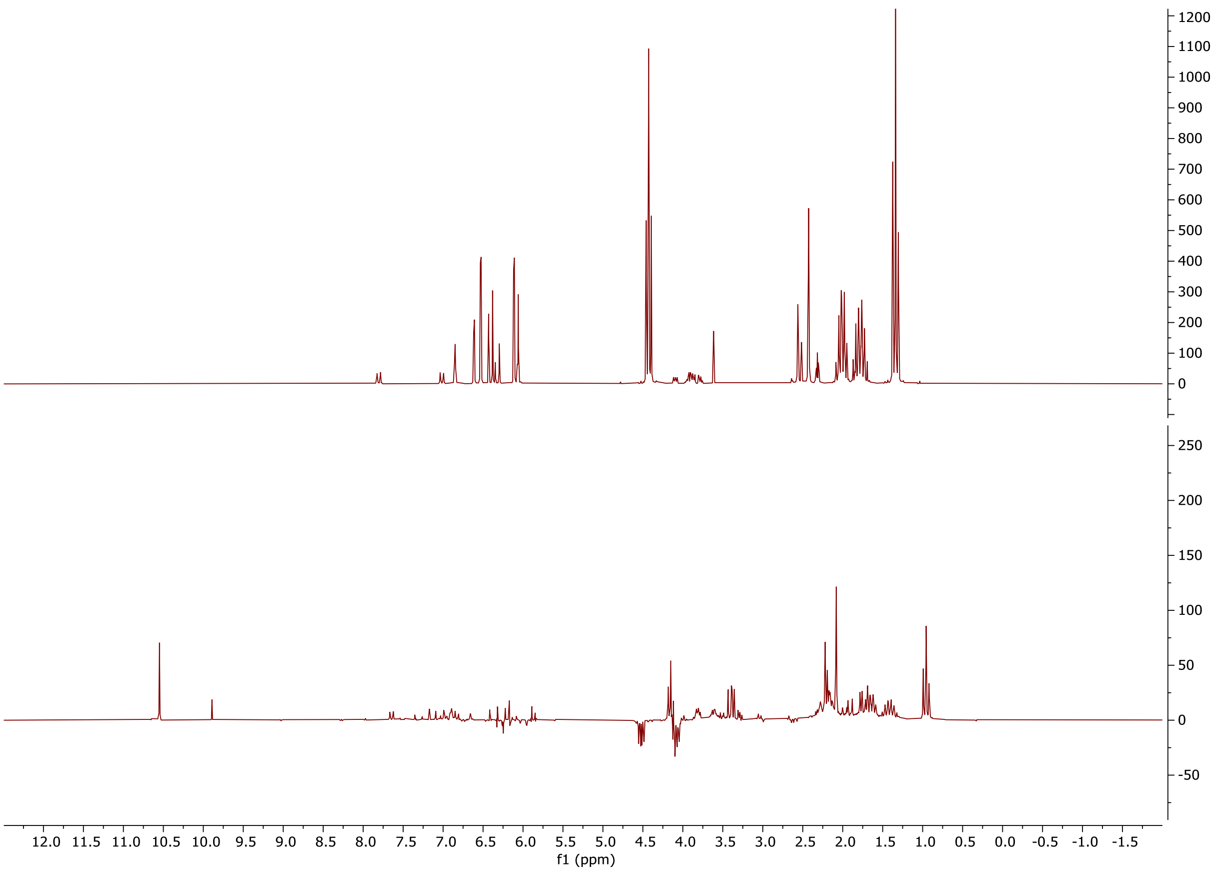

Figure S37. ^{1}H NMR (top) and photo-CIDNP (UV-vis lamp) spectra of **7** in Acetonitrile-d_3 in the presence of butyl acrylate.

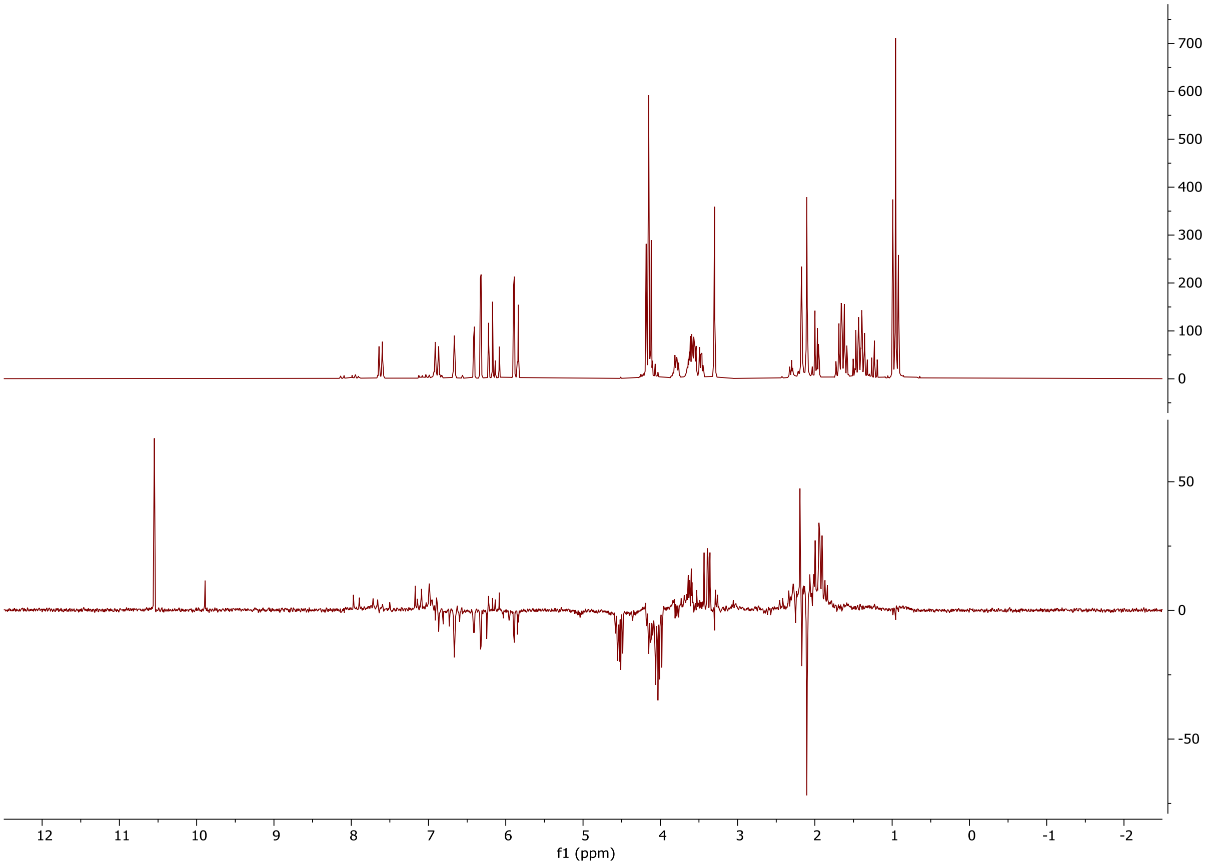

Figure S38. ^{1}H NMR (top) and photo-CIDNP (UV-vis lamp) spectra of **7** in Acetonitrile-d_3 in the presence of butyl acrylate.

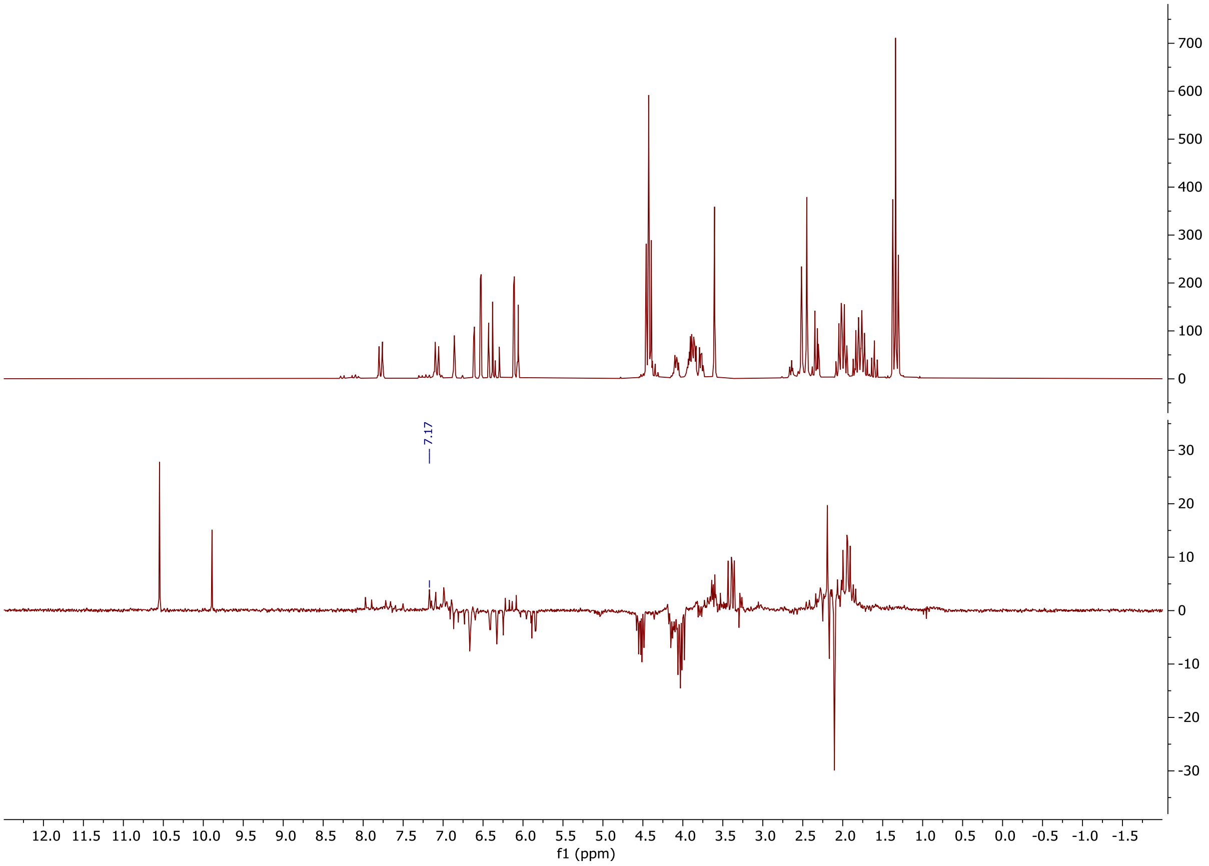

Figure S39. ^{1}H NMR (top) and photo-CIDNP (UV-vis lamp) spectra of **8** in Acetonitrile-d_3 in the presence of butyl acrylate.

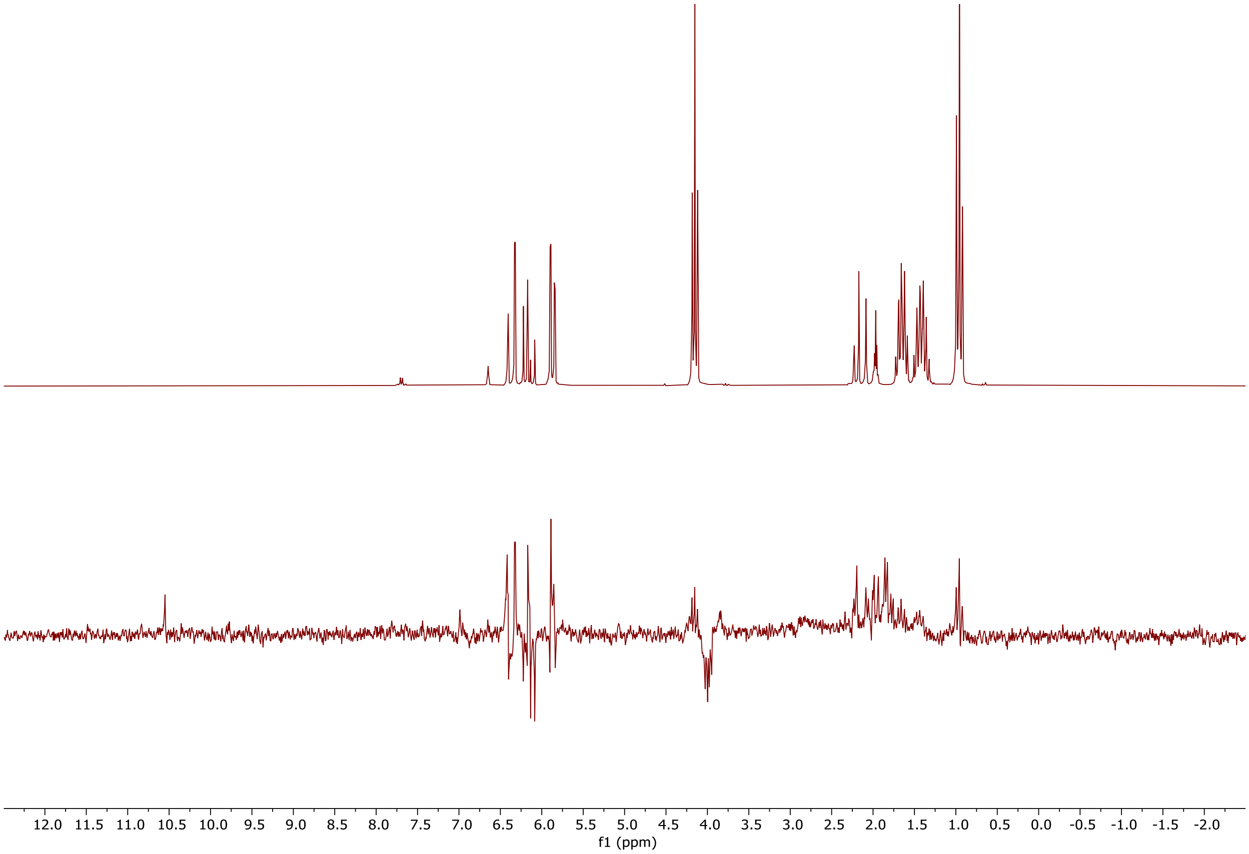

Figure S40. ^{1}H NMR (top) and photo-CIDNP (bottom) of **18** in the presence of Butyl acrylate in Acetonitrile-d_3. Only one aldehyde is present.

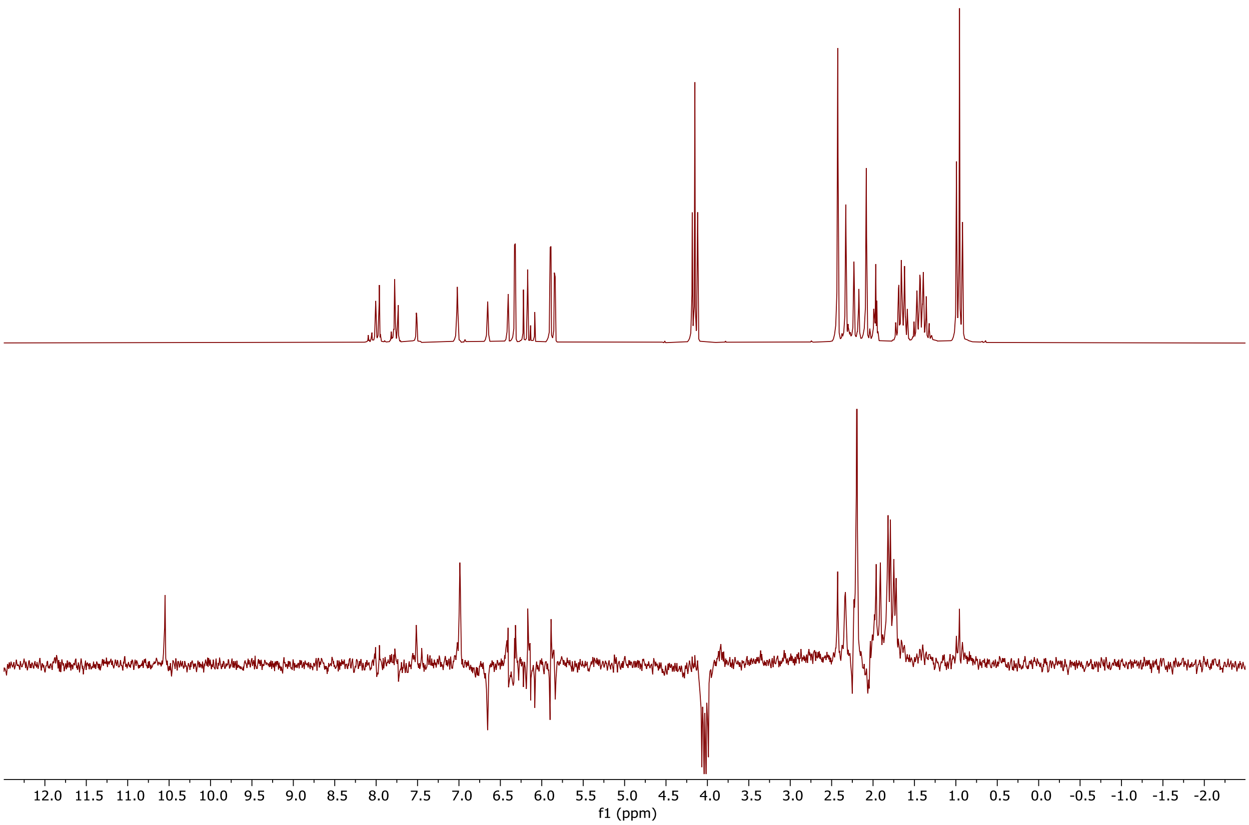

Figure S41. ^{1}H NMR (top) and photo-CIDNP (bottom) of **19** in the presence of Butyl acrylate in Acetonitrile-d$_3$. Only one aldehyde is present.

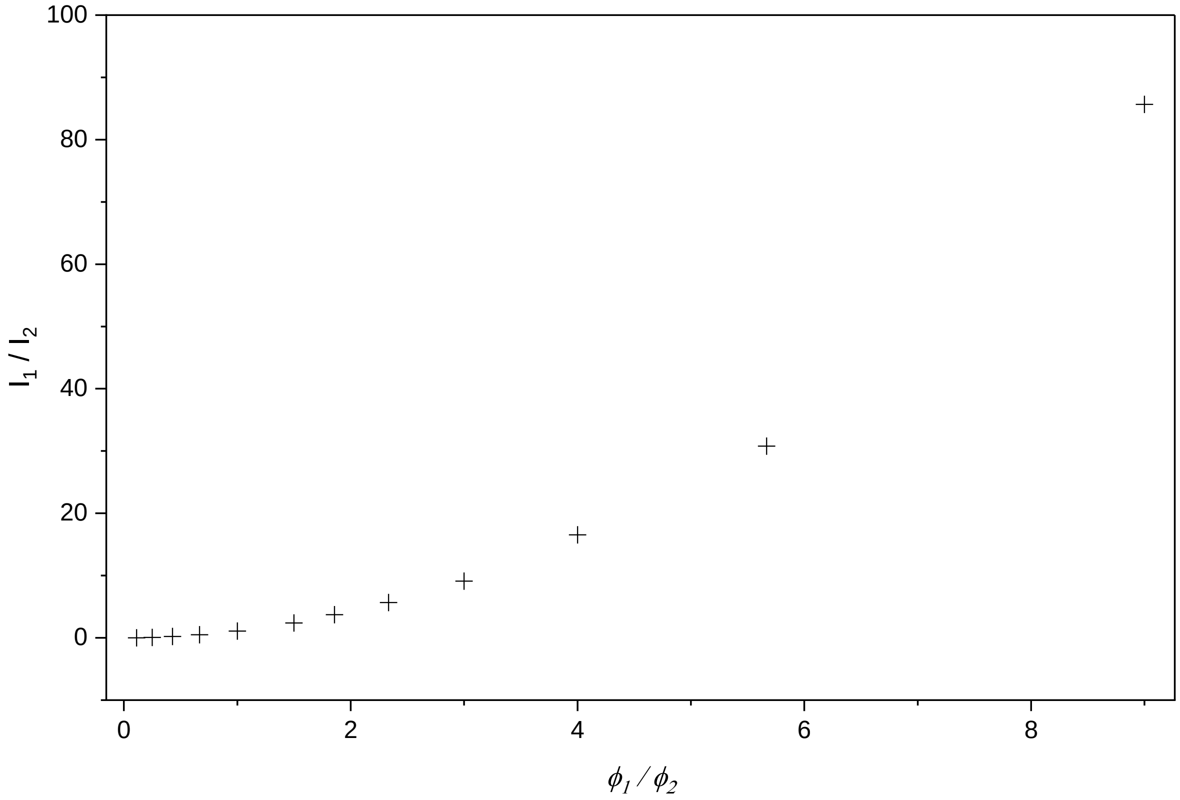

Figure S42. Ration of CIDNP polarizations of two aldehydes **15** and **16** (I_1/I_2) as the function of their quantum yields ratio (ϕ_1/ϕ_2) as evaluated by kinetic simulations with COPASI.[1] Kinetic equations correspond to the Scheme 5 of the main text. All other parameters were kept constant.

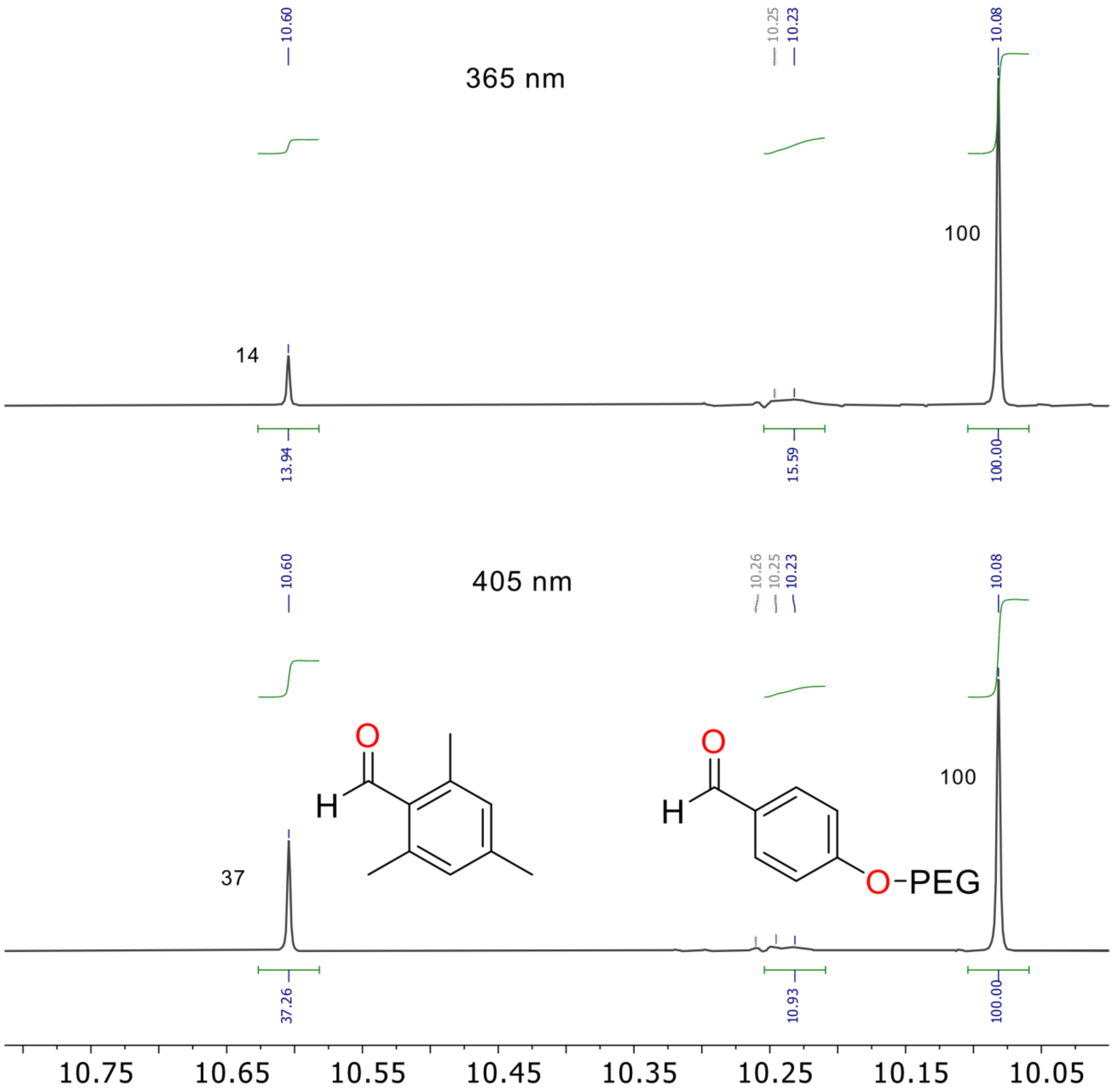

Figure S43. ^{1}H NMR (400 MHz) spectra of **8** in Acetonitrile-d_3 / benzyl alcohol mixture (10:6 v:v) after the irradiation at 365 nm (top) and 405nm. More than two-fold increase of mesitoyl aldehyde formation after the irradiation at 405 nm compared to 365 nm is clearly visible.

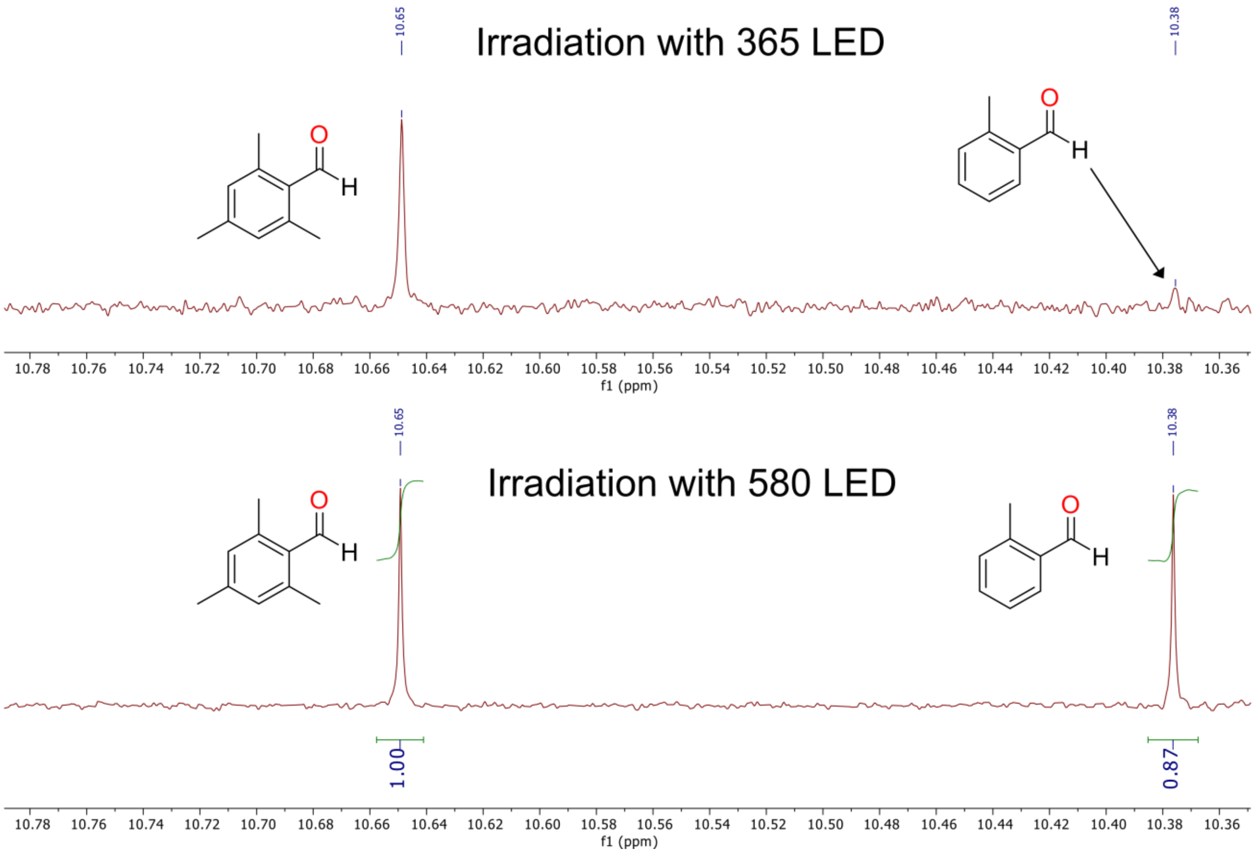

Figure S44. ^{1}H NMR after the irradiation of **20** at 365 nm (top) and 580 nm (bottom) in the presence of benzyl alcohol in Acetonitrile-d_3 (4:3, v:v)

Density Functional Theory Calculations

Computational Methods

The computation of the geometries and excited state properties of ivocerin® and compound **4** were started with the optimization on available crystal structures. For compound **8**, a model with a shorter chain (n=1) was included in the calculations. Density Functional Method (DFT) applying the B3LYP/def2-TZVP method [53,54] including Grimme's dispersion correction with Becke-Johnson damping D3BJ [55] was used for optimizations. Harmonic frequencies were computed to confirm that the structures are minima at the potential energy surface. The program Gaussian16 [56] was used for this part of the work. The solvent acetonitrile was modelled applying the conductor-like polarizable continuum model (CPCM) [57].

Twenty vertical excitations were then computed for the optimized geometries with the CAM-B3LYP functional [58] with the same basis set. Natural transition orbitals (NTOs) and NTO transition density differences were extracted from these data. The program ORCA6.0.1 [59] was used for this part of the work. For the simulation of the UV/Vis spectra, the program orca_asa [60] was used applying a Gaussian broadening of σ = 1500 cm^{-1}.

For the dissociation of the mesitoyl and of p-alkoxy-benzoyl moieties from **8**, we used fixed geometries at elongated Ge-C bonds starting from the optimized triplet state minimum and increasing the Ge-C distance from 1.9-7.0 Å. For these geometries, we computed CAM-B3LYP/def2-SVP single point energy curves applying the *FlipSpin* method with *FinalMs 1* to the described dissociating triplet state geometries to get the BrokenSym and HighSpin energies. Then, we performed for the same geometries single point TDDFT calculations using the keyword *triplets true* to get the singlet and triplet state energies. For this part of the study, we used the program ORCA6.0.1.

Computational results

The UV/vis spectra of ivocerin®, compound **4** and the model of **8** are discussed in the following. Detailed excitation data are given in Tables S1-S3, and the relevant natural transition orbitals (NTOs) are depicted in Tables S4-S6.

All three computed molecules show a long-wavelength band around 370 nm (computed value) assigned to n→π* transitions from the carbonyl groups and a shorter wavelength band at ca. 270-280 nm assigned to π→π* transitions at the aromatic moieties, respectively. Based on their structure with a different number of mesitoyl groups, the long wavelength band(s) are formed by two vertical excitations in ivocerin®, and three vertical excitations in compound **4** (see Tables S3 and S4) with similar energies. All excited states consist of a linear combination of the various n-π* transitions around the mesitoyl moieties.

Model compound **8** shows four distinct n→π* transitions responsible for the long-wavelength band. The first one of them with the lowest energy (S1) shows a transition mainly at the C=O groups of the mesitoyl groups, while the three with higher energies (S2, S3 and S4) additionally contribute transition densities at the C=O group at the benzoyl moieties. The respective transition density differences between the ground and excited state are sketched in Figure 3 in the main paper and presented in detail in Table S7 for compound **8**.

The simulation of the UV spectrum for the model of **8** shows a good agreement with the experimental spectrum (Figure S45) when shifting the spectra by 30 nm to account for the systematic error of the theoretical method.

Table S1: Natural transitions and the respective wavelength λ (in nm), oscillator strength f and excitation energy E_{exc} (in eV) of the first four transitions of ivocerin®, computed with CAM-B3LYP/def2-TZVP in acetonitrile. Orbital 103 is the HOMO, orbital 104 is the LUMO. Occupation numbers of the NTOs are given in brackets for $n \geq 0.10$.

**Excitation**	λ / nm	f	E_{exc}/ eV	NTOs (occupation number n)
S_1	374.2	0.0022	3.313	0.73 (103→104),<br>0.26 (102→105
S_2	370.7	0.0059	3.344	0.73 (103→104),<br>0.26 (102→105)
S3	260.6	1.1042	4.757	0.64 (103→104),<br>0.30 (102→105)
S_4	255.6	0.2816	4.851	0.68 (103→104),<br>0.24 (102→105)

Table S2: Relevant Natural Transition Orbitals (NTOs) of ivocerin®, computed with CAM-B3LYP/def2-TZVP in acetonitrile. Orbital 103 is the HOMO, orbital 104 is theLUMO. Contour values are 0.05 a.u. The individual geometries are slightly rotated for better visibility of the NTOs.

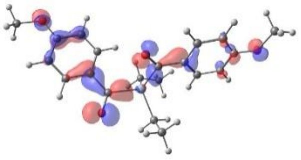	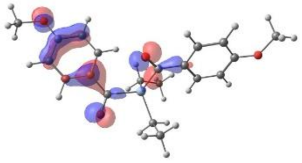
102	103
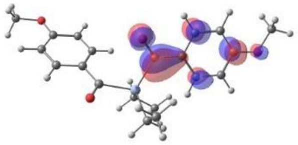	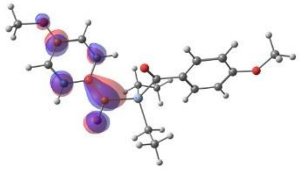
104	105

Table S3: Natural transitions and the respective wavelength λ (in nm), oscillator strength f and excitation energy E_{exc} (in eV) of the first five transitions of **4**, computed with CAM-B3LYP/def2-TZVP in acetonitrile. Orbital 174 is the HOMO, orbital 175 is the LUMO. Occupation numbers of the NTOs are given in brackets for n ≥ 0.10.

**Excitation**	λ / nm	f	E_{exc}/ eV	NTOs (occupation number n)
S_1	370.5	0.0023	3.347	0.69 (174→175),<br>0,28 (173→176)
S_2	363.3	0.0090	3.413	0.85 (174→175),<br>0.12 (173→176)
S_3	356.6	0.0119	3.477	0.54 (174→175),<br>0.38 (173→176)
S_4	271.1	0.0959	4.573	0.55 (174→175),<br>0.37 (173→176)
S_5	269.8	0.0514	4.596	0.93 (174→175)

Table S4: Relevant Natural Transition Orbitals (NTOs) of **4**, computed with CAM-B3LYP/def2-TZVP in acetonitrile. Orbital 174 is the HOMO, orbital 175 is the LUMO. Contour values are 0.05 a.u. The individual geometries are slightly rotated for better visibility of the NTOs.

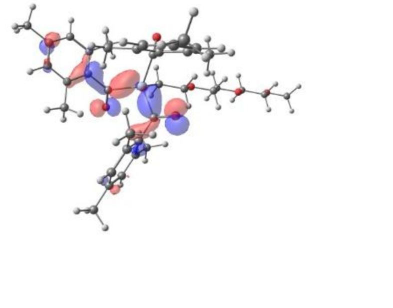	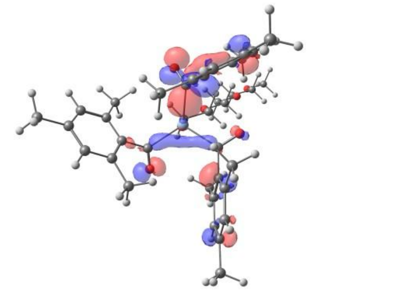
173	174
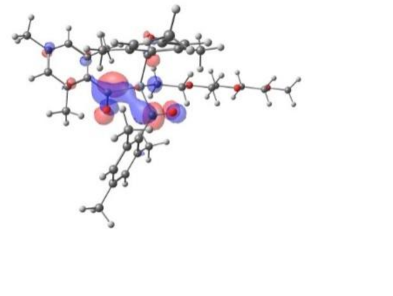	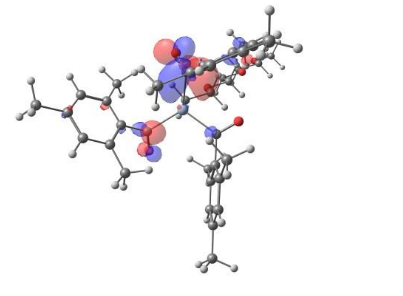
175	176

Table S5: Natural transitions and the respective wavelength λ(in nm), oscillator strength f and excitation energy Eexc (in eV) of the first six transitions of the model of **8**, computed with CAM-B3LYP/def2-TZVP in acetonitrile. Orbital 189 is the HOMO, orbital 190 is the LUMO. Occupation numbers of the NTOs are given in brackets for n≥ 0.10.

Excitation	λ / nm	f	E_{exc}/ eV	transition (occupation number n)
S_1	383.0	0.0051	3.237	189 → 190 (0.71)<br>188 → 191 (0.23)
S_2	371.2	0.0149	3.340	189 → 190 (0.67)<br>188 → 191 (0.19)<br>187 → 192 (0.10)
S_3	360.1	0.0119	3.443	189 → 190 (0.88)
S_4	358.4	0.0154	3.459	189 → 190 (0.56)<br>188 → 191 (0.30)<br>187 → 192 (0.13)
S_5	279.3	0.3563	4.439	189 → 190 (0.74)<br>188 → 191 (0.12)
S_6	276.5	0.1449	4.483	189 → 190 (0.74)<br>188 → 191 (0.18)

Table S6: Relevant Natural Transition Orbitals (NTOs) for **8** with n=1, computed with CAM-B3LYP/def2-TZVP in acetonitrile. Orbital 189 is the HOMO, orbital 190 is theLUMO. Contour values are 0.05 a.u. The individual geometries are slightly rotated for better visibility of the NTOs.

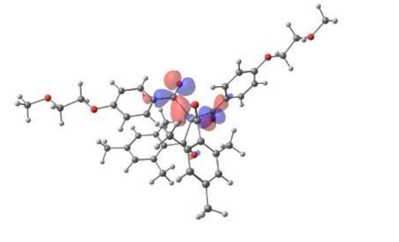	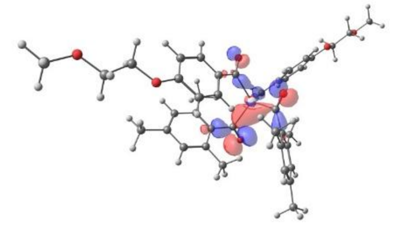	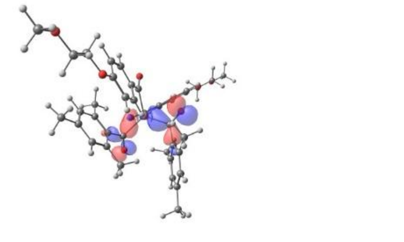
187	188	189
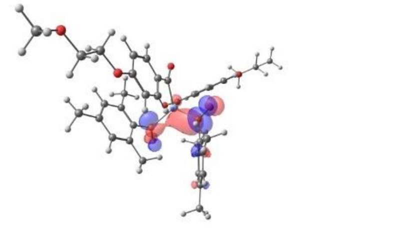	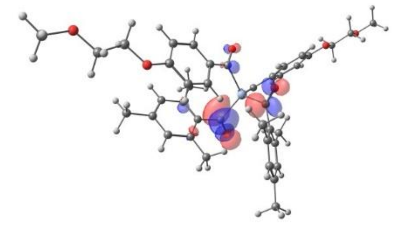	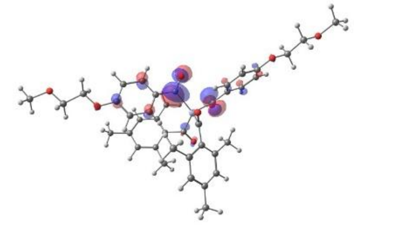
190	191	192

Table S7: Natural Transition Orbital (NTO) difference densities for vertical excitations S_1-S_6 of the model of **8**. The reduction of density is shown in red, the increase of density is shown in blue. The geometries are rotated in the individual pictures for better visibility of the NTO difference densities. Contour values of 0.005 a.u. were used in the program gabedit. The type of the main transition is printed as a comment together with the computed excitation energy.

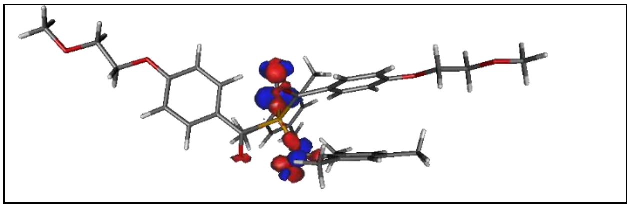	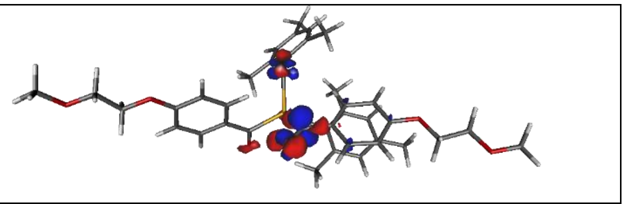
S_1: 3.237 eV, $n\rightarrow\pi^*$ at $C{=}O_{Mes+Mes}$	S_2: 3.340 eV, $n\rightarrow\pi^*$ at $C{=}O_{Bz+Mes}$
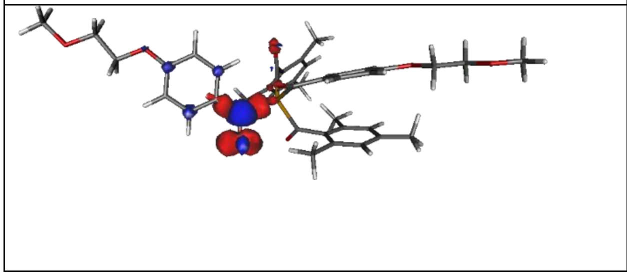	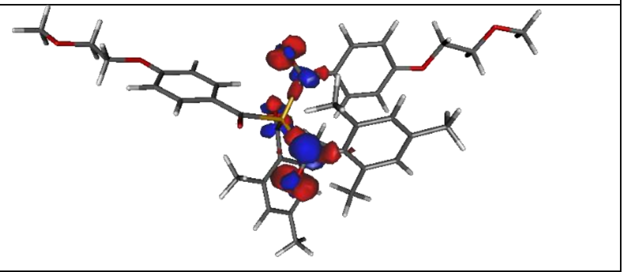
S_3: 3.443 eV, $n\rightarrow\pi^*$ at $C{=}O_{Bz}$	S_4: 3.459 eV, $n\rightarrow\pi^*$ at $C{=}O_{Mes+Bz+Mes}$
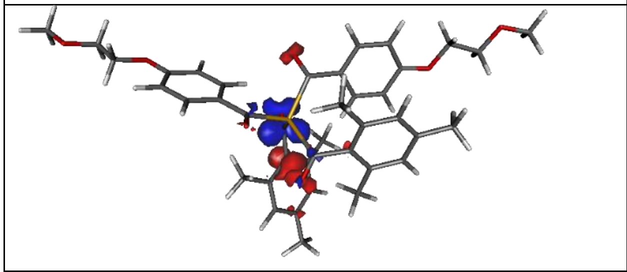	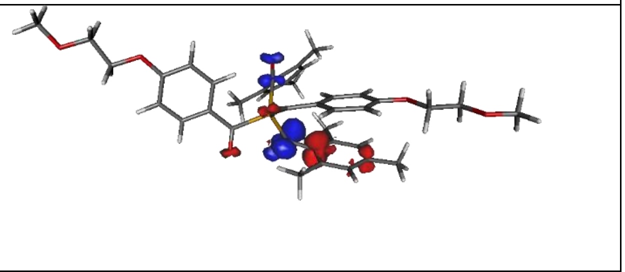
S_5: 4.439 eV, $\pi_{Mes}\rightarrow\sigma^*_{Ge}$	S_6: 4.483 eV, $\pi_{Mes}\rightarrow\pi^*_{C=O@Mes}$

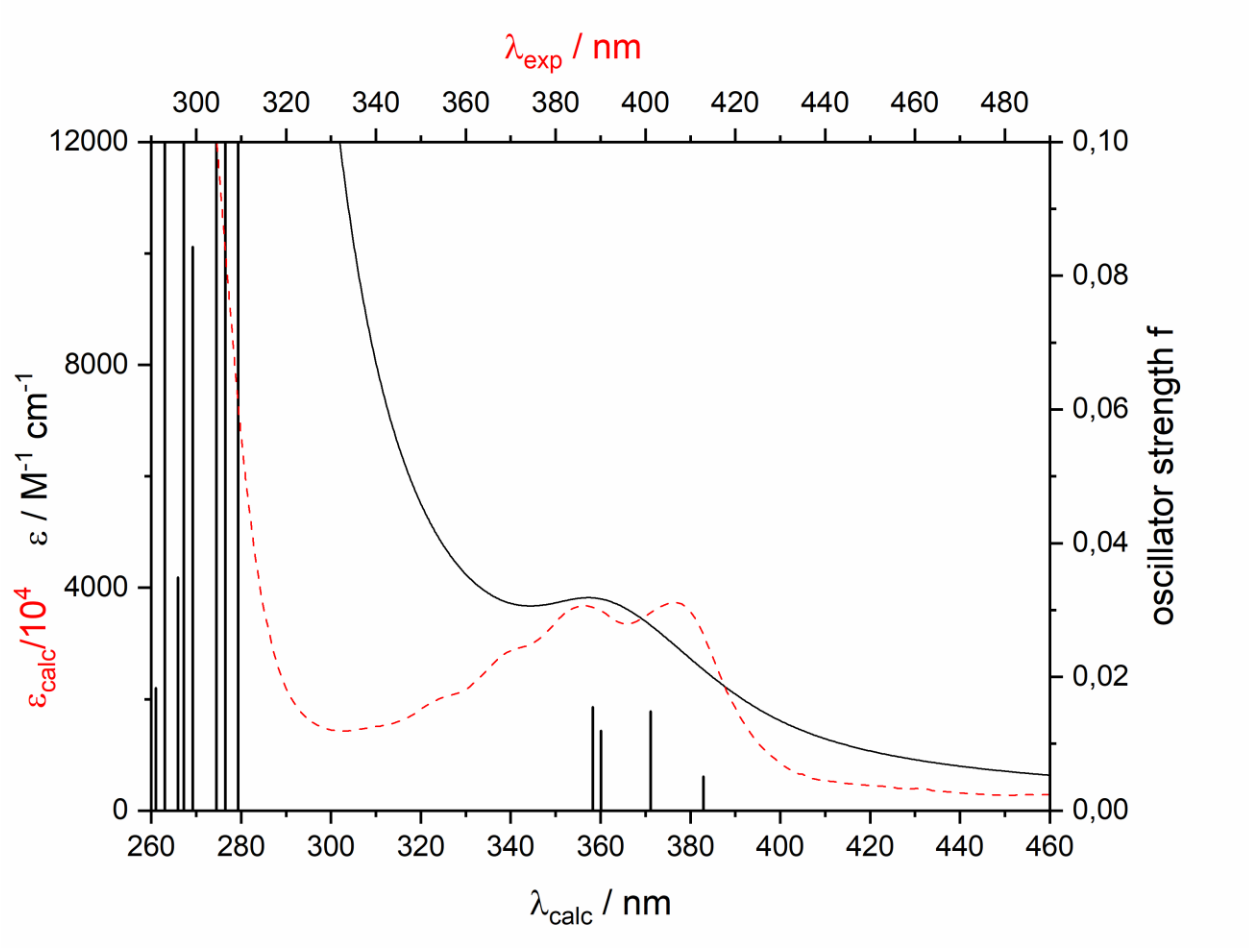

Figure S45. Simulated CAM-B3LYP/def2-TZVP/ACN absorption spectrum for **8** applying a Gaussian broadening (σ = 1500 cm^{-1}). The experimental (red) and theoretical (black) spectra are shifted by 30 nm relative to each other to account for the systematic error of the CAM-B3LYP/def2-TZVP/CPCM(acetonitrile) method. The left, red axis caption is valid for the experimental spectrum. The black vertical lines are the respective oscillator strengths.

Effect of the p-methoxy group to the orbitals and excited states.

To evaluate the effect of *para*-substitution on the benzene ring by an -O-PEG group, we additionally modelled compound **8** with a pristine benzoyl moiety lacking any *p*-substituent. This allows estimation of the influence of a methoxy (or -O-PEG) group on the absorption characteristics and electronic properties through inspection of the molecular orbitals, natural transition orbitals (NTOs), and excitation energies.

Molecular orbitals and transition densities in compound **8**, the frontier orbitals (HOMO-2 to LUMO+2) exhibit higher and more localized electron density at the carbonyl groups of the mesitoyl moieties when a methoxy group is present in the *para*-position of the benzoyl system (see Table S8), compared to the unsubstituted analogue (**8–without–O–PEG**). Thus, the *p*-methoxy (or -O-PEG) substituent donates electron density toward the carbonyl groups while simultaneously depleting it in the aromatic system of the benzene ring.

As a consequence, the first four n-π transitions are blue-shifted in 8 when the -O-PEG group is attached to the benzoyl moiety in para-position (Table S9). The most pronounced shifts are observed for transitions S2 and S3 (≈0.06 - 0.08 eV), while S1 and S4 are only slightly affected (≈0.001 - 0.01 eV). The transition densities (TDs) remain largely similar (noting that the coefficient applied for the natural transition densities shown in Fig. S45 was 0.005 a.u.). For S1, a minor redistribution is seen

at one of the benzoyl carbonyl groups; S2 and S3 display stronger TDs on the mesitoyl carbonyls, whereas S4 shows slightly enhanced TDs at the benzoyl C=O groups compared to the unsubstituted 8-without-O-PEG (cf. TD sketches in Fig. 3 of the main manuscript).

Table S8: Comparison of CAM-B3LYP/def2-TZVP/CPCM(acetonitrile) natural transition orbitals (NTOs for **8** and a model of **8** without o-PEG. The molecules are oriented so that the O=C-Bz-O-PEG moieties are looking to the right and bottom, the mesitoyl moieties are looking to top and left. We like to note that the order of some of the orbitals is interchanged, e.g. HOMO-1 and LUMO+1 of 8 is the same as HOMO and LUMO+2 of (**8**-without o-PEG), respectively, and vice versa.

orbital	8	8 without o-PEG
HOMO-2	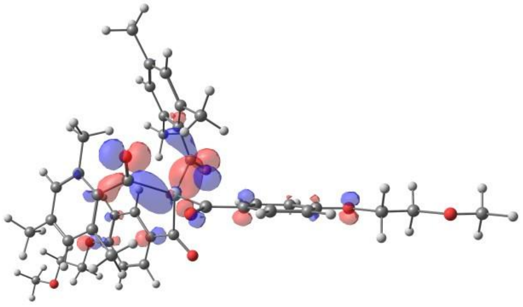	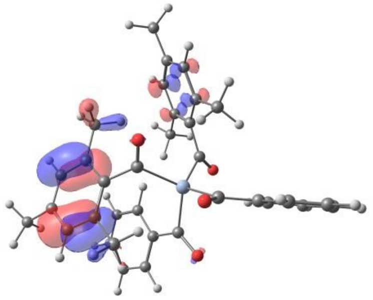
HOMO-1	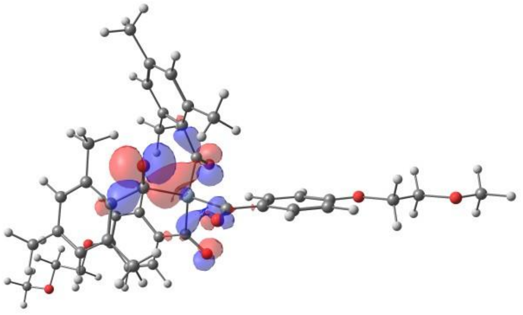	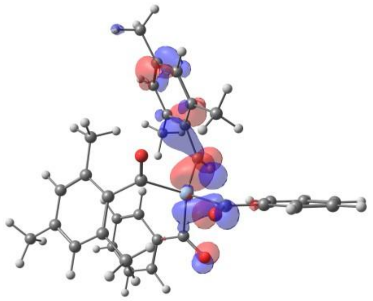
HOMO	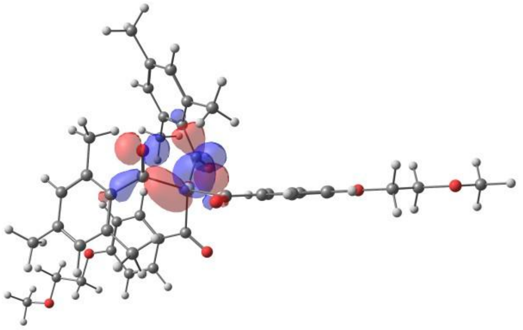	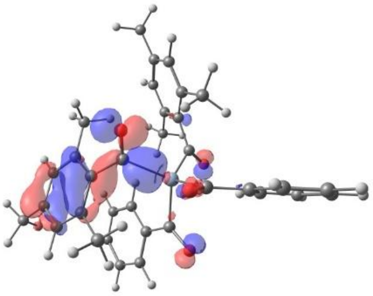

LUMO	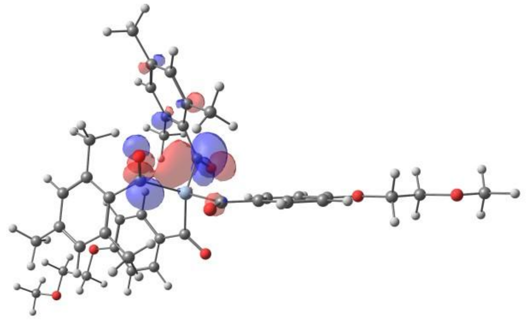	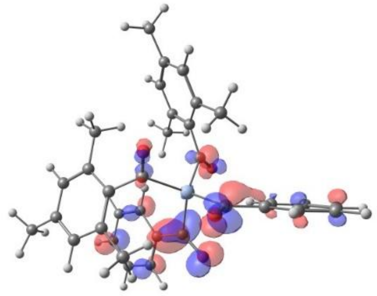
LUMO+1	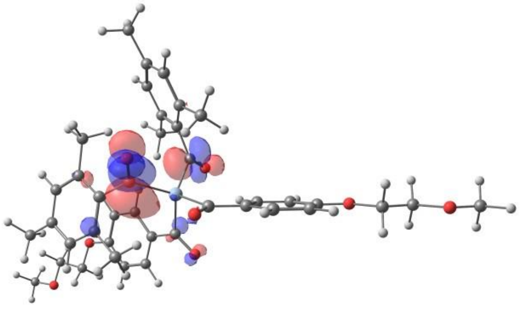	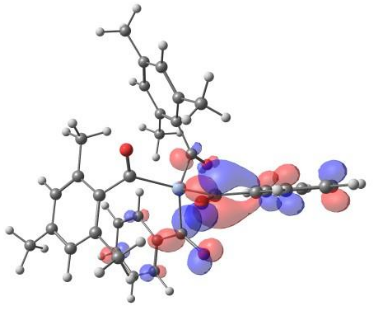
LUMO+2	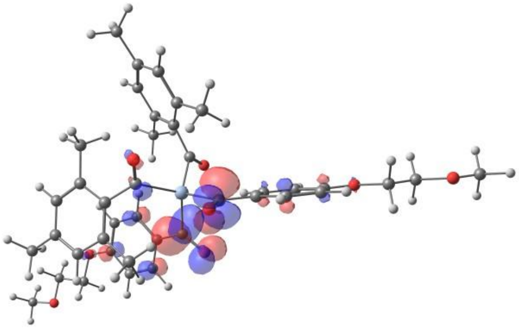	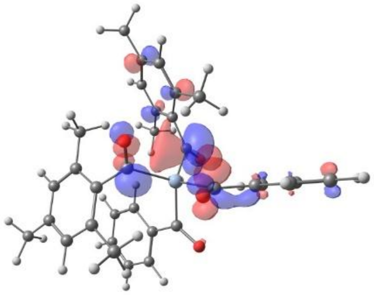

Table S9: Comparison of CAM-B3LYP/def2-TZVP/CPCM(acetonitrile) transition densities for **8** and a model of **8** without O-PEG. The molecules are oriented so that the O=C-Bz-O-PEG moieties are looking to the right and bottom, the mesitoyl moieties are looking to top and left.

state	8	8 without O-PEG
S1	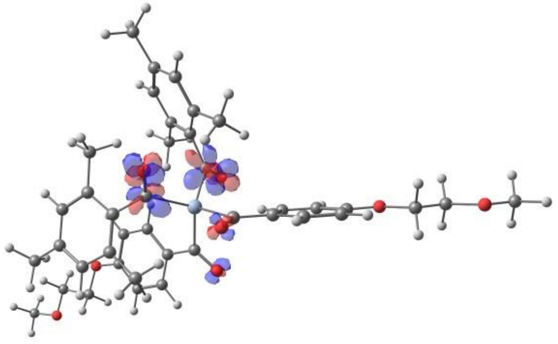<br>E_{exc} = 3.237 eV, λ = 383.0 nm	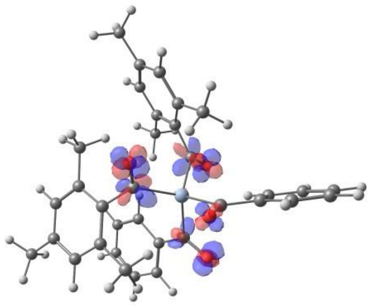<br>E_{exc} = 3.223 eV, λ = 384.7 nm
S2	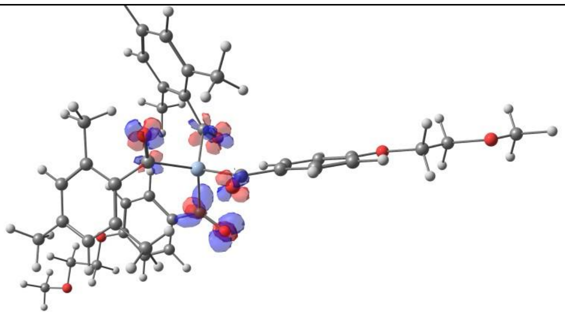<br>E_{exc} = 3.340 eV, λ = 371.2 nm	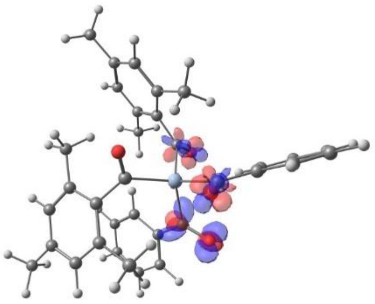<br>E_{exc} = 3.277 eV, λ = 378.7 nm
S3	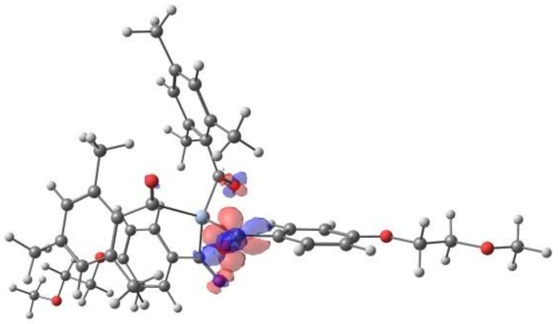<br>E_{exc} = 3.443 eV, λ = 360.1 nm	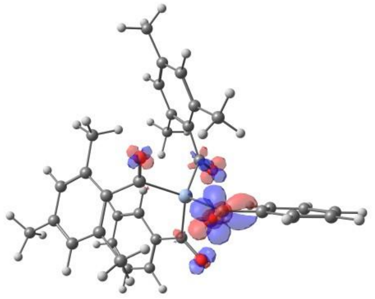<br>E_{exc} = 3.358 eV, λ = 369.2 nm

S4	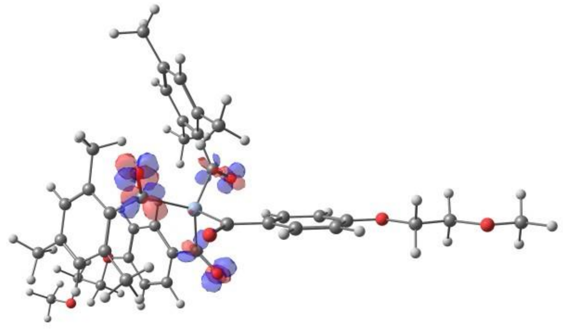 E_{exc} = 3.459 eV, λ = 358.9 nm	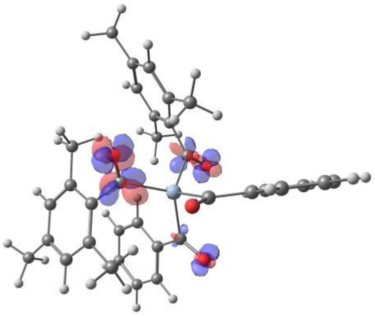 E_{exc} = 3.454 eV, λ = 358.9 nm

Table S10. Natural transitions and the respective wavelength (in nm), oscillator strenght f and excitation energy E_{exc} (in eV) of the first six transitions of the compound **8-without-O-PEG**, computed with CAM-B3LYP/def2-TZVP in acetonitrile. Orbital 149 is the HOMO, orbital 150 is the LUMO. Occupation numbers of the NTOs are given in brackets for n ≥ 0.10.

Excitation	λ / nm	f	E_{exc}/ eV	transition (occupation number n)
S_1	384.7	0.0010	3.233	149 → 150 (0.54)<br>148 → 151 (0.31)<br>147 → 152 (0.12)
S_2	378.3	0.0100	3.277	149 → 150 (0.61)<br>148 → 151 (0.28)<br>147 → 152 (0.10)
S_3	369.2	0.0113	3.358	149 → 150 (0.76)<br>148 → 151 (0.15)
S_4	358.9	0.0168	3.455	149 → 150 (0.64)<br>148 → 151 (0.28)
S_5	278.8	0.1967	4.447	149 → 150 (0.72)<br>148 → 151 (0.13)<br>147 → 152 (0.10)
S_6	277.7	0.0833	4.465	149 → 150 (0.68)<br>148 → 151 (0.23)

DFT computation of the triplet state dissociation:

Norrish type I photoinitiators dissociate from the low(est) triplet states after interconversion from higher states and intersystem crossing (ISC) between singlet and triplet states. The ISC and IC rates influence the processes after excitation, which are difficult to compute and beyond the level of DFT theory for appropriate results. Multireference methods would be necessary for appropriate rates, but this is not feasible for such large molecules. For example, ISC rates depend exponentially on the singlet-triplet gap,[61] and an error of 0.05 eV, which is far below the accuracy of DFT, can make the rate totally different.

Therefore, we tried to get some understanding of the excited state processes responsible for the two cleavage pathways of **8** after absorption by a simpler procedure. We performed TDDFT calculation on the fixed-geometry dissociation of **8** leading to R_1• (8C1•) + 8Ge1• or R_2• (8C2•) + 8Ge2•, respectively, in both their singlet and triplet states. With these calculations, we can support the experimentally detected variation of the product amounts for **14** and **15**.

First, we checked if the dissociating lowest T_1 triplet state has a barrier towards dissociation. Such barriers are usually small (compare the work of Glotz for bissilyl dione, $\Delta E_{T1,exp}$ = 27 kJ/mol, [62]), but of course they will be different for the two pathways and such influence the dissociation rates and product ratio. For this purpose, we computed the dissociation curves of the optimized triplet state of **8** by the *spinflip* method using DFT with regard to dissociating the mesitoyl R_1• (8C1•) or the p-alkoxybenzoyl R_2• (8C2•) groups, respectively.

Second, we wanted to estimate the excited states after absorption of the triplet state of **8** in more detail upon calculating the single point TDDFT curves of the lowest six singlet and triplet states. For this purpose, we computed the fixed-geometry Ge-C dissociation of both, the mesitoyl R_1• (8C1•) or the p-alkoxybenzoyl R_2• (8C2•) groups, respectively. With this procedure, we want to get an idea about the singlet-triplet splitting and possible ISC and IC pathways to explain the different product ratio upon excitation with light of different energy.

FlipSpin curves and the T_1 Barriers:

The FlipSpin curves (Figure S46) show that the triplet state dissociation of the mesitoyl group R_1• has a smaller barrier (27.0 kJ/mol) than the dissociation of the p-alkoxybenzoyl group R_2• (131.4 kJ/mol). Thus, we expect a higher rate constant for the dissociation of the mesitoyl group via the T_1 state. This finding supports our experimental study that the amount of **14** is higher than of **15**.

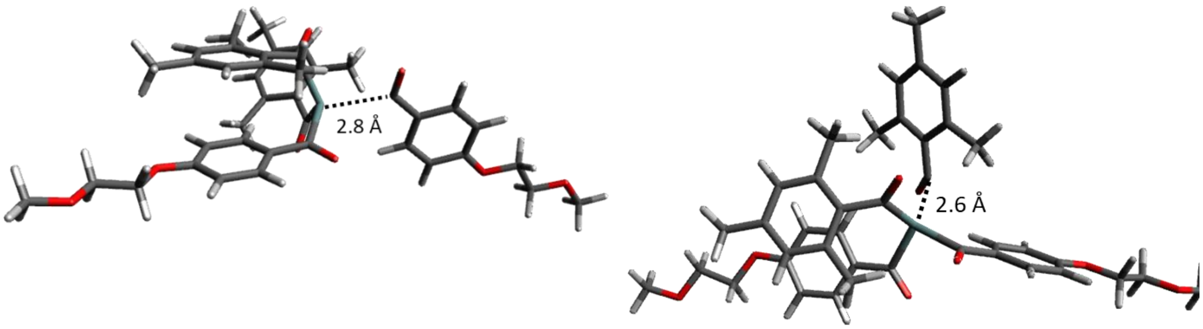

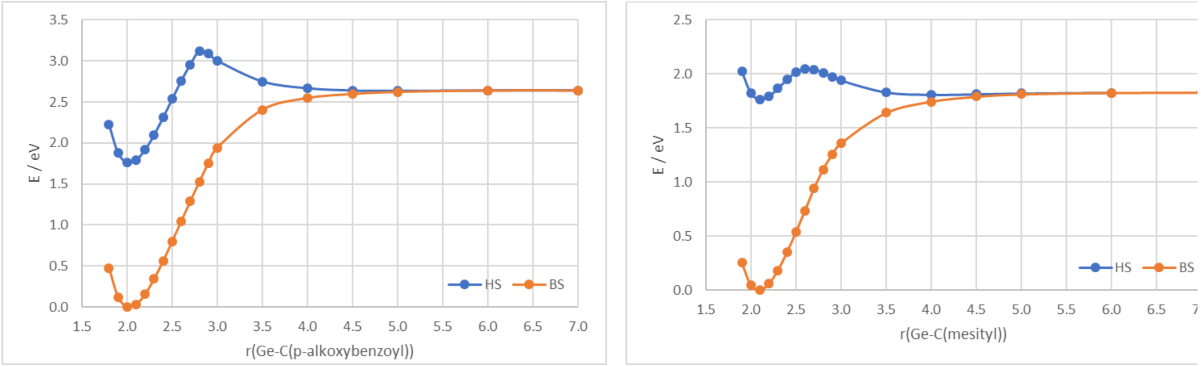

Figure S46. Geometries of the triplet state transition state (top) and SpinFlip dissociation curves of the triplet state of **8** (blue curve in the bottom graphs) upon dissociation of mesitoyl (left side) or of p-alkoxybenzoyl (right side) moiety. HS denotes the high spin triplet, BS denotes the broken symmetry singlet curve of the elongated triplet state geometries with fixed Ge-C distances.

Extended mechanistic interpretation based on excited-state topology

Fixed-geometry excited-state scans for **8** establish that both mesitoyl and p-alkoxybenzoyl release originate from triplet excited states, with mesitoyl dissociation remaining dominant across the investigated wavelength range due to its lower adiabatic T_1 dissociation barrier. Nevertheless, the experimentally observed increase in the relative contribution of p-alkoxybenzoyl release upon excitation at lower photon energies (longer wavelengths) cannot be rationalized on the basis of T_1 barrier heights alone and instead reflects pronounced differences in the excited-state topology along the two dissociation coordinates.

Along the p-alkoxybenzoyl dissociation coordinate, the energy separation $\Delta E(T_2 - T_1)$ is substantially smaller than for mesitoyl cleavage, and the T_2 surface exhibits a local minimum at approximately the Ge–C bond length where the T_1 surface reaches its maximum. This arrangement creates a region of strong nonadiabatic T_1/T_2 state mixing, allowing population approaching the T_1 bottleneck to transiently stabilize on T_2 and subsequently re-enter the T_1 surface on the product side. Such a two-triplet-state pathway effectively alleviates the dynamical bottleneck associated with the T_1 maximum, despite the higher adiabatic T_1 barrier for p-alkoxybenzoyl dissociation.

In contrast, the mesitoyl dissociation coordinate exhibits a significantly larger $\Delta E(T_2 - T_1)$ and lacks a comparable T_2 stabilization region near the T_1 maximum, resulting in a more direct, single-surface T_1 dissociation pathway. Although mesitoyl cleavage benefits from a lower adiabatic barrier, the absence of a similarly favorable multi-triplet topology limits the ability of excited-state population to exploit alternative nonadiabatic routes.

Additional differences arise from the relative placement of singlet–triplet crossings along the dissociation coordinates. For both reactions, crossings between S_1 and T_2 are identified; however, for p-alkoxybenzoyl dissociation this crossing occurs on the reactant side of the T_1 maximum, whereas for mesitoyl dissociation it appears only at or beyond the T_1 maximum. As a result, intersystem crossing from S_1 into T_2 can populate the advantageous T_2 region prior to the bottleneck for p-alkoxybenzoyl cleavage, while providing no comparable kinetic advantage for mesitoyl cleavage. In addition, a higher-lying singlet–triplet crossing between S_4 and T_7 is found exclusively along the p-alkoxybenzoyl coordinate close to the equilibrium Ge–C bond length and is accessible already at low excitation energies. This near–Franck–Condon crossing offers an early nonadiabatic doorway into the triplet manifold while largely preserving p-alkoxybenzoyl-centered electronic character, thereby efficiently feeding population into the low-lying triplet-state topology relevant for dissociation.

Finally, structural relaxation following bond cleavage further differentiates the two pathways. p-alkoxybenzoyl release leads to a substantially more planar Ge-centered radical than mesitoyl release, indicative of enhanced electronic delocalization and stabilization. This increased product stabilization is expected to reduce recrossing and further bias the effective branching in favor of p-alkoxybenzoyl dissociation once this pathway is accessed.

Taken together, these results demonstrate that the wavelength-dependent branching observed experimentally arises from a combination of excitation-energy-dependent population of chromophore-centered excited states, early and geometrically well-positioned singlet–triplet crossings, and a uniquely favorable low-lying triplet-state topology for p-alkoxybenzoyl dissociation. The reaction outcome is therefore governed by nonadiabatic excited-state topology and multi-surface dynamics rather than by adiabatic barrier heights alone, while the overall dominance of mesitoyl cleavage remains dictated by its intrinsically lower T_1 dissociation barrier.

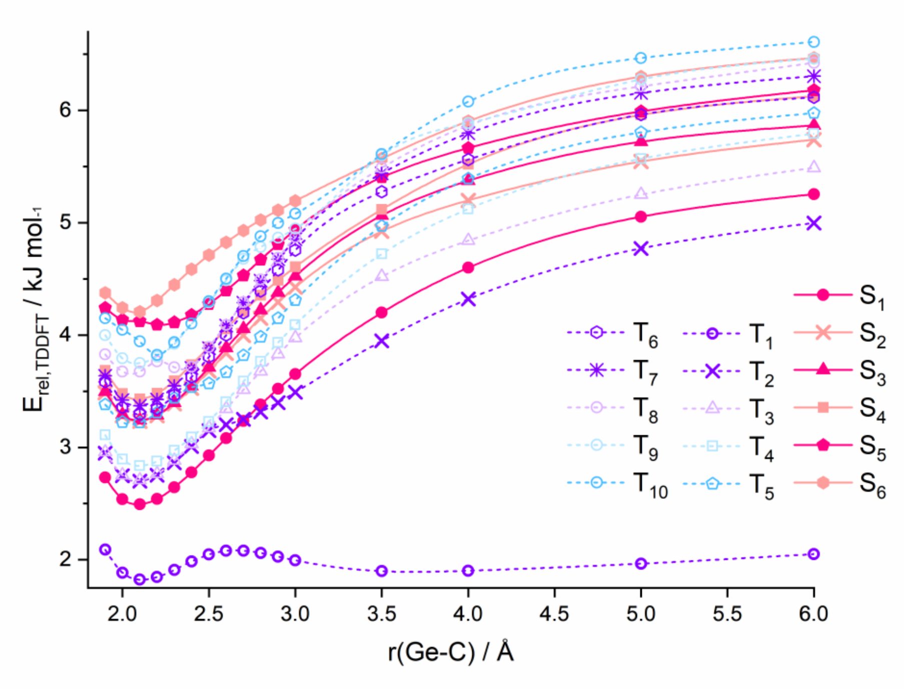

Figure S47. TDDFT dissociation curves of the singlet and triplet states of **8** upon elongation of the Ge-C bond (in Å) towards dissociation of mesitoyl R_1• (8C1•). Energies $E_{rel,TDDFT}$ are given relative to the optimized S_0 ground state of **8**. The Origin® data are available via the authors.

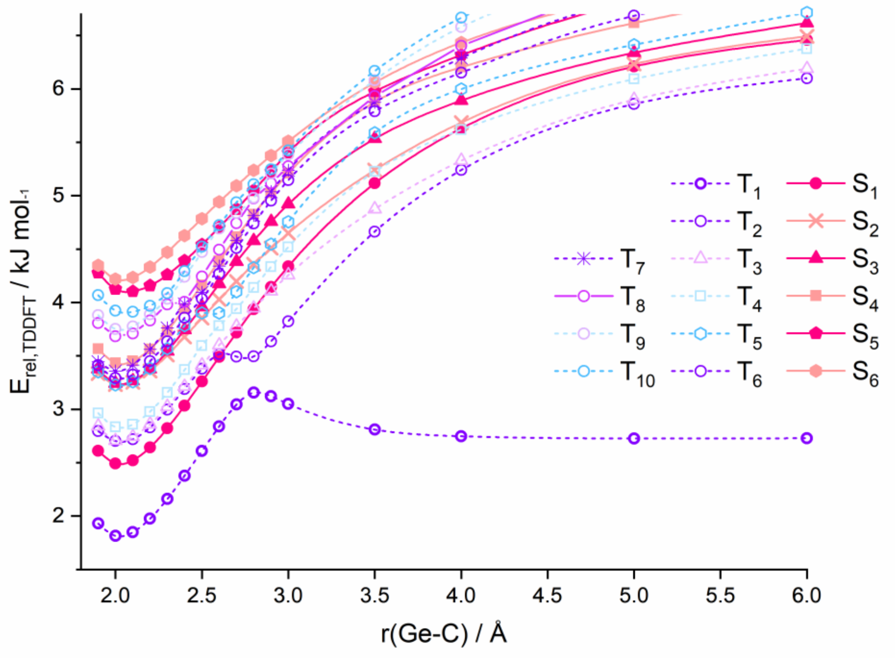

Figure S48. TDDFT dissociation curves of the singlet and triplet states of **8** upon elongation of the Ge-C bond towards dissociation to p-alkoxybenzoyl R_2• (8C2•). Energies $E_{rel,TDDFT}$ are given relative to the optimized S_0 ground state of **8**. The Origin® data are available via the authors.

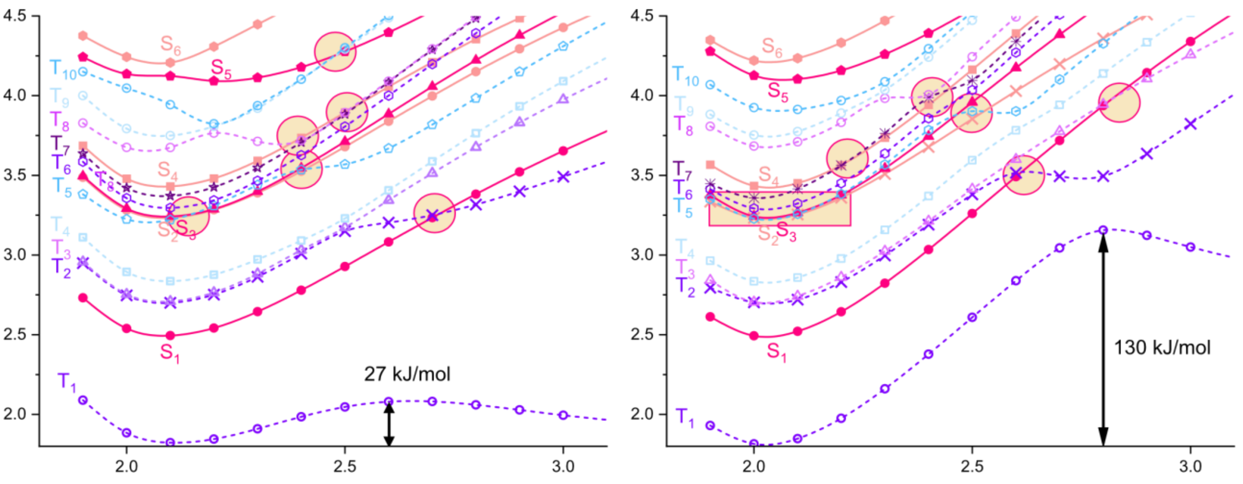

Figure S49. Zoomed-in / Schematic pictures of the singlet and triplet TDDFT dissociation curves of the triplet state of **8** upon elongation of the Ge-C bond towards dissociation to mesitoyl R_1• (8C1•) (left) and p-alkoxybenzoyl R_2• (8C2•) (right) moieties. Energies are given in kJ/mol based on CAM-B3LYP/def2-SVP/CPCM(acetonitrile) method. Important singlet-triplet crossings are marked with red circles.

Table S11: CAM-B3LYP/def2-SVP/CPCM(acetonitrile) computed data for the fixed-geometry Ge-C dissociation of **8**.

	R_1• (8C1•)+8Ge1•		R_2• (8C2•)+8Ge2•	
	E_{rel} / kJ/mol	r(Ge-C_R) / Å	E_{rel} / kJ/mol	r(Ge-C_R) / Å
T_1 barrier	24.9 [(a)]	2.7	129.4 [(a)]	2.8
	27.0 [(b)]	2.7	131.4 [(b)]	2.8
T_2 barrier	--	--	76.8 [(a)]	2.6
$\Delta E_{TDDFT}(S_1-T_1)$	64.8	2.1	65.4	2.0
ΔE_{TDDFT} (T_2-S_1)	65.5	2.0	19.9	2.0
ΔE_{TDDFT} $(T_2/S_1 – T_1)$	112.8	2.7	--	--
ΔE_{TDDFT} $(T_{2, min2} – T_{1,TS})$	--	--	32.6	2.8
	E_{rel} / kJ/mol	E_{rel} / eV		
E_{TDDFT} (T_1^{min})	175.8	1.8225		
E_{TDDFT} (S_1^{min})	240.66	2.4943		
E_{TDDFT} (T_2^{min})	260.78	2.7028		
E_{TDDFT} (S_2^{min})	312.87	3.2426		

(a) TDDFT single point calculation; (b) Spinflip calculation (HS value).

Single Crystal X-ray Crystallography

All crystals suitable for single crystal X-ray diffractometry were removed from a vial and immediately covered with a layer of silicone oil. A single crystal was selected, mounted on a MiTeGen on a copper pin, and placed in the cold N_2 stream provided by an Oxford Cryosystems cryostream. Data for crystal structures were collected using a Rigaku XtaLAB Synergy, Dualflex, HyPix-Arc 100 diffractometer. Data were measured using Cu Kα radiation (λ= 1.54056 Å). The diffraction pattern was indexed and the total number of runs and images was based on the strategy calculation from the program CrysAlisPro.[63] The unit cell was refined and data reduction, scaling and absorption corrections were performed using CrysAlisPro. Using Olex2, [64] the structure was solved with the SHELXT[65] structure solution program and refined with the SHELXL[66] refinement package using full matrix least squares minimization on F^2. The absolute configuration was established by anomalous dispersion effects in the diffraction measurements on the crystal. All non-hydrogen atoms were refined anisotropically. All hydrogen atoms were placed in calculated positions corresponding to standard bond lengths and angles and refined using a riding model.

CCDC deposition numbers 2424436 (compound **7**) and 2424437 (compound **6b**) contain the supplementary crystallographic data for compound in this paper. These data can be obtained free of charge via https://www.ccdc.cam.ac.uk/data_request/cif.

Table S12: *Crystallographic data and details of measurements for compounds* ***6b*** *and* ***7****. R1= Σ/ |F_o|- |F_c|/|Σ|F_d; wR2 = [$Σ_w$(F_o^2-F_2^2)^2/$Σ_w$(F_o^2)^2]$^{1/2}$*

**Compound**	**6b**	**7**
Formula	$C_{14}H_{20}O_6$	$C_{44}H_{52}GeO_8$
$D_{calc.}$/ g cm^{-3}	1.338	1.286
m/mm^{-1}	0.877	1.448
Formula Weight	284.30	781.44
Colour	colourless	yellow
Shape	plate	block-shaped
Size/mm^3	0.16×0.11×0.02	0.08×0.05×0.04
T/K	100.01(18)	99.9(9)
Crystal System	monoclinic	triclinic
Space Group	*P*2$_1$/*c*	*P*-1
a/Å	22.0671(5)	11.8530(3)
b/Å	7.59107(17)	12.5611(3)
c/Å	8.4443(2)	15.4157(3)
a/°	90	100.157(2)
b/°	93.782(2)	108.030(2)
g/°	90	105.430(2)
V/Å^3	1411.46(6)	2018.29(9)
Z	4	2
Z'	1	1
Wavelength/Å	1.54184	1.54184
Radiation type	Cu K_a	Cu K□
Q_{min}/°	4.015	3.143
Q_{max}/°	78.810	80.451
Measured Refl.	29390	33197
Independent Refl.	3012	8562
Reflections with I > 2(I)	2625	6761
R_{int}	0.0530	0.0590
Parameters	186	701
Restraints	0	252
Largest Peak	0.264	0.535
Deepest Hole	-0.311	-0.649
GooF	1.027	1.026
wR_2 (all data)	0.0975	0.1308
wR_2	0.0940	0.1209
R_1 (all data)	0.0441	0.0688
R_1	0.0378	0.0510

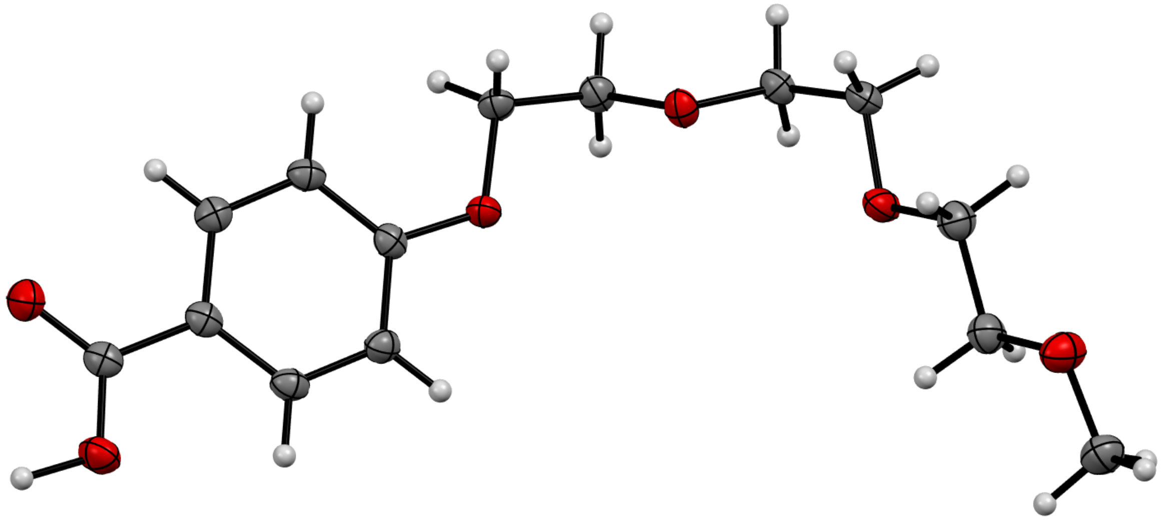

Figure S50. ORTEP representation of compound **6b**. Thermal ellipsoids are drawn at the 50% probability level.
